# Centromere-Specific Retrotransposons and Very-Long-Chain Fatty Acid Biosynthesis in the Genome of Yellowhorn (*Xanthoceras sorbifolium*, Sapindaceae), an Oil-Producing Tree With Significant Drought Resistance

**DOI:** 10.3389/fpls.2021.766389

**Published:** 2021-11-22

**Authors:** Hui Liu, Xue-Mei Yan, Xin-rui Wang, Dong-Xu Zhang, Qingyuan Zhou, Tian-Le Shi, Kai-Hua Jia, Xue-Chan Tian, Shan-Shan Zhou, Ren-Gang Zhang, Quan-Zheng Yun, Qing Wang, Qiuhong Xiang, Chanaka Mannapperuma, Elena Van Zalen, Nathaniel R. Street, Ilga Porth, Yousry A. El-Kassaby, Wei Zhao, Xiao-Ru Wang, Wenbin Guan, Jian-Feng Mao

**Affiliations:** ^1^National Engineering Laboratory for Tree Breeding, Beijing Advanced Innovation Center for Tree Breeding by Molecular Design, Key Laboratory of Genetics and Breeding in Forest Trees and Ornamental Plants, Ministry of Education, School of Ecology and Nature Conservation, College of Biological Sciences and Technology, Beijing Forestry University, Beijing, China; ^2^Protected Agricultural Technology, R&D Center, Shanxi Datong University, Datong, China; ^3^Key Laboratory of Plant Resources, Institute of Botany, Chinese Academy of Sciences, Beijing, China; ^4^Department of Bioinformatics, Ori (Shandong) Gene Science and Technology Co., Ltd., Weifang, China; ^5^Key Laboratory of Forest Ecology and Environment of the National Forestry and Grassland Administration, Research Institute of Forest Ecology, Environment and Protection, Chinese Academy of Forestry, Beijing, China; ^6^Umeå Plant Science Centre, Department of Plant Physiology, Umeå University, Umeå, Sweden; ^7^Départment des Sciences du Bois et de la Forêt, Faculté de Foresterie, de Géographie et de Géomatique, Université Laval Québec, Quebec City, QC, Canada; ^8^Department of Forest and Conservation Sciences, Faculty of Forestry, University of British Columbia, Vancouver, BC, Canada; ^9^Department of Ecology and Environmental Science, Umeå Plant Science Centre, Umeå University, Umeå, Sweden

**Keywords:** yellowhorn, centromere, LINE1, *Gypsy*, very-long-chain fatty acid

## Abstract

In-depth genome characterization is still lacking for most of biofuel crops, especially for centromeres, which play a fundamental role during nuclear division and in the maintenance of genome stability. This study applied long-read sequencing technologies to assemble a highly contiguous genome for yellowhorn (*Xanthoceras sorbifolium*), an oil-producing tree, and conducted extensive comparative analyses to understand centromere structure and evolution, and fatty acid biosynthesis. We produced a reference-level genome of yellowhorn, ∼470 Mb in length with ∼95% of contigs anchored onto 15 chromosomes. Genome annotation identified 22,049 protein-coding genes and 65.7% of the genome sequence as repetitive elements. Long terminal repeat retrotransposons (LTR-RTs) account for ∼30% of the yellowhorn genome, which is maintained by a moderate birth rate and a low removal rate. We identified the centromeric regions on each chromosome and found enrichment of centromere-specific retrotransposons of LINE1 and *Gypsy* in these regions, which have evolved recently (∼0.7 MYA). We compared the genomes of three cultivars and found frequent inversions. We analyzed the transcriptomes from different tissues and identified the candidate genes involved in very-long-chain fatty acid biosynthesis and their expression profiles. Collinear block analysis showed that yellowhorn shared the gamma (γ) hexaploidy event with *Vitis vinifera* but did not undergo any further whole-genome duplication. This study provides excellent genomic resources for understanding centromere structure and evolution and for functional studies in this important oil-producing plant.

## Introduction

Centromeres are those chromosomal regions that interact with spindle microtubules for the correct segregation of sister chromatids during mitosis and meiosis II, and of homologous chromosomes during meiosis I in eukaryotes (Houben and Schubert, 2003). Despite the early cytological discovery and rapid growth in the number of sequenced genomes, centromeres have been one of rather mysterious parts of genomes due to their highly repetitive content. Its function for chromosome segregation is highly conserved among species, but the sequences specific to centromeric chromatin are evolving rapidly, which is referred to centromere paradox (Henikoff et al., 2001). Satellite DNA is one of the dominant centromeric sequences in most species (Csink and Henikoff, 1998). Additionally, centromeric retrotransposons are found common in the centromeres of *Triticum boeoticum* and *Zea mays* (Zhong et al., 2002; Liu et al., 2008). In maize, centromeric retrotransposons include a lineage of *Gypsy* retrotransposons (Neumann et al., 2011) while, in *Musa acuminata*, they are dominated by long interspersed nuclear elements (LINE) and *Gypsy* (D’Hont et al., 2012; Čížková et al., 2013; Belser et al., 2021). The few available reports illustrate that sequence composition in centromeres can be complex and vary among species. However, our understanding of centromere structure, sequence composition, and the mode and the rate of evolution is thus far very limited.

Determining the precise boundaries of centromeres has proved to be difficult, especially for the repeat-rich plant genomes, creating challenges for complete genome assembly (Kumar and Bennetzen, 1999; Henikoff et al., 2001; Feschotte et al., 2002). The advance in long-read sequencing, such as Pacific Biosciences (PacBio) sequencing, and genome scaffolding methods, such as optical mapping and Hi-C sequencing, has vastly improved our ability to obtain unprecedented complete and contiguous genome assemblies (Sedlazeck et al., 2018). Long-read sequencing is also able to yield contiguous centromeric sequences and thus assemblies of centromeric regions despite their complex repeat structures (VanBuren et al., 2015; Belser et al., 2021). Based on the colocalization of centromeres and the patterns it creates in Hi-C contact maps, it is possible to infer the locations of all centromeres for all chromosomes in a genome (Mizuguchi et al., 2014; Varoquaux et al., 2015).

**FIGURE 1:**
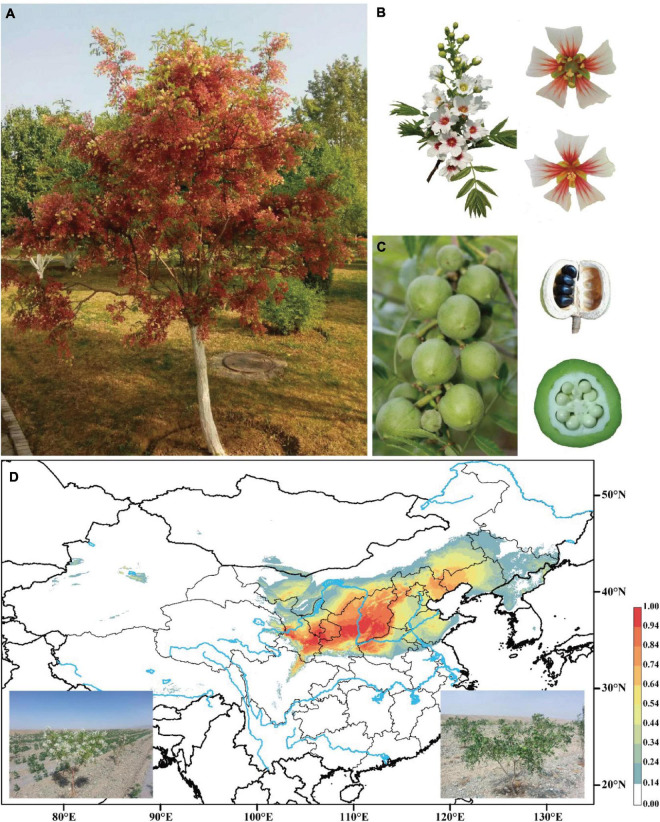
Images of yellowhorn and its potential distribution range. **(A)** The mature tree (“JGXP”) sampled for genome sequencing. **(B)** Raceme, hermaphrodite flower (up), and male flower (down). **(C)** Capsular fruits, seeds in ripe fruit, and cross-section of fruit. **(D)** Predicted distribution of yellowhorn based on sampled records and current climate data. Images at the bottom are the yellowhorn tree with flowers (left) and fruits (right), respectively.

Yellowhorn (*Xanthoceras sorbifolium*) is a rare, deciduous tree or shrub in the Sapindaceae family and the only species in the genus *Xanthoceras* native to dryland in northern China (Figures 1A–D). This species has a high capacity of saline-alkali tolerance and withstands extreme temperatures. It is thus widely used for afforestation programs for soil and water conservation (Yu et al., 2017). The seeds of yellowhorn are rich in lipids, proteins, and saponins, with oil contents range from 49.8% to 68.3% and unsaturated fatty acids up to 90.9% of the total fatty acids (Yao et al., 2013; Venegas-Calerón et al., 2017; Yu et al., 2017), and thus the plant has been identified as an important biofuel crop. Notably, nervonic acid, a very-long-chain fatty acid (VLCFA), which is rarely found in plants, accounts for 1.5-3% of the seed oil of yellowhorn (Ruan et al., 2017). Nervonic acid is an important component in myelin biosynthesis in the central and peripheral nervous system and an essential nutrient for brain growth and maintenance (Oda et al., 2005; Amminger et al., 2012). The increase of nervonic acid content in seeds will become an important target for yellowhorn breeding.

Previous morphometric analysis has determined the chromosome number of yellowhorn and the karyotype as 2n = 30 (Lang et al., 1980). Recently, two long-read-based genome assemblies have been reported for yellowhorn, each representing a different cultivar (Table 1; Bi et al., 2019; Liang et al., 2019). Genome analyses from these two studies show that yellowhorn diverged from its close sister species *Dimocarpus longan* at ∼33 MYA to ∼46 MYA (million years ago), and no whole-genome duplication event is detected in yellowhorn (Bi et al., 2019; Liang et al., 2019). However, the identification of centromere regions and their sequence characteristics, genome structural variations, and the biosynthesis of VLCFA were not investigated.

**TABLE 1:** Statistics of the three yellowhorn assemblies of “JGXP,” “ZS4,” and “WF18”. N50, shortest sequence length at 50% of the genome.

	JGXP	ZS4	WF18
Whole genome sequencing reads	PacBio and Illumina	PacBio and Illumina	PacBio, 10× Genomics, and Illumina
Scaffolding sequencing techniques	Hi-C	Hi-C	Hi-C and BioNano optical maps
Estimated genome size (Mb)	435^a^	526^c^/541^c^	434^c^/442^d^
Heterozygosity (%)	0.51^a^/0.38^b^	0.75^d^	0.81^d^
Number of chromosomes	15	15	15
Assembled genome size (Mb)	470	504	440
Anchored size (Mb)	446 (94.9%)	489 (97.0%)	420 (95.4%)
Number of scaffolds	988	2,297	267
N50 of scaffolds (Mb)	30.8	32.2	29.4
Number of contigs	3,302	2,836	2,002
N50 of contigs (Mb)	0.42	1.04	0.64
GC content (%)	34.94	36.95	32.75
Protein-coding genes	22,049	24,672	21,059/22,046^b^
TE proportion (%)	65.7	65.0	61.5
Complete BUSCOs	1361 (94.5%)	1,364 (94.7%)	1,218 (84.6%)
LAI	14.53	12.89	14.00

*LAI, LTR assembly index.*

*^a,d^Estimated by K-mer analysis using PacBio long reads and Illumina paired-end reads, respectively.*

*^b^Estimated using Illumina paired-end reads and values are retrieved from the study (Liang et al., 2019).*

*^c^Estimated by flow cytometry analysis.*

Here, we present a high-contiguity chromosome-level genome assembly for another cultivar of yellowhorn by combining PacBio long-reads and Hi-C scaffolding strategies. This high-quality genome assembly allowed us to identify the centromeric regions (Note that the term “centromeric” is used in this study to refer to both the centromeric and pericentromeric regions, as these are difficult to distinguish from one another) for the 15 chromosomes and characterize their sequence composition and mode of evolution. We further conducted comparative genomic analyses among cultivars and transcriptome analyses to identify candidate genes of VLCFA biosynthesis. The genome resources and investigations presented here enrich our understanding about centromere genetics and promote efficient utilization of this precious bio-resource plant.

## Results

### Genome Sequencing and Assembly

**FIGURE 2:**
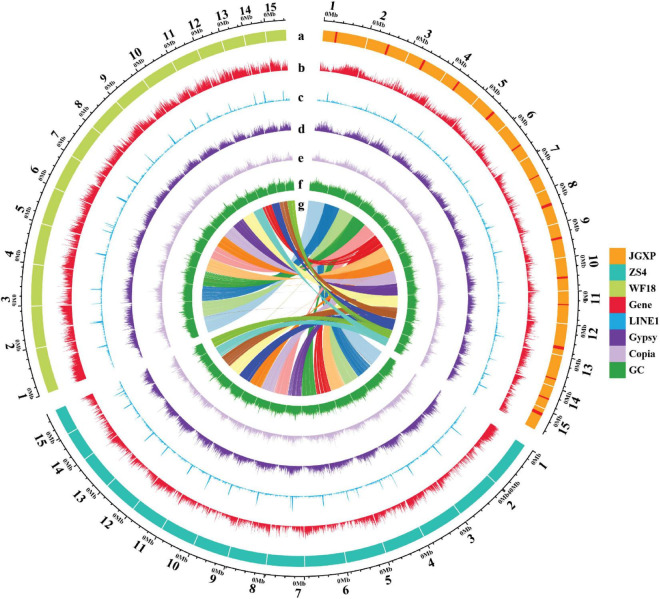
The genomic landscape across chromosomes among the three yellowhorn assemblies of “JGXP,” “ZS4,” and “WF18.” a: The tracks represent 15 assembled chromosomes for each genome of “JGXP,” “ZS4,” and “WF18.” The red rectangles in “JGXP” represent the centromeric regions. b–f: The distribution of the gene density, LINE1 density, *Gypsy* density, *Copia* density, and GC content, respectively, with densities calculated in 100 Kb non-overlap windows. g: The homolog chromosomes among three cultivars of yellowhorn. The chromosomes of “JGXP” are used as references.

A nationally certificated variety, “Jinguanxiapei” (“JGXP”) (Figure 1A), was selected to generate ∼60 Gb (∼120×) PacBio long reads, ∼60 Gb (∼120×) Hi-C reads, and ∼21 Gb (∼40×) Illumina paired-end reads (Supplementary Tables 1, 2) for *de novo* genome assembly. The genome size and heterozygosity were estimated to be 435 Mb and 0.51%, respectively, based on 17-bp *K*-mers frequency analysis with corrected PacBio long reads (Table 1 and Supplementary Figure 1). The total assembly length of “JGXP” was 470 Mb with 988 scaffolds and a scaffold N50 of 30.8 Mb (Supplementary Table 3), of which 446.2 Mb (94.9%) was anchored to 15 chromosomes (Supplementary Figure 2A and Table 1). This assembly of the “JGXP” genome was smaller than that of the previously reported cultivar “ZS4” genome (504 Mb) but larger than the cultivar “WF18” genome (440 Mb) (Table 1). We determined the homologous chromosomes among the three yellowhorn genomes based on shared synteny blocks (Supplementary Figure 3 and Supplementary Table 4). All the 15 chromosomes of the three genomes were in perfect 1:1 synteny (Figure 2 and Supplementary Figure 3). We also generated the complete plastid (Pt) genome (152,643 bp, Supplementary Figure 4) and mitochondrial (Mt) genome (389,005 bp, Supplementary Figure 5) from the sequence data.

We evaluated the quality of the “JGXP” assembly by several criteria. First, a 94.5% complete BUSCOs score suggests high-gene space completeness of the assembly, which was similar to “ZS4” (94.7%) but higher than “WF18” (84.6%) (Table 1). Second, the LTR Assembly Index (LAI) (Ou et al., 2018), a standard for evaluating the assembly using long terminal repeat retrotransposons (LTR-RTs), was 14.53 for our assembly, which classifies it into the “reference” category (Ou et al., 2018; Table 1). Finally, 99.39% of PacBio long reads, 91.43% of the transcriptome, and 97.66% of Illumina paired-end reads were mapped to the yellowhorn genome, respectively (Supplementary Table 5).

### Genome Annotation

A total of 22,049 high-confidence protein-coding genes were annotated, with 1,341 (93.1%) of complete core eukaryotic BUSCO genes covered (Supplementary Table 6). We identified 588 small ncRNA genes, 65 rRNA genes, and 708 tRNA genes (Supplementary Table 6). In addition, we identified a total of 16,386 pseudogenes, including 11,197 FRAGs (Fragment Pseudogenes), 4,120 DUPs (duplicated pseudogenes), and 1,069 PSSDs (retrotransposed pseudogenes) (Supplementary Table 6).

Nearly all (99.1%) of the protein-coding genes were functionally annotated by sequence and domain architecture similarity searches, with only 193 protein-encoding genes remaining completely uncharacterized (Supplementary Table 7). We identified 2,887 transcription factors (TFs), transcriptional regulators (TRs), and chromatin regulators (CRs) from 96 gene families in our “JGXP” assembly, including the major gene families of C2H2, CCHC (Zn), WD40-like, MYB, and PHD, respectively, which contained 457, 239, 236, 212, and 129 genes, respectively (Supplementary Table 8).

We identified 22,070 gene families among the three assemblies of yellowhorn, 50.9% (11,244) gene families were core gene families (Supplementary Figure 6). The genes of “JGXP” were clustered into 16,519 (74.8%) gene families, with 14,905 (67.6%) core genes, and only 1,046 (4.7%) private genes (Supplementary Figure 6). We found more dispensable genes in “JGXP” and “ZS4” than those in “WF18” (Supplementary Figure 6).

We identified 65.67% of the “JGXP” assembly as repetitive sequences (Supplementary Table 9). LTR-RTs were the most abundant transposable elements (TE), representing 29.64% of the “JGXP” assembly (Supplementary Table 9). Among the LTR-RTs, *Gypsy* (16.83%) and *Copia* (11.88%) were predominant (Supplementary Table 9). LINEs represent 4.06% of the genome, and most of them are LINE1, which represent 3.79% of the “JGXP” assembly (Supplementary Table 9). DNA transposons and the uncharacterized category “unknown” constituted 5.62% and 24.27% of the “JGXP” assembly, respectively (Supplementary Table 9). TEs were unevenly distributed along the chromosomes of the “JGXP” assembly, tending to accumulate in the regions of a low density of genes and high GC content for each chromosome (Figure 2). We re-annotated the repeat elements of the assemblies of “ZS4” and “WF18” using our annotation strategy. In general, the number and the length of each repeat element family were similar among the three assemblies of yellowhorn (Supplementary Figures 7A–C and Supplementary Table 9). However, five TE families, including LTR/Cassandra, LTR/DIRS, LINE/LINE1-Tx1, LINE/Penelope, and DNA/PiggyBac, were only present in our “JGXP” assembly, and 2,664 LTR/Ngaro elements were found in the “JGXP” assembly, while only 139 in the “WF18” assembly and absent in the “ZS4” assembly (Supplementary Figure 7C and Supplementary Table 9). To exclude the artificial processing, we further mapped PacBio long-reads from two accessions, “JGXP” and “ZS4,” to our “JGXP” assembly using minimap2 and checked whether the annotated TEs were supported under the mapping quality > 30. We found that almost all of the six TE families, including the LTR/Ngaro elements mentioned above, were verified by PacBio long-reads from “JGXP” and “ZS4” (Supplementary Table 10). It suggests these TEs are lost in the genome assemblies of “ZS4” and “WF18” during the genome assembly, or the TE annotation pipeline failed to recognize them.

### Genome Structural Variation

**FIGURE 3:**
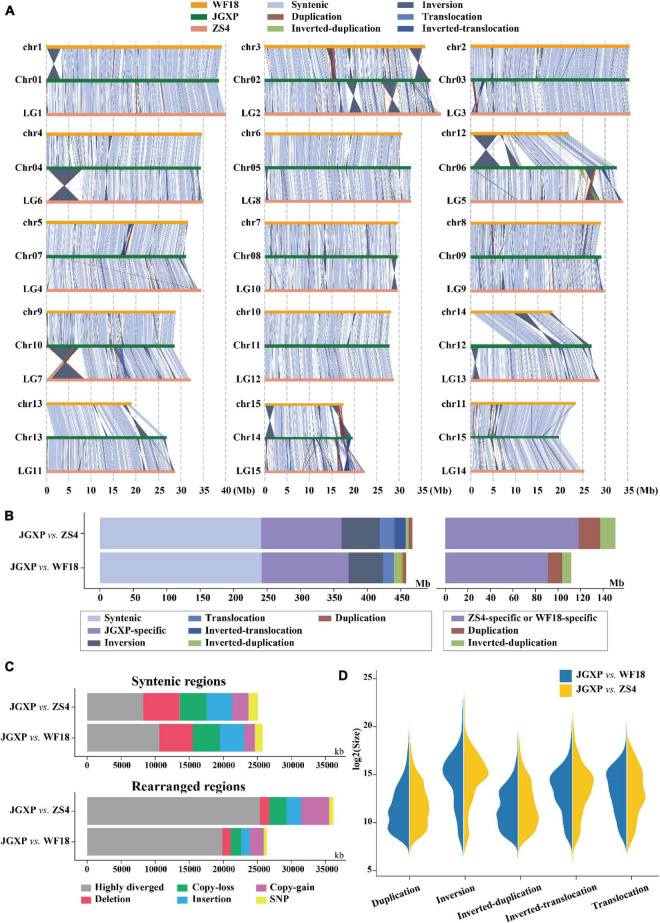
Comparative analysis among the three yellowhorn assemblies of “JGXP,” “ZS4,” and “WF18.” **(A)** Structural variations between the reference “JGXP” and the other two cultivars of yellowhorn genomes. The chromosome in the query genome has been reverse complemented if the majority of alignments between homologous chromosomes were inverted. **(B)** Barplot showing the total length of structural variations. **(C)** Barplot showing the sequence differences in the structural variations of syntenic (upper) and rearranged (lower) regions for “JGXP vs. ZS4” and “JGXP vs. WF18.” **(D)** Size distributions of different types of structural variations.

We compared the genomes of cultivars “JGXP,” “ZS4,” and “WF18” and identified structural variations (inversions, translocations, and duplications) and sequence differences (SNPs, indels) using “JGXP” as the reference. Genome comparison showed that the three genomes were in general syntenic (Figures 3A,C and Supplementary Figure 3). The syntenic regions encompassed 241.5 Mb (51.4%, 3,652 regions) for “JGXP *vs.* ZS4” and 242.2 Mb (51.5%, 3,027 regions) for “JGXP *vs.* WF18,” and the inversions were main structural arrangements, including 56.9 Mb (12.1%, 378 regions) for “JGXP *vs.* ZS4” and 51.4 Mb (10.9%, 426 regions) for “JGXP *vs*. WF18” (Figures 3B,C and Supplementary Table 11). However, we detected 119.9 Mb – 129.7 Mb (25.5–27.6%) JGXP-specific regions relative to the other two cultivars (Figure 3B and Supplementary Table 11).

Structural variations were found distributed unevenly among the chromosomes (Figure 3A). First, large fragments of structural variation were rare on chromosomes “Chr05” and “Chr11,” while they were abundant on “Chr02,” “Chr06,” and “Chr14” in “JGXP.” Second, large fragments of inversions were enriched in chromosome terminal ends. The size of inversion regions was larger than that of other structural variations, and the longest inversion was 8.1 Mb and found on “Chr04” (left end) of “JGXP” (Figures 3A,D).

### Centromere Identification

**FIGURE 4:**
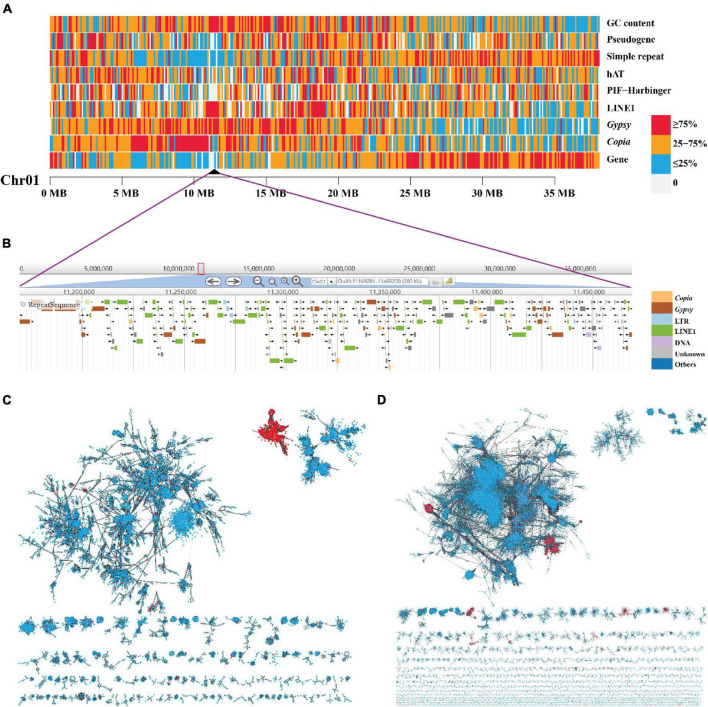
The high enrichment of LINE1 and *Gypsy* elements and their independent accumulation in the centromere of Chromosome 1. **(A)** A heat map view of protein-coding genes, TE (*Copia*, *Gypsy*, LINE1, PIF-Harbinger, and hAT), simple repeat, pseudogene, and GC content density in 100-Kb non-overlap sliding windows along chromosome 1 “Chr01.” The black triangle represents the predicted location of centromere. **(B)** The zoom in on the centromeric region. **(C,D)** The phylogenetic network of LINE1 and *Gypsy* elements, respectively. Each node in the network represents a single element. The links were defined as blast + alignment “bitscore” values (red, element in centromeric regions; blue, element in non-centromeric regions).

The most abundant tandem repeat is the centromeric sequences for most species (Melters et al., 2013). We do not find the tandem repeats detecting from PacBio long reads enriched preferentially in specific regions along the 15 chromosomes (Supplementary Figure 8 and Supplementary Data File 1). We used Centurion (Varoquaux et al., 2015) with Hi-C data as an alternative approach to predict the centromeres in yellowhorn genome. The centromere of each chromosome was predicted to a genomic point of one base pair (Table 2 and Supplementary Figure 2B). Based on the distribution of different TE families along the chromosomes, we noticed that LINE1 retrotransposons were enriched preferentially in narrow regions, and these regions highly matched the centromeres predicted by Centurion. These regions also contained a high density of *Gypsy* retrotransposons, *Copia* retrotransposons, and high GC content while a low density of genes (Figures 2, 4A,B and Supplementary Figures 2B, 9–22). A similar pattern is also found in the other two yellowhorn genome assemblies (“ZS4” and “WF18”) (Figure 2).

**TABLE 2:** A summary of centromere regions and chromosome types for each chromosome.

Chromosome	Predicted position (bp)	Start (bp)	End (bp)	Size (Mb)	Arm ratio (*r*)	Term
Chr01	11,336,700	10,700,001	12,100,000	1.4	2.36	sm
Chr02	18,486,900	17,600,001	19,200,000	1.6	1.02	m
Chr03	14,643,700	13,500,001	15,200,000	1.7	1.40	m
Chr04	13,374,100	12,500,001	13,900,000	1.4	1.55	m
Chr05	17,994,100	16,700,001	18,600,000	1.9	1.24	m
Chr06	20,311,600	19,900,001	20,900,000	1.0	1.70	m
Chr07	17,351,700	17,300,001	18,000,000	0.7	1.29	m
Chr08	13,037,700	11,800,001	14,000,000	2.2	1.28	m
Chr09	12,381,000	11,600,001	13,300,000	1.7	1.34	m
Chr10	16,802,400	16,200,001	17,700,000	1.5	1.48	m
Chr11	11,513,900	11,000,001	11,900,000	0.9	1.39	m
Chr12	20,460,700	19,500,001	22,000,000	2.5	3.22	st
Chr13	20,418,300	20,100,001	21,000,000	0.9	3.26	st
Chr14	11,055,000	10,500,001	11,600,000	1.1	1.30	m
Chr15	5,226,800	3,700,001	62,000,00	2.5	2.67	sm

*Arm ratio (r, long arm/short arm): m = metacentric, r from 1 to 1.7; sm = submetacentric, r from 1.7 to 3; st = subtelocentric, r from 3 to 7.*

Based on the density distribution of LINE1, we manually defined the borders of the putative centromeric region for each chromosome with a resolution of 100 kb (Table 2). The sizes of centromeres we identified ranged from 0.7 Mb to 2.5 Mb, summing up to 23 Mb (4.9% of the length of “JGXP” genome) (Table 2). We also classified the karyotype by calculating the arm ratio (*r*, long arm/short arm) for each chromosome as in the study of Levan et al. (1964). The 15 chromosomes were classified into 11 m (metacentric, *r* from 1 to 1.7) terms, 2 sm (submetacentric, *r* from 1.7 to 3) terms, and 2st (subtelocentric, *r* from 3 to 7) terms (Table 2). The karyotype of “JGXP” genome is thus 2n = 30 = 22m + 4sm + 4st.

We found a total of 3,312 (15.0%) LINE1, 6,592 (7.6%) *Gypsy*, 3,567 (5.6%) *Copia* retrotransposons, and 287 (1.3%) genes in the centromeric regions (Supplementary Table 12). Most of the genes in the centromeric regions are expressed (Supplementary Figure 23 and Supplementary Data File 2). The lengths of LINE1, *Gypsy*, and *Copia* elements in the centromeric regions were significantly longer than those in the non-centromeric regions (*p* < 0.0001, Wilcoxon test) (Supplementary Figure 24B and Supplementary Table 12). Additionally, we found 61 (34.9%) intact LINE1, 226 (8.1%) intact *Gypsy*, and 87 (2.3%) intact *Copia* retrotransposons in the centromeric regions (Supplementary Figure 24C and Supplementary Table 12). For the intact *Gypsy* retrotransposons in the centromeric regions, 117 (51.8%) were CRM (Supplementary Figure 24C).

The median insertion time of intact LINE1 and *Gypsy* elements in the centromeric regions was 0.67 MYA and 0.66 MYA, respectively; both were significantly younger than those in the non-centromeric regions (*p* < 0.01, Wilcoxon test) (Supplementary Figure 24A and Supplementary Table 13). However, the median insertion time of intact *Copia* elements in the centromeric regions was 1.36 MYA, which was significantly older than that in the non-centromeric regions (*p* < 0.05, Wilcoxon test) (Supplementary Figure 24A and Supplementary Table 13).

To examine whether the LINE1, *Gypsy*, and *Copia* in the centromeric regions were centromere-specific sequences, we constructed a sequence similarity-based phylogenetic network using these elements from the whole genome. The network showed that most of the LINE1 in the centromeric regions was clustered into one “module” (Figure 4C), and the *Gypsy* in the centromeric regions was clustered into two “modules” (Figure 4D), while the *Copia* in the centromeric regions did not distinguish from those in non-centromeric regions (Supplementary Figure 25). These indicate that the centromeres of yellowhorn are dominated by centromere-specific retrotransposons of LINE1 and *Gypsy*.

### Candidate Genes of Very-Long-Chain Fatty Acid Biosynthesis

In plants, VLCFA are important biological components of various lipids such as the triacylglycerols (TAGs), some sphingolipids and phospholipids, the cuticular waxes, and nervonic acid (Joubès et al., 2008; Ruan et al., 2017; Xu et al., 2019). VLCFA biosynthesis pathways involve four successive reactions and the first reaction, which catalyzes the condensation by the 3-ketoacyl-CoA synthase (KCS) or elongation-defective-like (ELO-like) enzyme of a long chain acyl-CoA with a malonyl-CoA, is the synthesis rate-limiting step (Haslam and Kunst, 2013). Twenty-one KCS genes were identified in *Arabidopsis thaliana* and classified into eight phylogenetic subclasses: α, β, γ, δ, ζ, ε, η, and θ (Costaglioli et al., 2005; Joubès et al., 2008).

**FIGURE 5:**
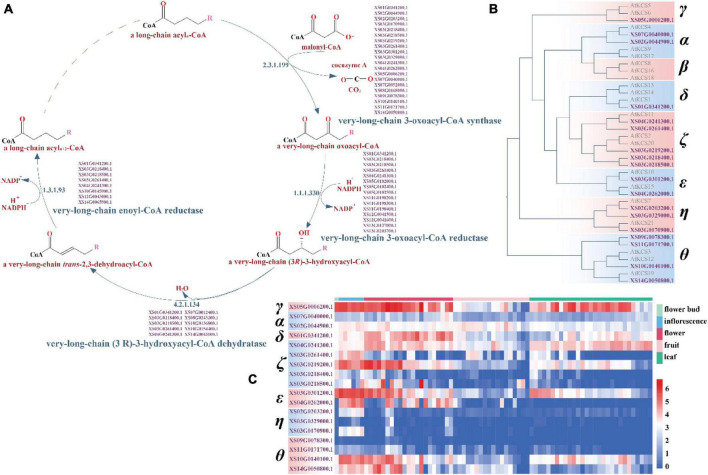
The very-long-chain fatty acid biosynthesis pathway and the classification and expression profile of the candidate KCS genes. **(A)** The candidate genes-encoding key enzymes in the four reactions of the very long-chain fatty acid synthesis pathway in yellowhorn genome. **(B)** Phylogenetic tree of the candidate KCS genes in *Arabidopsis* and yellowhorn. **(C)** The candidate KCS genes expression profile. The expression values were normalized by ln (TPM + 1).

Sequence similarity-based functional annotation identified 38 candidate genes in VLCFA biosynthesis in yellowhorn, of which 18 were KCS genes and two ELO-like genes (Figures 5A,B and Supplementary Data File 3). Phylogenetic analysis divided the 18 KCS genes into seven subclasses with the absence of the β subclass: 2 α genes, 1 γ gene, 1 δ gene, 5 ζ genes, 2 ε genes, 3 η genes, and 4 θ genes (Figure 5B). Overall, the domain structure is highly conservation among the subclasses of KCS gene, with 10 of the KCS genes displaying no intron structure (Supplementary Figure 26).

In *Arabidopsis*, the six KCS genes in the α (*KCS4*, *KCS9*, and *KCS17*) and β (*KCS8*, *KCS16*, and *KCS18*) subclasses are closely related to the seed-specific condensing enzyme that play a role in seed oil production, whereas the other 15 genes have been implicated in the synthesis of wax components (Costaglioli et al., 2005; Joubès et al., 2008). The two KCS candidate genes of the α subclass in yellowhorn, *XS02G0044900.1* and *XS07G0040000.1*, were most similar to *KCS4*, indicating that they may be involved in catalyzing a condensing reaction of VLCFA biosynthesis (Figure 5B). These two genes in yellowhorn showed differential patterns of expression in flower bud, inflorescence, flower, fruit, and leaf tissues (Figure 5C).

### Long Terminal Repeat Retrotransposons Evolution

Long terminal repeat retrotransposons are the highest portion of TEs, representing ∼30% of yellowhorn genome (Supplementary Figure 7A-B and Supplementary Table 9). To investigate the mode and evolution of the expansion of LTR-RTs in yellowhorn, we identified the intact LTR-RTs, solo-LTRs (the LTRs without *Gag-Pol*), and truncated LTR-RTs in the “JGXP” genome and 16 other plant genomes (Supplementary Table 14). A total of 6,749 intact LTR-RTs (*I*) were identified in yellowhorn (Supplementary Table 15), much more than in the other genomes, indicating that intact LTR-RTs (*I*) are maintained at a higher frequency in yellowhorn (Supplementary Figure 26A and Supplementary Table 15). To estimate LTR-RT birth and removal rates, we compared the numbers of solo-LTRs (*S*) and truncated LTR-RTs (*T*). The truncated LTR-RTs (33,692) were far more prevalent than solo-LTRs (10,771) in the yellowhorn genome (Supplementary Table 15). The *I* + *S* + *T* values of yellowhorn were moderate compared with the other 16 plants (Supplementary Figure 27C and Supplementary Table 15), which can represent the birth rate of LTR-RTs (Lyu et al., 2018). Since the fragmental scaffolds of the genome affect the identification of the three classes of LTRs, we corrected the counting by filtering out short scaffolds and then calculated the ratios of filtered *S:I*, *T:I*, and *(S* + *T):I*, which were 1.53, 4.98, and 5.50, respectively. These ratios are relatively low compared with the other 16 plant genomes (Supplementary Table 15). We further analyzed the trends of *S*:*I* among clusters of LTR-RT sequences by their similarity. Cluster-level *S*:*I* values reflect the removal rate for a specific family. We considered groups with filtered *S*:*I* > 3 to have a high death rate as defined by a previous study (Lyu et al., 2018). We found 18.66% of the sequence families have high removal rates, which are a low proportion compared with the other 16 plant genomes (Supplementary Figure 27E and Supplementary Table 15). Thus, the high proportion of LTR-RTs in yellowhorn is maintained by a moderate birth rate and a low removal rate.

### Phylogenetic Inference and Gene Family Evolution

We constructed a phylogenetic tree using a concatenated sequence alignment of 201 single-copy orthologous genes among the yellowhorn genome and 16 other plant genomes. In the phylogenetic tree, yellowhorn and *Dimocarpus longan* were clustered into a group of the Sapindaceae family with an estimated divergence time of ∼53 MYA (Supplementary Figure 28A). Our analysis supports the grouping of *Populus trichocarpa* with malvids rather than fabids and the grouping of myrtales as a sister taxon to the eurosids rather than a taxon in malvids, in agreement with recently published whole-genome studies (Myburg et al., 2014; Yang et al., 2017).

Expanded gene families (EGF), regardless of duplication type, provide the raw material for adaptation and trait evolution. We compared 569,169 protein-coding genes from the 17 plant species, yielding a total of 33,631 gene families that comprised 449,645 genes. A total of 181,959 genes belonging to 5,873 gene families were shared among all 17 plant genomes. We found that 282 gene families comprising 830 genes were unique for yellowhorn genome. A total of 964 orthologous groups with 3,107 genes were EGF in the yellowhorn lineage since divergence from *D. longan* (Supplementary Figure 28A). EGF genes were significantly enriched (*FDR* < 0.001) in a number of gene ontologies (GO) of the flavonoid metabolic process (GO:0009812) and the flavonoid biosynthetic process (GO:0009813), and more specifically in quercetin 3-O-glucosyltransferase activity (GO:0080043), UDP-glucosyltransferase activity (GO:0035251), and flavonoid glucuronidation (GO:0052696) (Supplementary Figure 29).

### The Gamma Hexaploidy Event

The collinear blocks within yellowhorn provided evidence for the gamma (γ) hexaploidy event that remained visible in chromosomes 2, 7, and 8 (Supplementary Figure 28B). The distribution of *Ks* (synonymous substitution rate) in yellowhorn and *Vitis vinifera* (grape) was similar, both showed the peaks of *Ks* at around 1.4–1.6 (117 MYA-132 MYA), which further support that yellowhorn and grape shared the γ event (Supplementary Figure 28C). The dotplot of collinear blocks within the yellowhorn genome and the 1:1 collinear pattern between yellowhorn and grape indicated that the genome has not undergone a whole-genome duplication (WGD) event since its divergence from grape (Supplementary Figure 28B, 27C).

## Discussion

This study presents a high-quality chromosome-scale genome assembly and extensive comparative analyses on genome diversity and centromere evolution for a valuable oil-producing tree species yellowhorn. Our investigation provided insights into centromere structure, sequence composition, and evolutionary dynamics that contribute to our understanding of centromere biology.

By providing an additional reference genome for yellowhorn, we were able to compare genome variations among three cultivars. The three cultivar genomes are largely syntenic (∼51%), but genome-specific regions were also substantial, making up to 26–27% of the genome between cultivar comparisons. Structural rearrangements were detected among the cultivars with inversions, accounting for 11–12%. These suggest that there is substantial genomic variation in the species, and that one specific cultivar was insufficient to capture the entire genome property of yellowhorn. Large-scale re-sequencing study could provide a better understanding about the degree of diversity in different categories/families of sequences, and thus guide effective breeding efforts.

Centromeric tandem repeats are the dominant sequences of centromeres in most species, while, in some species, retrotransposons of *Gypsy* elements are also reported (Csink and Henikoff, 1998; Neumann et al., 2011). Centromeres are one of the difficult and mysterious parts of many high-quality genomes. They are comprised of highly repetitive elements and can vary dramatically even among closely related species (Yang et al., 2021). There were no readily apparent conserved characteristics for the candidate centromere tandem repeats from ∼300 animals and plants (Melters et al., 2013). Interestingly, the typical tandem centromeric repeats were not found in yellowhorn; instead, we discovered centromere-specific retrotransposons of LINE1 and *Gypsy*, which are dominant centromeres. The size of centromeric regions varies among chromosomes, ranging from 0.4 Mb to 1.4 Mb in *A. thaliana* and from 0.065 Mb to 2 Mb in *O. sativa* (Round et al., 1997; Copenhaver et al., 1999; Cheng et al., 2002). In yellowhorn, centromere size on each chromosome ranged from 0.7 Mb to 2.5 Mb; the total size of centromeres was 23 Mb, of which 4.2 Mb was LINE1 elements and 6.2 Mb *Gypsy* elements. To date, centromere-specific LINE (named *Nanica*) elements are found only in *M. acuminata*, but the origin and evolutionary dynamic of such centromeric LINEs are unclear (D’Hont et al., 2012; Čížková et al., 2013; Belser et al., 2021). Our analysis suggests that the insertion time of centromeric LINE1 and *Gypsy* elements (0.67 MYA and.66 MYA) was significantly younger than those in non-centromeric regions. This indicates that the centromeres are going through rapid evolution in yellowhorn. The previous study showed that some centromeres adopt new positions over evolutionary time subsequent to a speciation event by comparing the closely related species human and macaque (McKinley and Cheeseman, 2016). The recent enrichment of LINE1 and *Gypsy* elements and the lack of typical tandem centromeric repeats indicates that we identified a case of recently evolved centromeres in yellowhorn. Our finding of the enrichment of centromere-specific retrotransposons deserves further verification of centromeric localization by analyses such as the ChIP-seq with an antibody against the fast-evolving CENH3 (Centromere Specific Histone 3) protein.

The seed oil of yellowhorn contains 1.5–3.% nervonic acid (Ruan et al., 2017), which has great potential for production nervonic acid. We identified the biosynthetic pathway of VLCFA in yellowhorn and revealed associated gene expression patterns. KCS enzymes catalyze the synthesis of several VLCFA, including nervonic acid (Millar and Kunst, 1997; Guo et al., 2009; González-Mellado et al., 2019). We assayed the expression of the yellowhorn KCS genes by comparing different tissues at various developmental stages using RNA-Seq. Most KCS genes were highly expressed in flowers and inflorescences, two genes were moderately expressed in leaves, while almost all genes were lowly expressed in fruits. This result directs us to a hypothesis that the VLCFA in the seeds of yellowhorn may be synthesized and accumulated during flowering, or it is synthesized in leaves and then transported to seeds for storage. Our results are important for further investigation and manipulation of nervonic acid synthesis in plants.

In conclusion, the characterization of the reference genome sequence of yellowhorn presented here provides a key resource for further development of hypotheses in plant centromere evolution and functioning, and advancement of plant biotechnology in yellowhorn improvement and breeding, such as molecular marker-assisted selection and genome editing.

## Materials and Methods

### Plant Material and Sequencing

The sequenced individual, “Jinguanxiapei” (abbreviated with “JGXP”), was collected from a natural yellowhorn stand in Chengde, Hebei Province, China. DNA was extracted from young leaves of this variety in the early spring using a cetyl trimethyl ammonium bromide (CTAB)-based method (Doyle and Doyle, 1987).

Three approaches were employed in DNA sequencing. First, 2 × 150 pair-end libraries were sequenced on the Illumina HiSeq X Ten platform. Second, SMART libraries were constructed using PacBio^®^ SMRTbell™ Template Prep Kit 4.0 V2, following the PacBio 20-Kb protocol^1^ and sequenced on PacBio RS II and PacBio SEQUEL. Third, a Hi-C library was prepared following a published protocol (Wang et al., 2015) and sequenced on Illumina HiSeq 2500.

### Estimating Genome Size, Heterozygosity, and Repeat Content

The 17-bp *K*-mers were counted using Jellyfish v1.1.11 (Marcais and Kingsford, 2011) with default parameters using corrected PacBio reads. The genome size, the level of heterozygosity, and repeat content were estimated using gce v1.0.0 (Liu et al., 2013) using PacBio reads. We also estimated the heterozygosity by mapping Illumina paired-end reads using bowtie 2 (Langmead and Salzberg, 2012) to the assembled genome and calling the heterozygous variant locus using samtools/bcftools pipeline (Li et al., 2009).

### *De novo* Genome Assembly

The *de novo* assembly was prepared as follows in a progressive manner. The primary version v0.1 was assembled by SMART *de novo* v1.0.0 (Liu et al., 2021) after correction with Canu v1.6. The contigs of assembly v0.1 were polished using arrow v2.2.1 with PacBio long reads, which were further used for scaffolding using SSPACE-LongReadv1.1 (Boetzer and Pirovano, 2014) and SSPACE-standard v3.0 (Boetzer et al., 2011) and using GapCloser v1.12 (Luo et al., 2012) with Illumina paired-end reads. After one round of polishing by arrow v2.2.1 and three rounds of polishing by pilon v1.22 (Walker et al., 2014), we generated assembly v1.2. We mapped the Hi-C reads to the assembly v1.2 using Juicer v1.5.6 (Durand et al., 2016) to correct the mis-joined scaffolds using the 3D-DNA pipeline (version 170123) (Dudchenko et al., 2017) with Hi-C reads. Afterward, we then generated assembly v2.2 after three rounds of polishing using arrow v2.2.1 and three rounds of polishing using pilon v1.22.

We failed to assemble the complete genome of chloroplast (Pt) and mitochondrial (Mt) in the v2.2 assembly. The PacBio long reads of Pt and Mt were enriched by sequence similarity search against 11 Pt genomes of Sapindaceae and 24 Mt genomes of Malvidae available in the NCBI database^2^, and then the two genomes were *de novo* assembly using Canu v1.6.

After merging the assembly v2.2, Pt genome, and Mt genome, we removed redundancy sequence with Redundans v0.13c (Pryszcz and Gabaldón, 2016), and then generated the final assembly of the “JGXP” genome.

### Assessment of Genome Completeness

Genome completeness was assessed using the plant data set of BUSCO (Benchmarking Universal Single-Copy Orthologs) (Simao et al., 2015), LTR Assembly Index (LAI) (Ou et al., 2018), and the mapping rate, including PacBio long reads, Illumina paired-end reads, and the transcriptome assembled in the current study.

### Transcriptome Assembly

To construct a comprehensive yellowhorn transcriptome, three methods, including *de novo* and reference genome-guided assembly using Trinity v2.0.6 (Grabherr et al., 2011), reference genome-guided using StringTie v1.3.5 (Pertea et al., 2015) and HiSat2 v2.1.0 (Kim et al., 2015) and were performed using 75 Illumina paired-end samples in the current study (Supplementary Table 16). These three sets of transcriptomes were merged and further refined using CD-HIT v4.6 (Fu et al., 2012) with 95% identity and 95% coverage.

### Gene Prediction and Functional Annotation

Three approaches, including transcript-based prediction, protein homology-based prediction, and *ab initio* prediction, were employed to predict the protein-coding genes using repeat-masked version genome. Protein sequences of *Arabidopsis thaliana* (Swarbreck et al., 2007), *Olea europaea* (Fernando et al., 2016), *Dimocarpus longan* (Lin et al., 2017), and *Citrus grandis* (Wang X. et al., 2017), were merged and further refined using CD-HIT v4.6 (Fu et al., 2012) with 95% identity and 95% coverage. The transcriptome and protein sequences were aligned with the repeat-masked genome using BLAST, respectively, and further optimized the alignment using Exonerate v2.4.0 (Slater and Birney, 2005). Single-copy genes identified by BUSCO (Simao et al., 2015) were trained and further used for *ab initio* gene prediction using AUGUSTUS v3.2.3 (Stanke et al., 2008; Keller et al., 2011). The transcripts, proteins, and *ab initio* predictions were combined as evidence hints for the input of the MAKER v2.31.9 (Cantarel et al., 2008) annotation pipeline for final gene model prediction. The completeness of gene annotation was assessed using BUSCO.

The predicted protein-coding genes were functionally annotated using two approaches: (1) the sequence similarity searching method by five functional databases: the NR (NCBI’s non-redundant protein) database, the Swiss-Prot protein database, the TrEMBL database, the Pfam database, and the eggNOG database (Jensen et al., 2007), and (2) the domain architecture similarity searching method by InterProScan v5.27-66.0 (Jones et al., 2014). In addition, transcription factors, transcriptional regulators, and chromatin regulators were annotated using PlanTFcat (Dai et al., 2013).

Pseudogenes were identified using Pseudopipe (Zhang et al., 2006) with default parameters. The tRNA genes and rRNA genes were predicted using tRNAScan-SE v1.3.1 (Lowe and Eddy, 1997) and RNAMMER v1.2 (Lagesen et al., 2007), respectively. The small non-coding RNA genes were subjected to similarity searches against the Rfam (11) database using rfam_scan.pl (Burge et al., 2012). We used GeSeq (Tillich et al., 2017) to predict the protein-coding genes, rRNA genes, and tRNA genes of Pt genome and Mt genome, respectively.

### Expression Quantification

Before mapping the reads to the genome, all reads were filtered for adapter contamination, ambiguous residues (N’s), low-quality regions lower than 30, and reads shorter than 60 bp using cutadapt (Martin, 2011). The clean reads were mapped to the genome using HiSat2 v2.1.0 (Kim et al., 2015) with the parameter “-k 1.” We calculated the TPM values of genes using StringTie v1.3.5 (Pertea et al., 2015).

### Genome Comparison

We performed the pairwise alignment among the yellowhorn genome in the current study and the two previously published assembled genomes “ZS4” (Bioproject accession: PRJNA483857) (Bi et al., 2019) and “WF18” (Bioproject accession: PRJNA496350) (Liang et al., 2019) using minimap2 (Li, 2018). The syntenic regions, structural rearrangements (inversions, translocations, and duplications), and the sequence differences (SNPs, indels, and so on) of the pairwise comparison for the three genomes were identified using SyRI v1.3 (Goel et al., 2019). The pairwise homolog chromosomes among the three genomes were determined by the shared synteny blocks based on the dotplots of the pairwise alignments.

### Gene Family Clustering Among Three Cultivars of Yellowhorn

The core and the dispensable gene sets were summarized based on gene family clustering with protein sequences of the three cultivars using OrthoFinder v2.5.2 (Emms and Kelly, 2019) with default parameters. The BLASTP with *E*-value of 1*E*-10 implemented in diamond v0.9.9.110 (Buchfink et al., 2021) was performed for homologous searching. The gene families present in all three and two cultivars were defined as core gene families and dispensable gene families, respectively. Those that only existed in one accession were defined as private gene families.

### Centromere Identification

As tandem repeats are typical components of centromeric chromosome regions, we first followed Melters’s approach (Melters et al., 2013) to identify the centromeric regions using PacBio long reads. After masking the low complexity of the long reads using DUST implemented in MEME suite v4.11.3 (Bailey et al., 2009), tandem repeats were detected using TRF v4.09 (Benson, 1999). Tandem repeats > 90% identity were clustered, and the repeats in the top clusters are presumed to be the candidate centromeric repeat. However, we do not find centromeric tandem repeats in the yellowhorn genome (Supplementary Figure 8 and Supplementary Data File 1).

Centromeres are tethered to the spindle pole body, leading to centromere clustering (Feng et al., 2014; Mizuguchi et al., 2014). The spatial proximity reflected by the Hi-C interaction intensity decreased along with the increasing of physical distance between two loci (Lieberman-Aiden et al., 2009). Thus, we also performed Centurion (Varoquaux et al., 2015) to identify the location of centromeres using a genome-wide Hi-C contact map. Centurion was performed to call centromere locations in the yellowhorn genome using the Hi-C sequencing data generated in the current study. The centromere location for each chromosome predicted by Centurion was presented as a genomic point of one base pair.

We noted that LINE1 retrotransposons were accumulated preferentially in narrow regions (Figure 2), and these regions highly match the centromeres predicted by Centurion (Supplementary Figure 2B). The density of *Gypsy* retrotransposons and GC content was high, while the density of genes was low in these regions (Figures 2, 4A,B and Supplementary Figures 9–22). Based on these, we manually defined the start and the end of the centromeric regions with a resolution of 100 kb according. We calculated the arm ratio for each chromosome, long arm/short arm, to classify the karyotype according to previous study (Levan et al., 1964).

### Phylogenetic Network of Transposable Elements

To generate weighted links, the sequences of LINE1, *Copia*, and *Gypsy* elements were pairwise aligned using BLASTN v2.2.31 (“-strand plus -dust no -max_target_seqs 4000”). The link weights were defined as alignment “bitscores.” We did not set a threshold to remove links to avoid disconnecting whole modules of ancient sequences from the network (Levy et al., 2017). For efficiency and improved perception, we disconnected the weakest links of each node for *Gypsy* and *Copia* network, and retained the top strongest 3% and 10% of strongest links, respectively. We displayed all of the links for LINE1 network. The network was visualized with Cytoscape (Shannon et al., 2003).

### Insertion Dating of Long Interspersed Nuclear Elements 1 and Long Terminal Repeat Retrotransposons

The LINE1 retrotransposons with its best BLAST hit (Yang and Bennetzen, 2009) and 5’-LTRs and 3’-LTRs of the same LTR-RTs were aligned using MAFFT v7.221 (Katoh and Standley, 2013), and the corresponding divergence *K* was estimated using the Kimura Two-Parameter model (Kimura, 1980). The insertion time was calculated by the formula: *T* = *K*/(2 × *r*), where *r* refers to a substitution rate of 1.3 × 10^–8^ per site per year (Ma et al., 2004).

### Intact Long Interspersed Nuclear Elements 1 Elements

We performed getorf (“-find 1 -minsize 800”) implemented in EMBOSS v6.5.7.0 (Rice et al., 2000) to identify the ORFs (open reading frames) of the LINE1 elements extended 1-Kb flanking regions. The identified ORFs were annotated using hmmscan v3.2 (Mistry et al., 2013) with Pfam31 (Finn et al., 2009). The intact LINE1 elements were screened as the descriptions of previous study (Ivancevic et al., 2016).

### Candidate Genes of the Very Long-Chain Fatty Acids Biosynthesis Pathway

Protein-coding genes were annotated with enzyme function classes using E2P2 (Ensemble Enzyme Prediction Pipeline) v3.1 (Chae et al., 2014) and then assigned to PLANTCYC v13.0^3^ using Pathway Tools v22.5 (Karp et al., 2015) for the prediction of genes involving in the VLCFA biosynthesis pathway. The KCS genes were annotated using CDD (conserved domain database) (Lu et al., 2020) and SMART (simple modular architecture research tool) (Letunic et al., 2020). To construct the maximum likelihood tree of KSC genes, including in yellowhorn and *A. thaliana*, IQ-TREEv1.6.7 (Nguyen et al., 2015) was performed with the optimal amino acid substitution model of LG + I + G4 with 1,000 ultrafast bootstrapping. The visualization was displayed using TB tools v1.068 (Chen et al., 2020).

### Repetitive Element Identification and Long Terminal Repeat Retrotransposons Evolution

The *de novo* repeat identification approach was employed to annotate the repeat elements. First, RepeatModeler v1.0.10 (Smit and Hubley, 2008) was performed to train a repeat database by BLAST approach, and then RepeatMasker v4.07 (Smit et al., 2013) was used to annotate the repeat elements based on the database above.

To accurately identify the LTR-RTs, LTRharvest v1.5.10 (Ellinghaus et al., 2008) and LTRdigest v1.5.10 (Steinbiss et al., 2009) were used to *de novo* identify the candidate intact LTR-RTs with a pair of flanking LTRs ranged from 100 bp to 3,000 bp with similarity > 80%. The domain-based annotation method implemented in Profrep^4^ was performed to annotate the internal sequences of candidate LTR-RTs using the REXdb v3.0 database (Neumann et al., 2019). An LTR-RT with complete *Gag-Pol* protein sequence was retained as an intact LTR-RT (*I*). If one side of the flanking sequences covered at least 50% of any *Gag-Pol* sequences with *E*-value < 1*E*-8 and identity > 30%, the corresponding LTR homologies were classified as truncated LTR-RTs (*T*). The LTRs without *Gag-Pol* were considered as solo-LTRs (*S*). SiLiX v1.2.9 (Miele et al., 2011) was performed to cluster the LTRs with the coverage of 70% and the identity of 60%.

### Phylogenetic and Gene Family Analysis

OrthoMCL v2.0.9 (Li et al., 2003) was used to identify gene family with the protein-coding genes of yellowhorn and the other 16 plants species (Supplementary Table 14). A total of 201 single-copy gene families were identified and used for phylogenetic tree reconstruction. Each single-copy gene family was aligned using MUSCLE v3.8.425 (Edgar, 2004) with default parameters. The alignments of each gene family were concatenated into a single alignment. This alignment was trimmed using trimAl v1.4.rev15 (Capella-Gutierrez et al., 2009). The trimmed alignment was used for the maximum likelihood phylogenetic tree reconstruction using IQ-TREE v1.6.7 (Nguyen et al., 2015), with the best-fit model JTT + F + R5 selected by ModelFinder (Kalyaanamoorthy et al., 2017) and with the 1,000 replications of ultrafast bootstrap and Shimodaira-Hasegawa-like approximate likelihood-ratio (SH-aLRT) test.

The MCMCTree in PAML v4.9h (Yang, 2007) was run to estimate the divergence time. The divergence time between *O. sativa* and Pentapetalae (other 16 species), representing the monocot-dicot divergence, was fixed at 130 to 135 MYA in the present study (Magallón et al., 2015). The divergence of Rosids from other Pentapetalae species was at least 99.6 MYA (Basinger and Dilcher, 1984; Magallón et al., 2015), and the divergence of *C. grandis* from other Sapindales species was at least 65.5 MYA (Magallón et al., 2015).

Expansion and contraction of the families were determined using CAFE v4.2 (Han et al., 2013) with default parameters. Enrichment of gene ontology (GO) terms was summarized using clusterProfiler v3.8.1 (Yu et al., 2012). We controlled the false discovery rate (*FDR*) of the *P* values using Benjamini-Hochberg procedure (Benjamini and Hochberg, 1995).

### Analysis of Genome Duplication Event

Syntenic blocks containing at least five genes were identified using MCscanX (Wang et al., 2012) with default parameters. KaKsCalculator v2.0 (Wang et al., 2010) was used to calculate *Ks* with the YN model. Only the gene pairs with *Ks* ≤ 3 were remained for the downstream analysis.

### Visualization

Visualization of the predicted distribution of yellowhorn based on sampled records and current climate data (Wang Q. et al., 2017) was conducted in ArcGIS v9.2. The screens of zoom in on the centromeric regions were generated using JBrowse implemented in PlantGenIE (Skinner et al., 2009; Sundell et al., 2015).

## Data Availability Statement

The raw sequence data have been deposited in the Short Read Archive under NCBI BioProject ID PRJNA694500. The Whole Genome Shotgun project has been deposited at DDBJ/ENA/GenBank under the accession JAFEMO000000000. The version described in this paper is version JAFEMO010000000. Genome assembly, repeat and gene annotation, transcriptome, and gene expression profiles could be downloaded and explored online under URL: https://yellowhorn.plantgenie.org/.

## Author Contributions

J-FM, HL, and WG conceived and designed the study. HL, X-MY, Xin-ruiW, D-XZ, QZ, T-LS, K-HJ, X-CT, S-SZ, R-GZ, Q-ZY, QW, QX, CM, and EV prepared the materials and conducted the experiments. HL, X-MY, and J-FM wrote the manuscript. Xiao-ruW, J-FM, WZ, NS, IP, and YE-K were involved in structuring and polishing the manuscript. All authors contributed to the article and approved the submitted version.

## Conflict of Interest

The remaining authors declare that the research was conducted in the absence of any commercial or financial relationships that could be construed as a potential conflict of interest.

## Publisher’s Note

All claims expressed in this article are solely those of the authors and do not necessarily represent those of their affiliated organizations, or those of the publisher, the editors and the reviewers. Any product that may be evaluated in this article, or claim that may be made by its manufacturer, is not guaranteed or endorsed by the publisher.

## Footnotes

1
https://www.pacb.com/

2
https://www.ncbi.nlm.nih.gov/

3
https://pmn.plantcyc.org/organism-summary?object=PLANT

4
https://bitbucket.org/nina_h/profrep/wiki/Home
