# Supplementary material for: Centromere-Specific Retrotransposons and Very-Long-Chain Fatty Acid Biosynthesis in the Genome of Yellowhorn (Xanthoceras sorbifolium, Sapindaceae), an Oil-Producing Tree With Significant Drought Resistance
Source: Front Plant Sci. 2021 Nov 22;12:766389. doi: 10.3389/fpls.2021.766389 (PMC8647845; doi:10.3389/fpls.2021.766389)
Supplement: Supplementary file 1 [file Data_Sheet_1.zip › Supplementary Figures and Tables.docx]

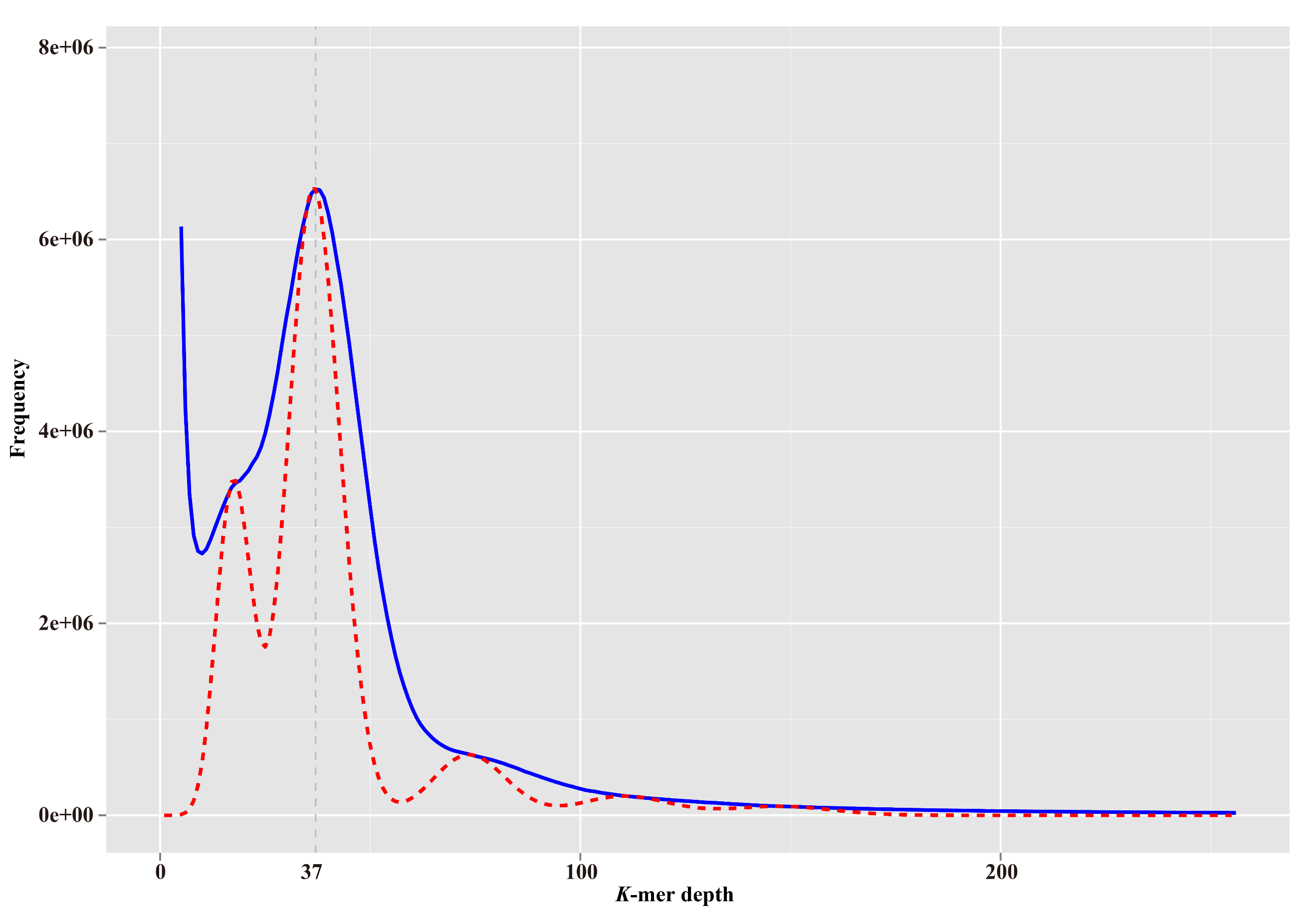


**Supplementary Figure 1. *K*-mer frequency distribution estimated from 40x PacBio sequences after filtering and correction at a size of 17 bp.** Blue solid line for observed *K*-mer frequency distribution, red dash line for fitted model of *K*-mer frequency distribution.


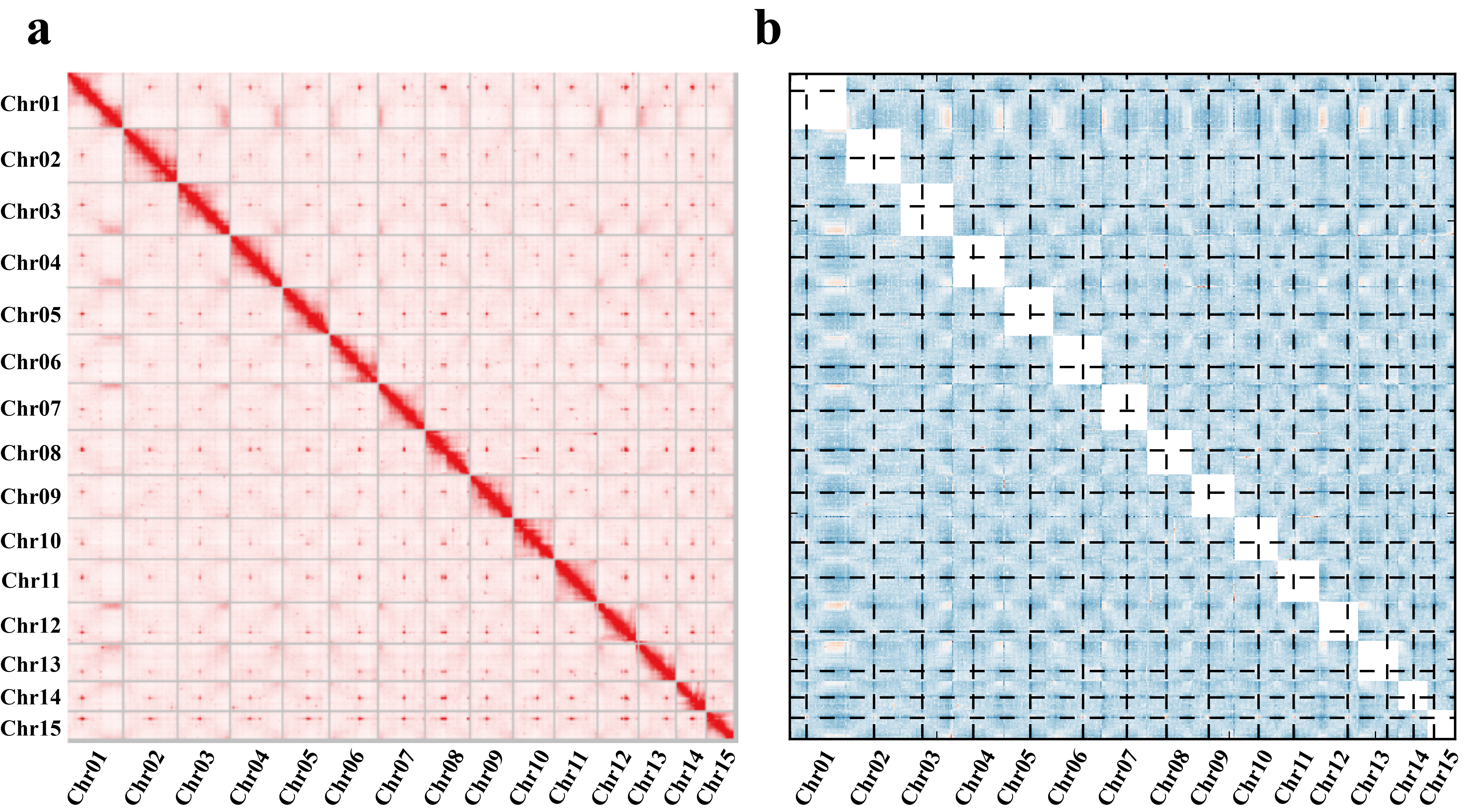


**Supplementary Figure 2. Heatmap showing the contact counts of H-C data.** (a) Contact map of Hi-C links among chromosomes. Darker red color indicates higher Hi-C contact density in the plot. (b) Heatmap of the normalized *trans* contact counts with *de novo* centromeres calls. White squares represent chromosomes and the crosses of dash lines mark the predicted centromeres. The contact counts were smoothed with a Gaussian filter for visualization purposes.


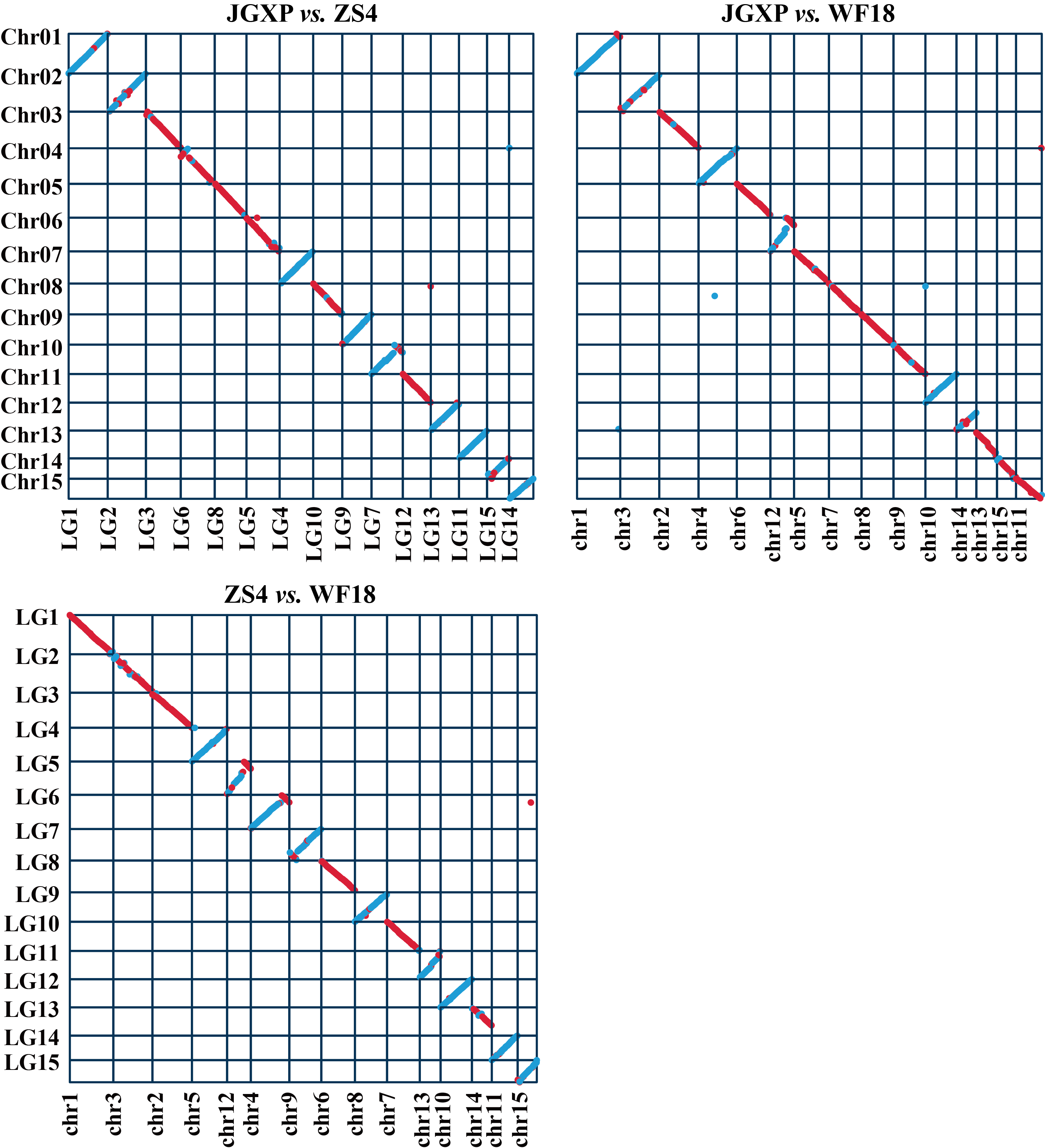


**Supplementary Figure 3. Dot plots showing the genome comparison among the three yellowhorn assemblies of “JGXP”, “ZS4”, and “WF18”.** Dots represent the homology sequences.


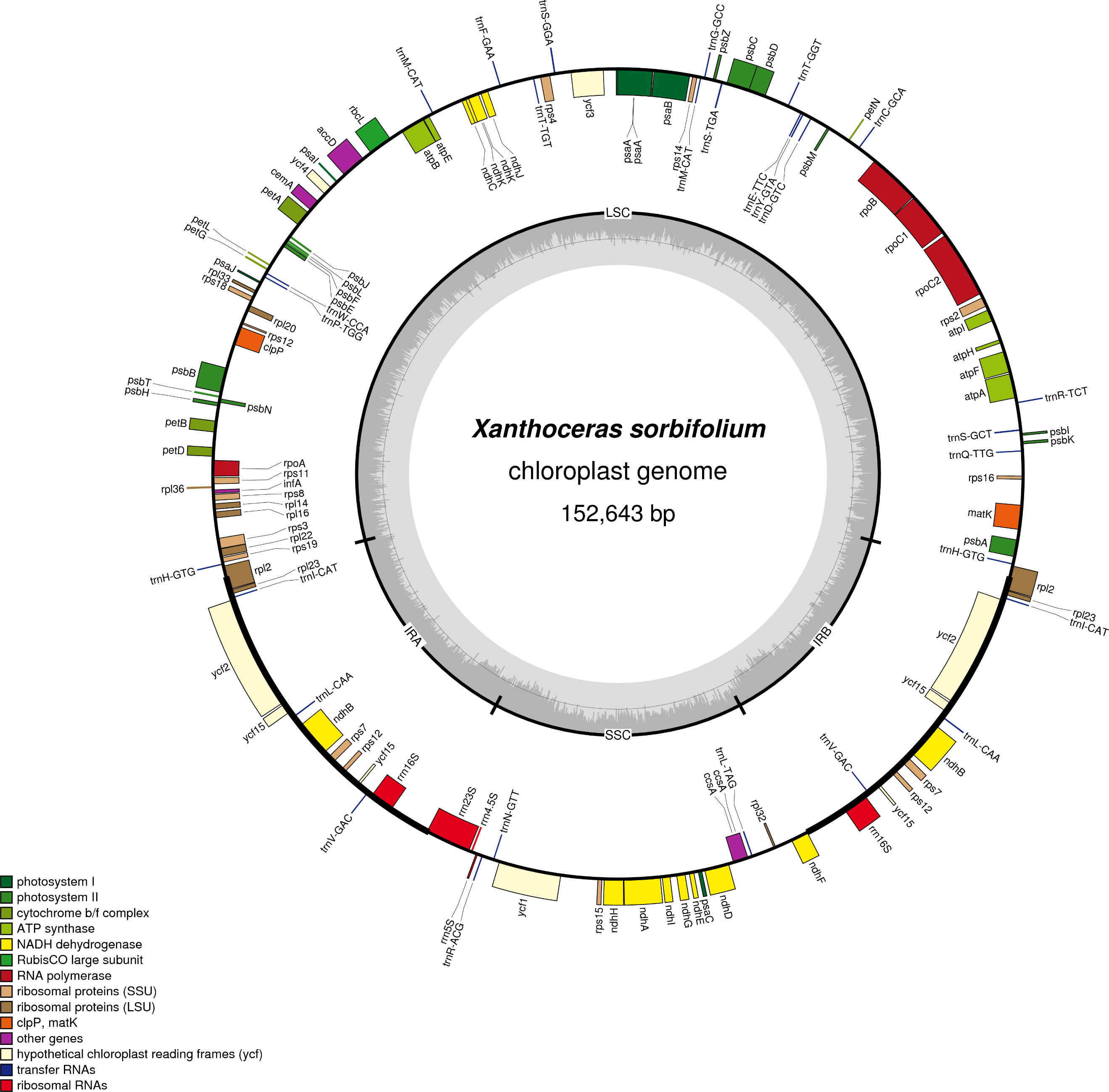


**Supplementary Figure 4. The chloroplast genome of yellowhorn.** Genes (exons were closed boxes) shown on the outside of the circle were transcribed clockwise, whereas those on the inside were transcribed counter-clockwise. Genes from the same protein complex were colored the same, introns were indicated in white boxes.


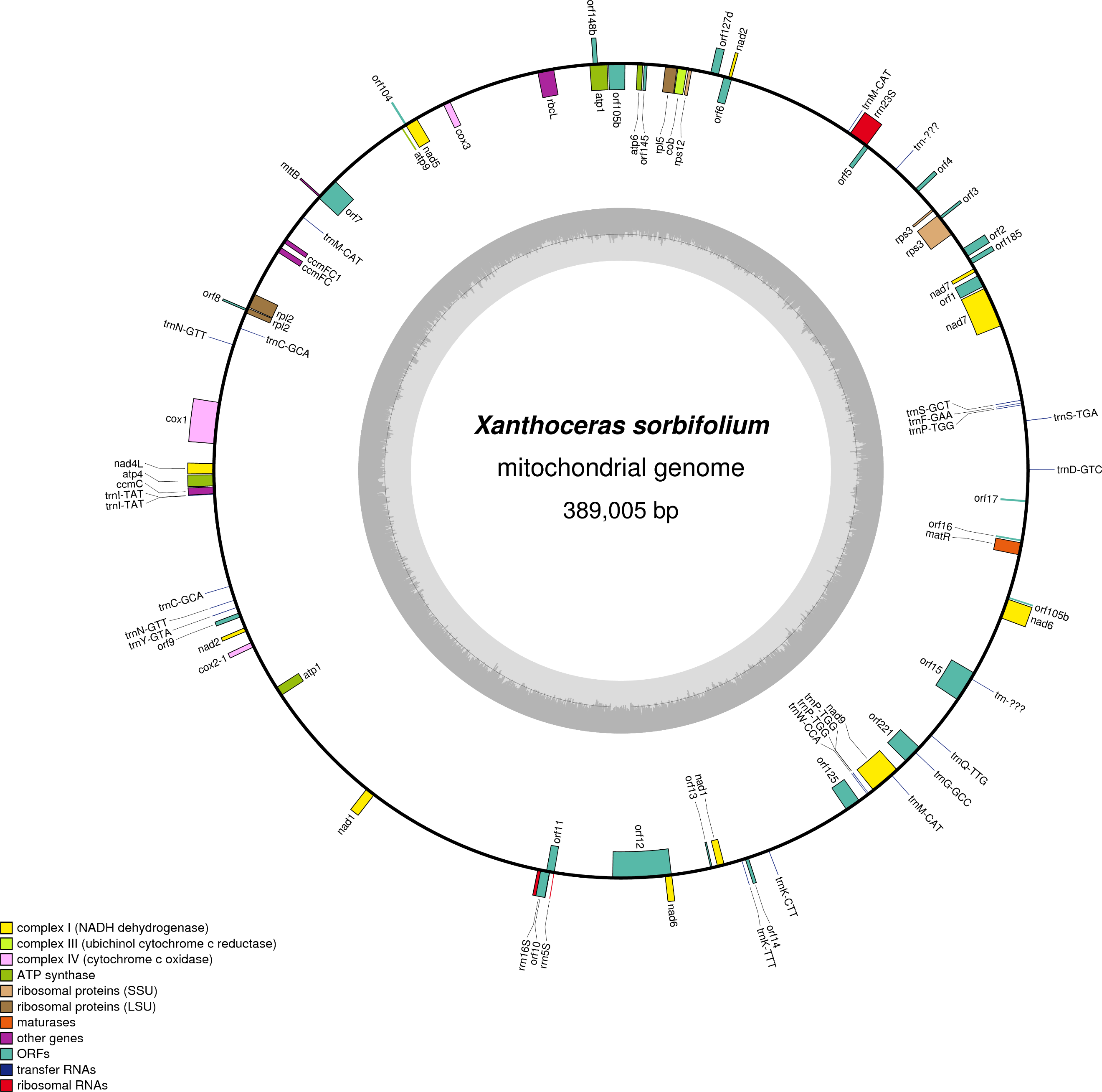


**Supplementary Figure 5. The mitochondrial genome of yellowhorn.** Genes (exons were closed boxes) shown on the outside of the circle were transcribed clockwise, whereas those on the inside were transcribed counter-clockwise. Genes from the same protein complex were colored the same, introns were indicated in white boxes.

**
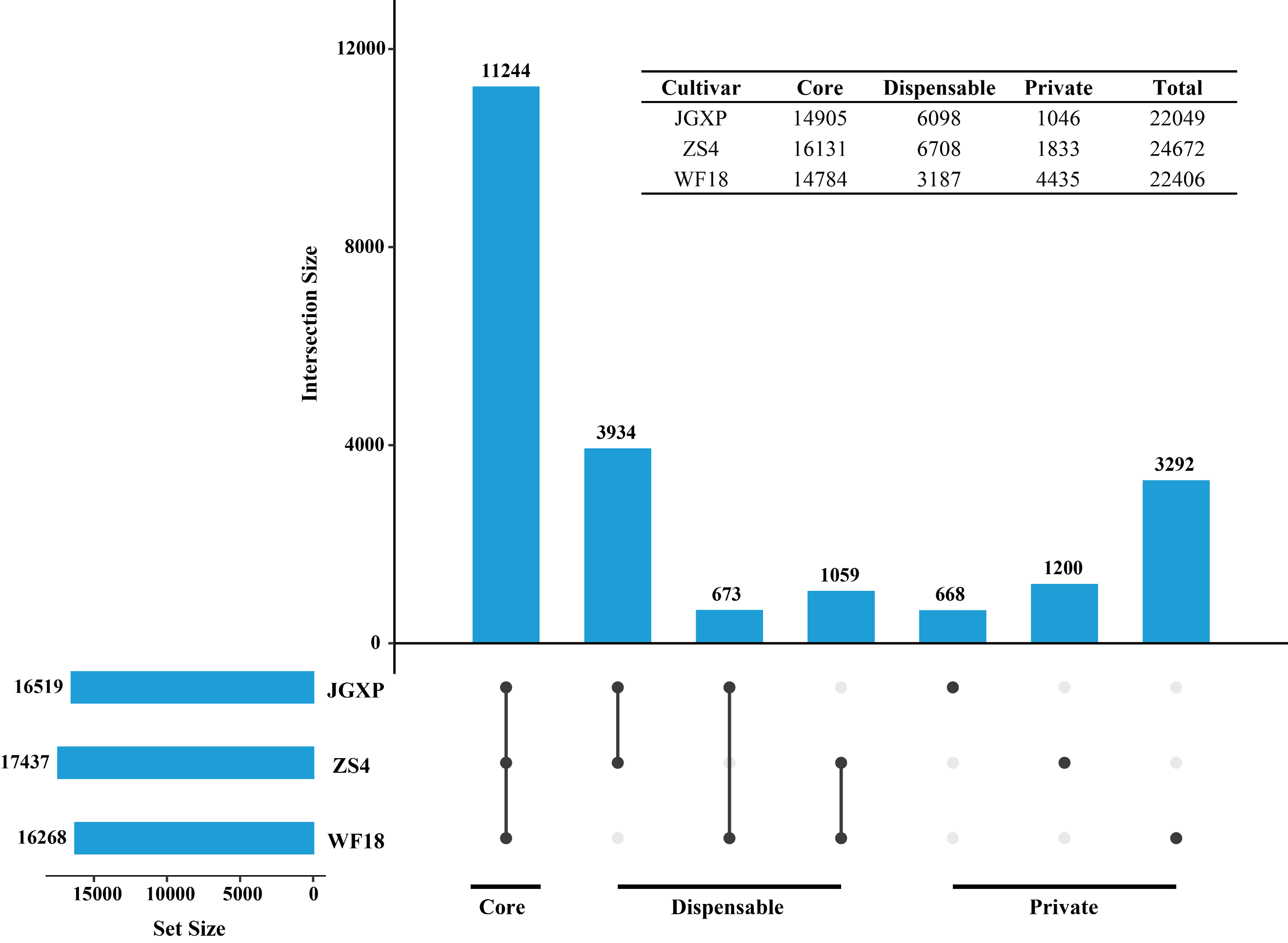
**

**Supplementary Figure 6. An Upset plot showing the number of the core, dispensable, and private gene families among the three yellowhorn assemblies of “JGXP”, “ZS4”, and “WF18”.** The table within the plot showing the number of the core, dispensable, and private genes of the three genomes of yellowhorn. Core, gene families or genes present in all 3 cultivars; Dispensable, gene families or genes present in two cultivars; Private, gene families or genes present in only one cultivar.


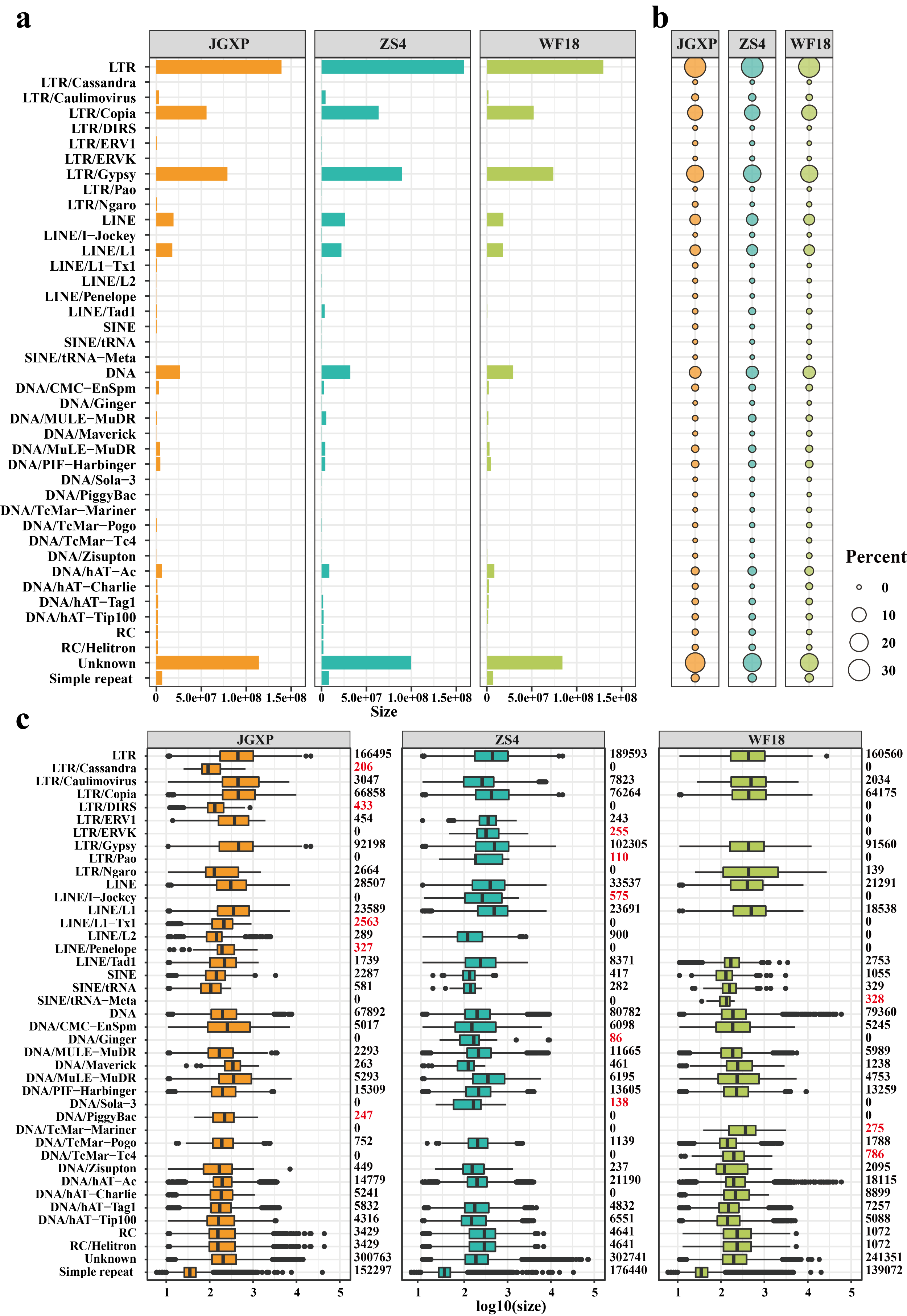


**Supplementary Figure 7. The statistics of repeat elements for the three yellowhorn assemblies of “JGXP”, “ZS4”, and “WF18”.** (a) Barplot showing the total size of repeat elements among the three assemblies of “JGXP”, “ZS4”, and “WF18”. (b) The percentage of repeat elements among the three assemblies. The size of the circles represents the percentage of repeat elements in the assembly. (c) Boxplot of the size of repeat elements among the three assemblies. The numbers represent the total number of repeat elements and those highlight with red indicate the cultivar-specific repeat elements.

**
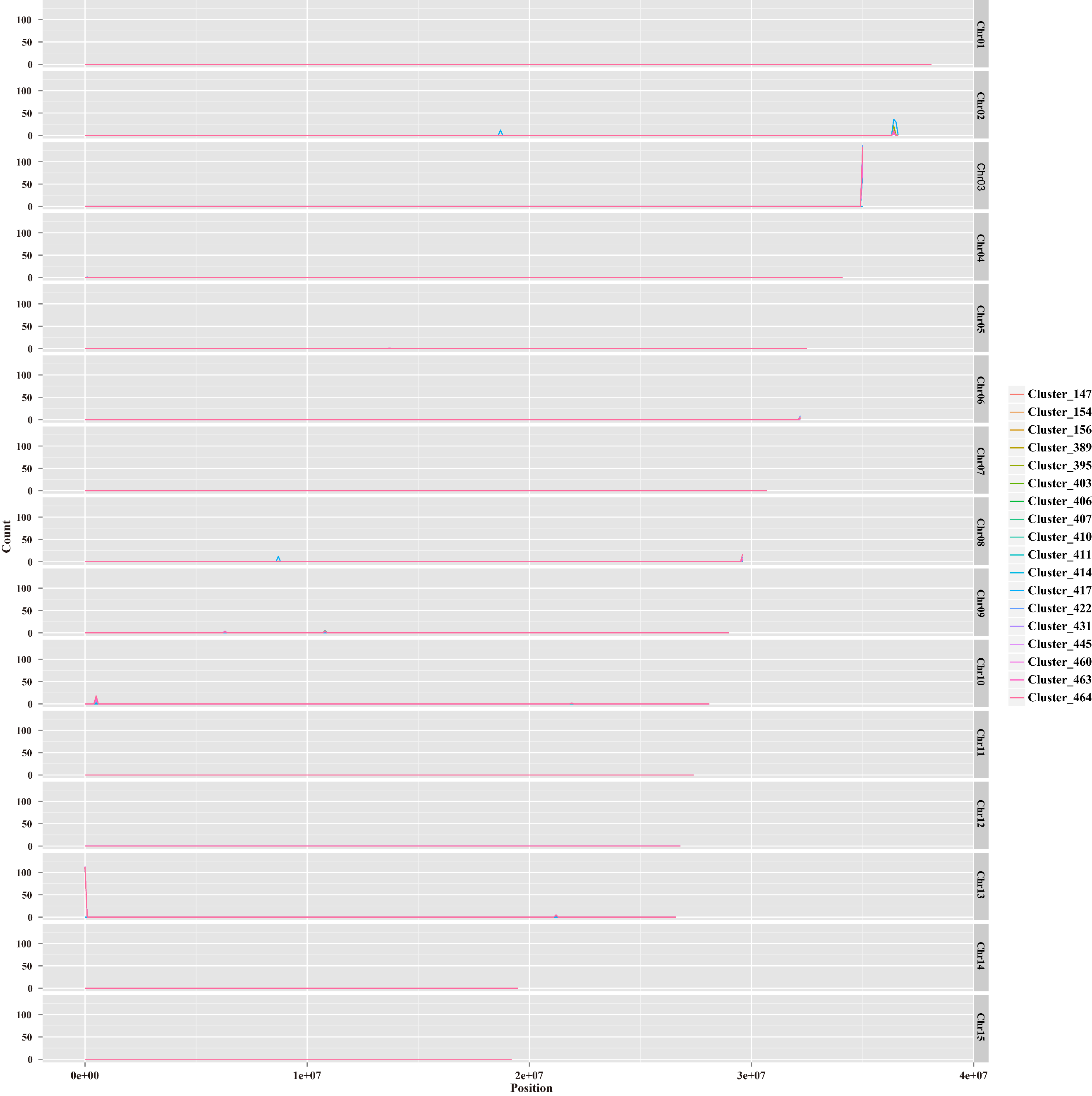
**

**Supplementary Figure 8. The distribution of tandem repeats identified from PacBio long reads along the 15 chromosomes.**

**
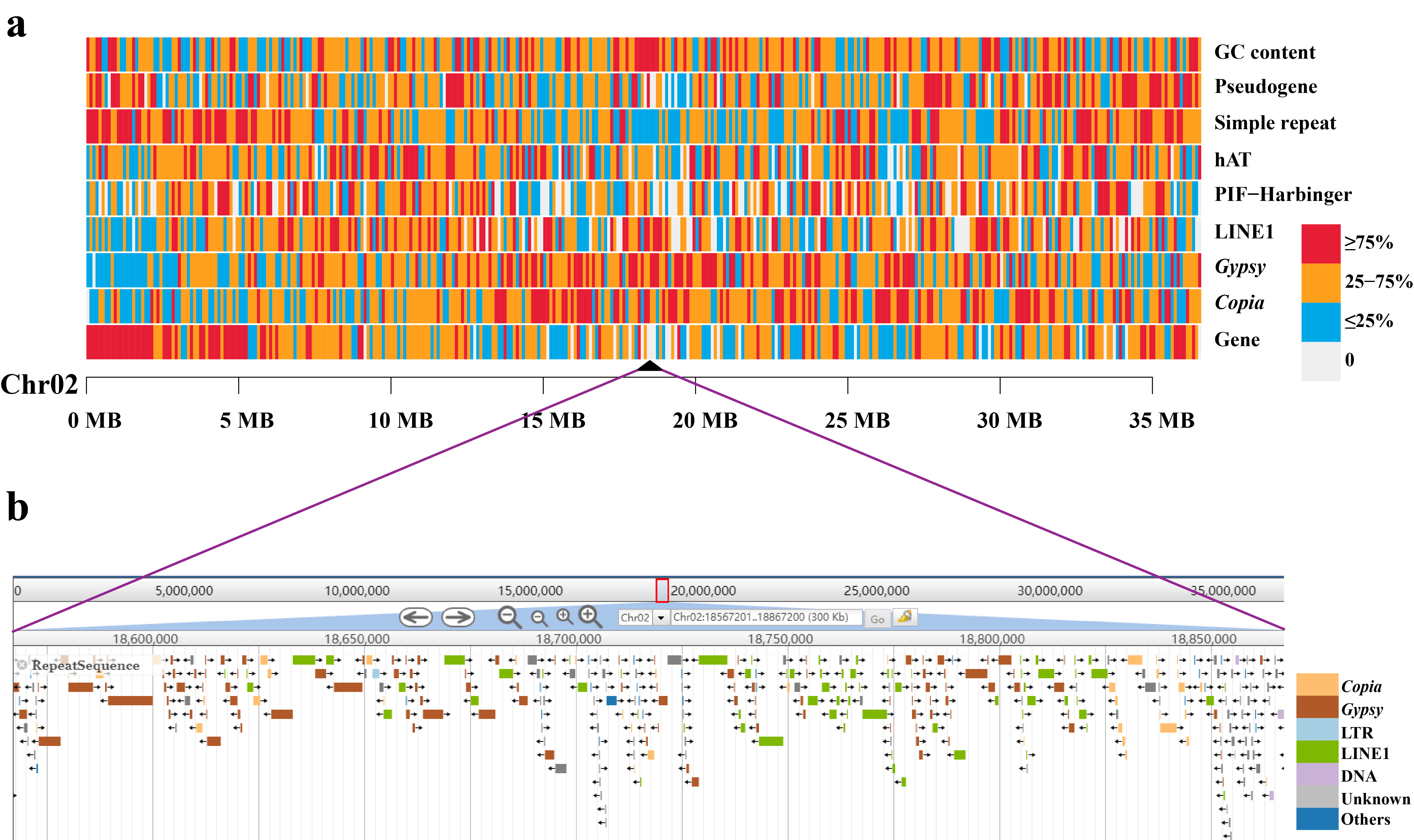
**

**Supplementary Figure 9. The density of genomic feature and the zoom in on the centromeric region for “Chr02”.** (a) Heat map view of genes, TE (*Copia*, *Gypsy*, LINE1, PIF-Harbinger, hAT), simple repeat, pseudogene, and GC content density in 100 Kb non-overlap windows. The black triangle represents the predicted location of centromere. (b) The zoom in on the centromeric region was showed.

**
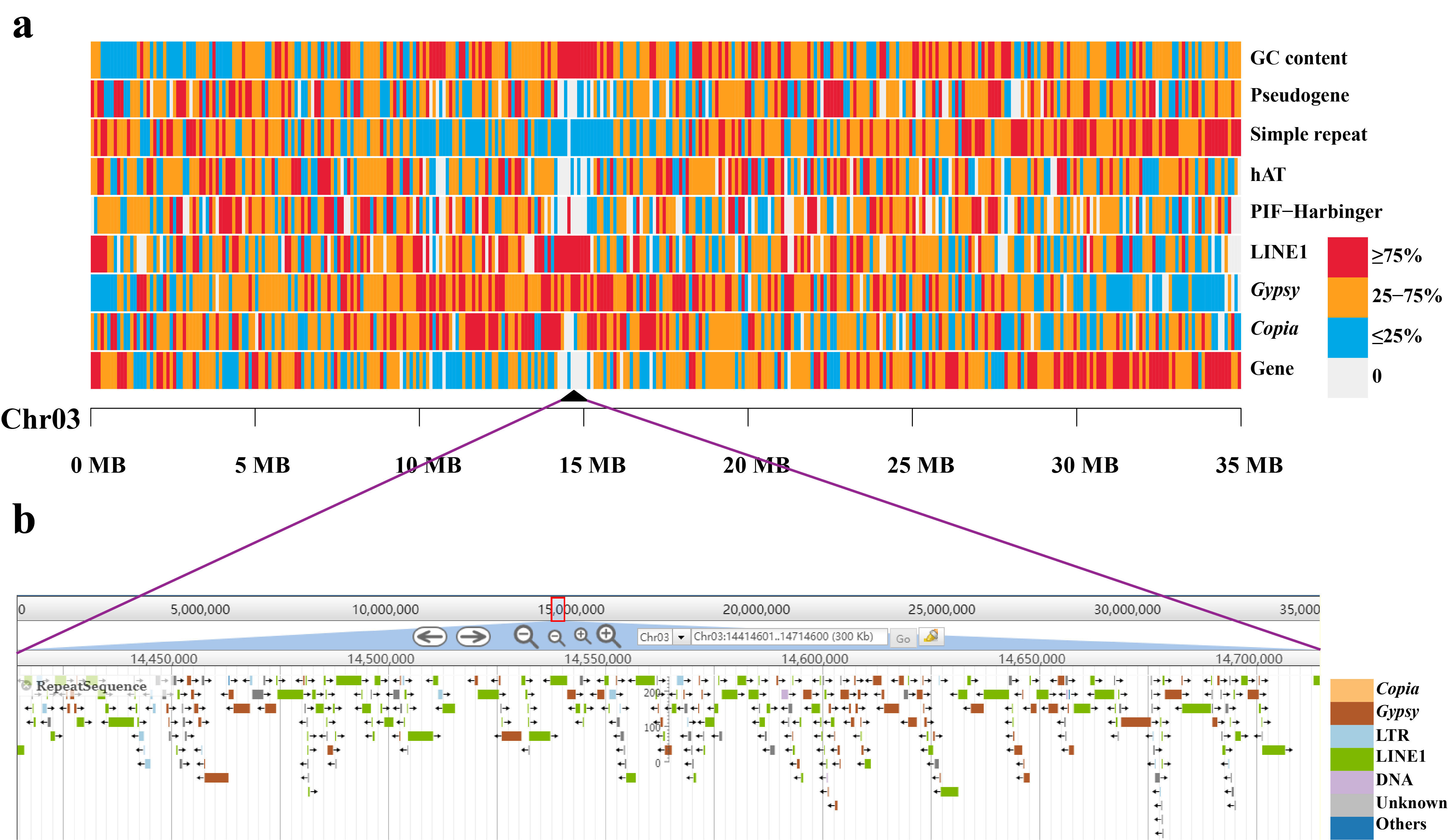
**

**Supplementary Figure 10. The density of genomic feature and the zoom in on the centromeric region for “Chr03”.** (a) Heat map view of genes, TE (*Copia*, *Gypsy*, LINE1, PIF-Harbinger, hAT), simple repeat, pseudogene, and GC content density in 100 Kb non-overlap windows. The black triangle represents the predicted location of centromere. (b) The zoom in on the centromeric region was showed.

**
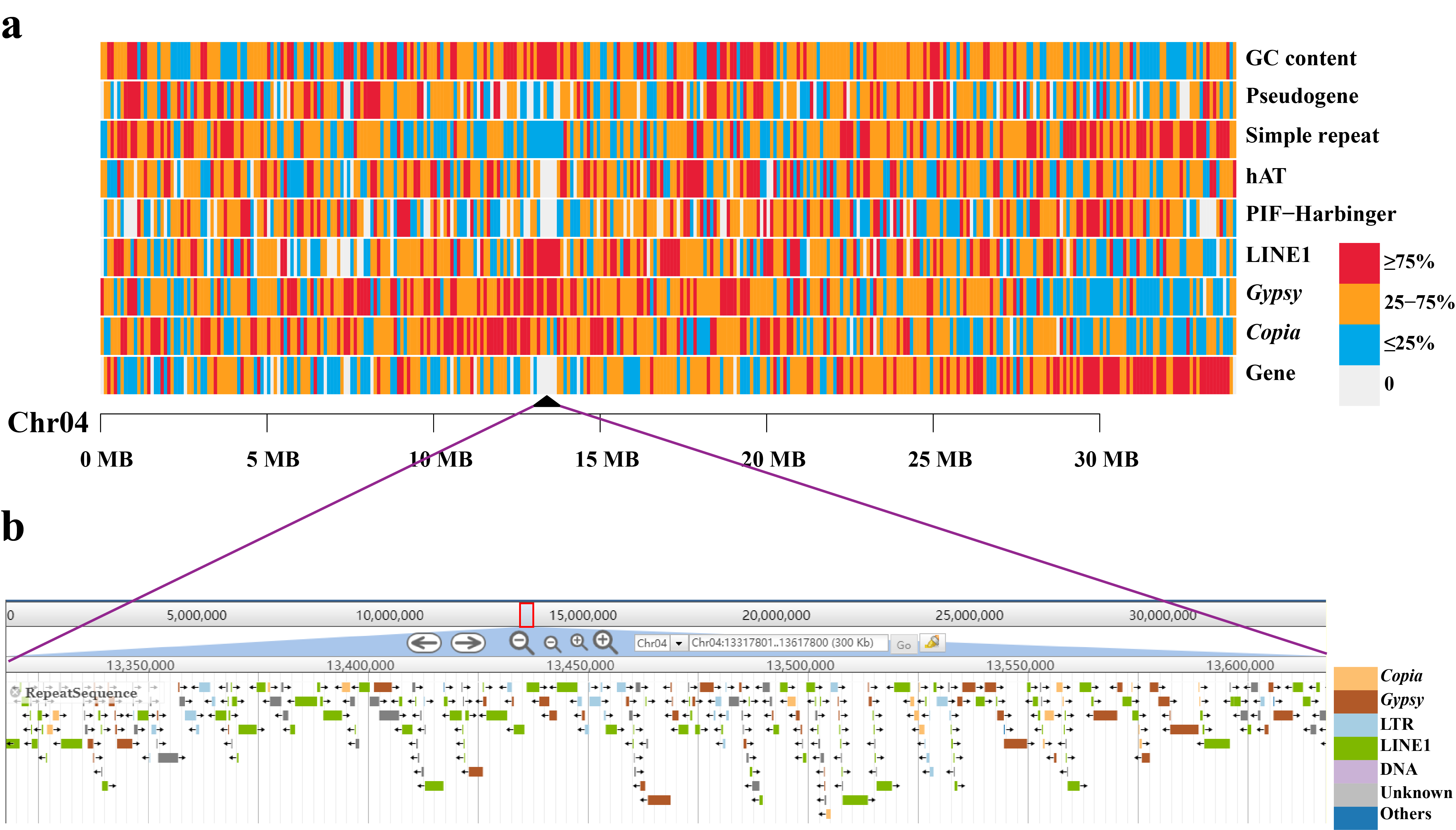
**

**Supplementary Figure 11. The density of genomic feature and the zoom in on the centromeric region for “Chr04”.** (a) Heat map view of genes, TE (*Copia*, *Gypsy*, LINE1, PIF-Harbinger, hAT), simple repeat, pseudogene, and GC content density in 100 Kb non-overlap windows. The black triangle represents the predicted location of centromere. (b) The zoom in on the centromeric region was showed.

**
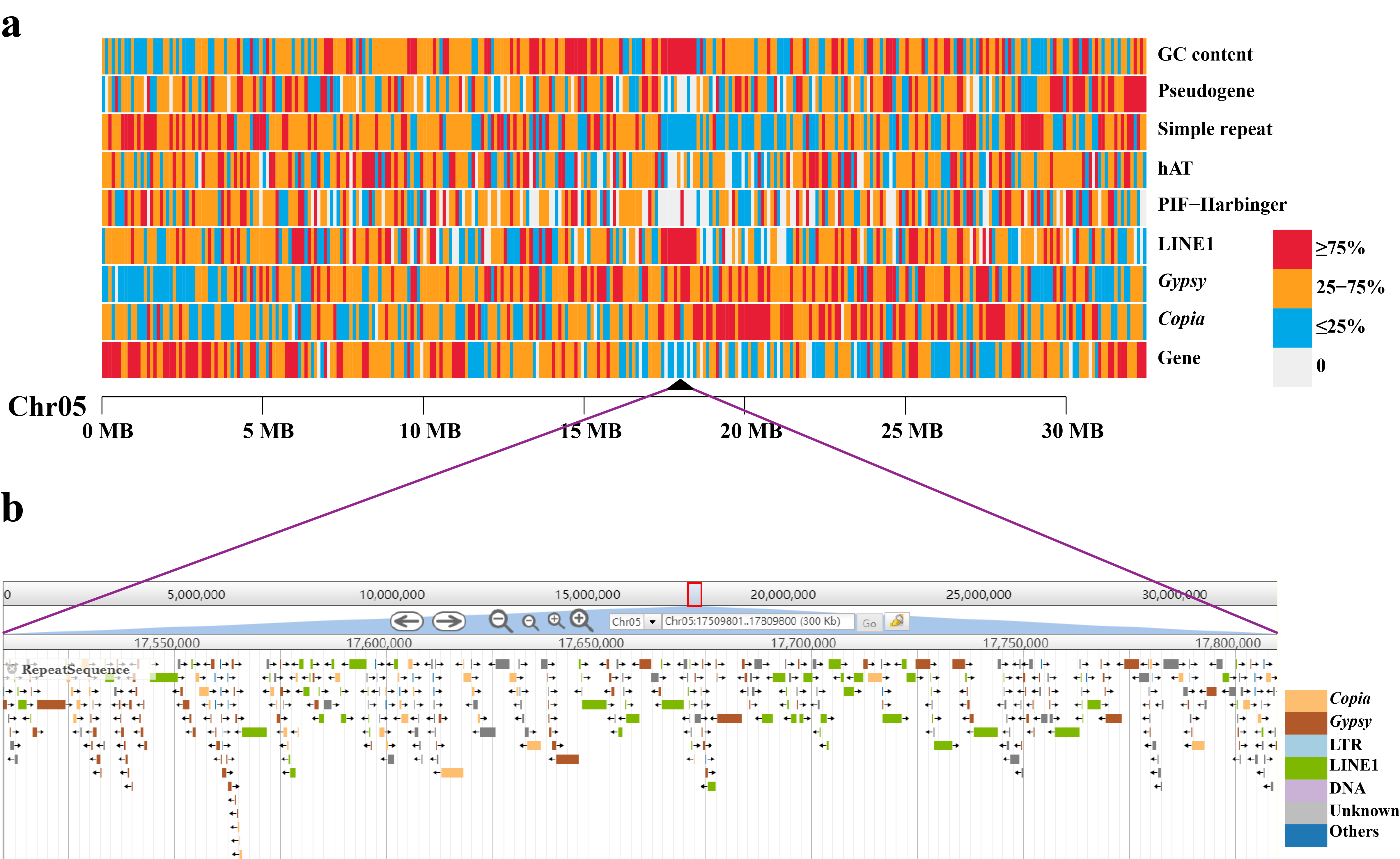
**

**Supplementary Figure 12. The density of genomic feature and the zoom in on the centromeric region for “Chr05”.** (a) Heat map view of genes, TE (*Copia*, *Gypsy*, LINE1, PIF-Harbinger, hAT), simple repeat, pseudogene, and GC content density in 100 Kb non-overlap windows. The black triangle represents the predicted location of centromere. (b) The zoom in on the centromeric region was showed.

**
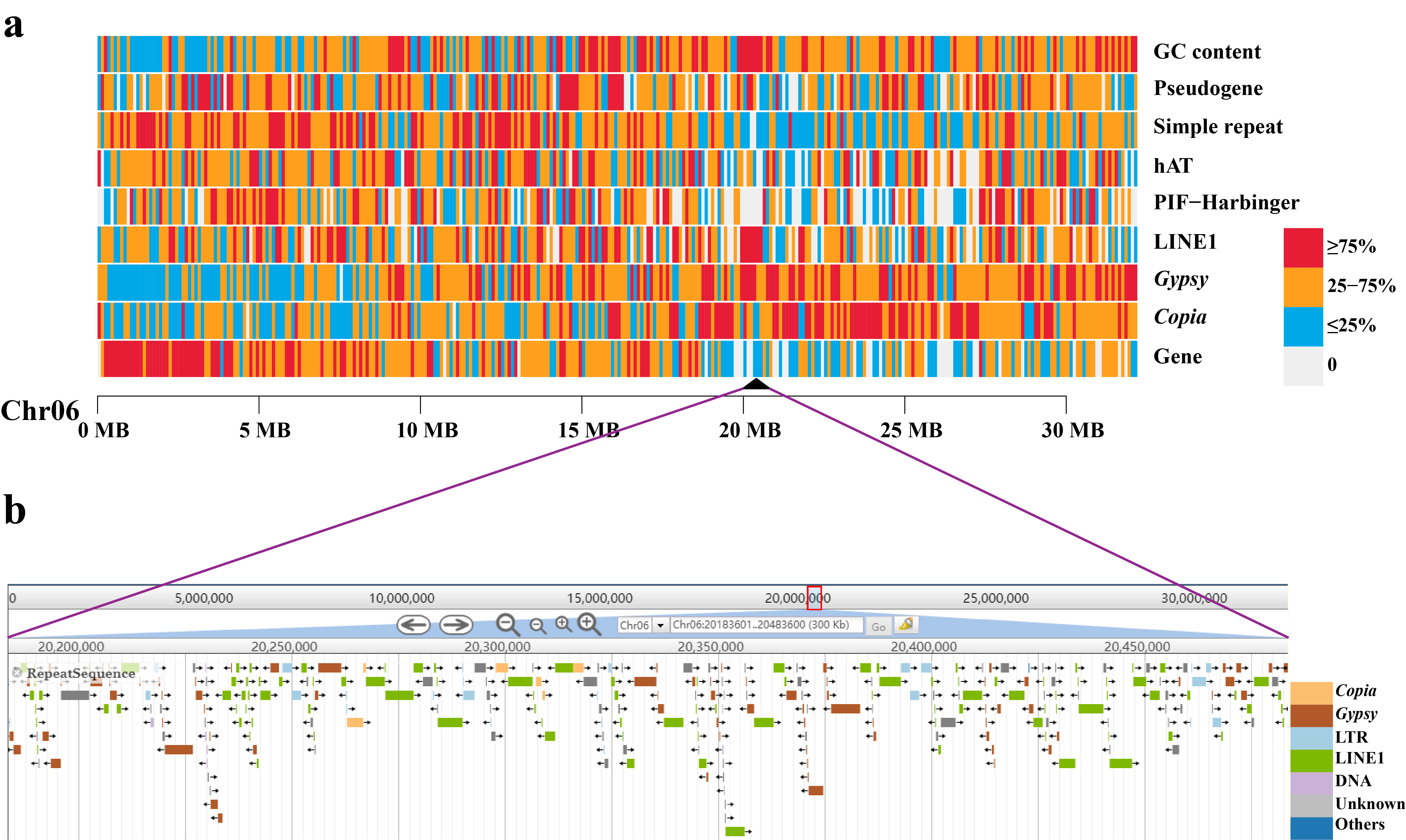
**

**Supplementary Figure 13. The density of genomic feature and the zoom in on the centromeric region for “Chr06”.** (a) Heat map view of genes, TE (*Copia*, *Gypsy*, LINE1, PIF-Harbinger, hAT), simple repeat, pseudogene, and GC content density in 100 Kb non-overlap windows. The black triangle represents the predicted location of centromere. (b) The zoom in on the centromeric region was showed.

**
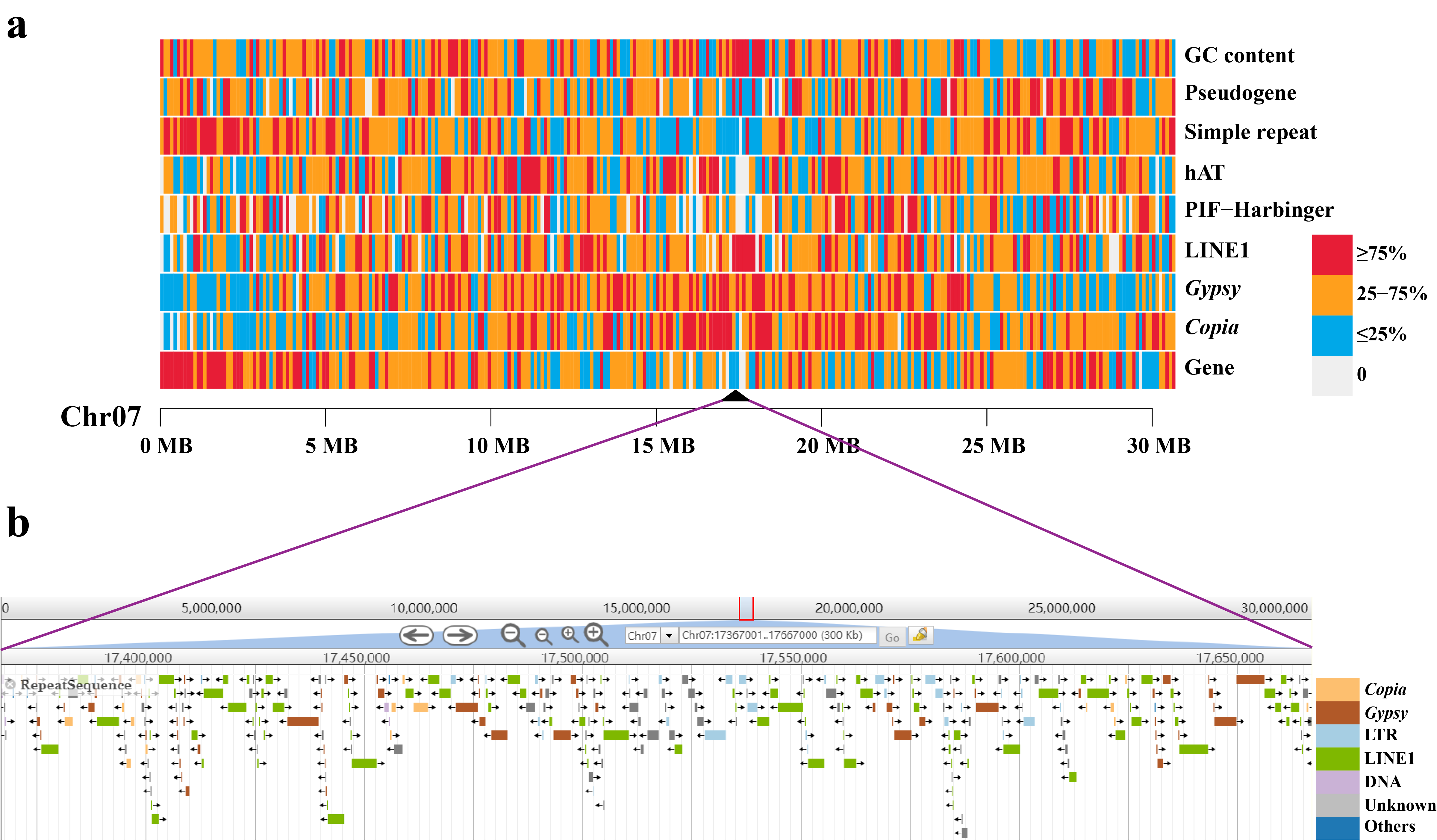
**

**Supplementary Figure 14. The density of genomic feature and the zoom in on the centromeric region for “Chr07”.** (a) Heat map view of genes, TE (*Copia*, *Gypsy*, LINE1, PIF-Harbinger, hAT), simple repeat, pseudogene, and GC content density in 100 Kb non-overlap windows. The black triangle represents the predicted of location centromere. (b) The zoom in on the centromeric region was showed.

**
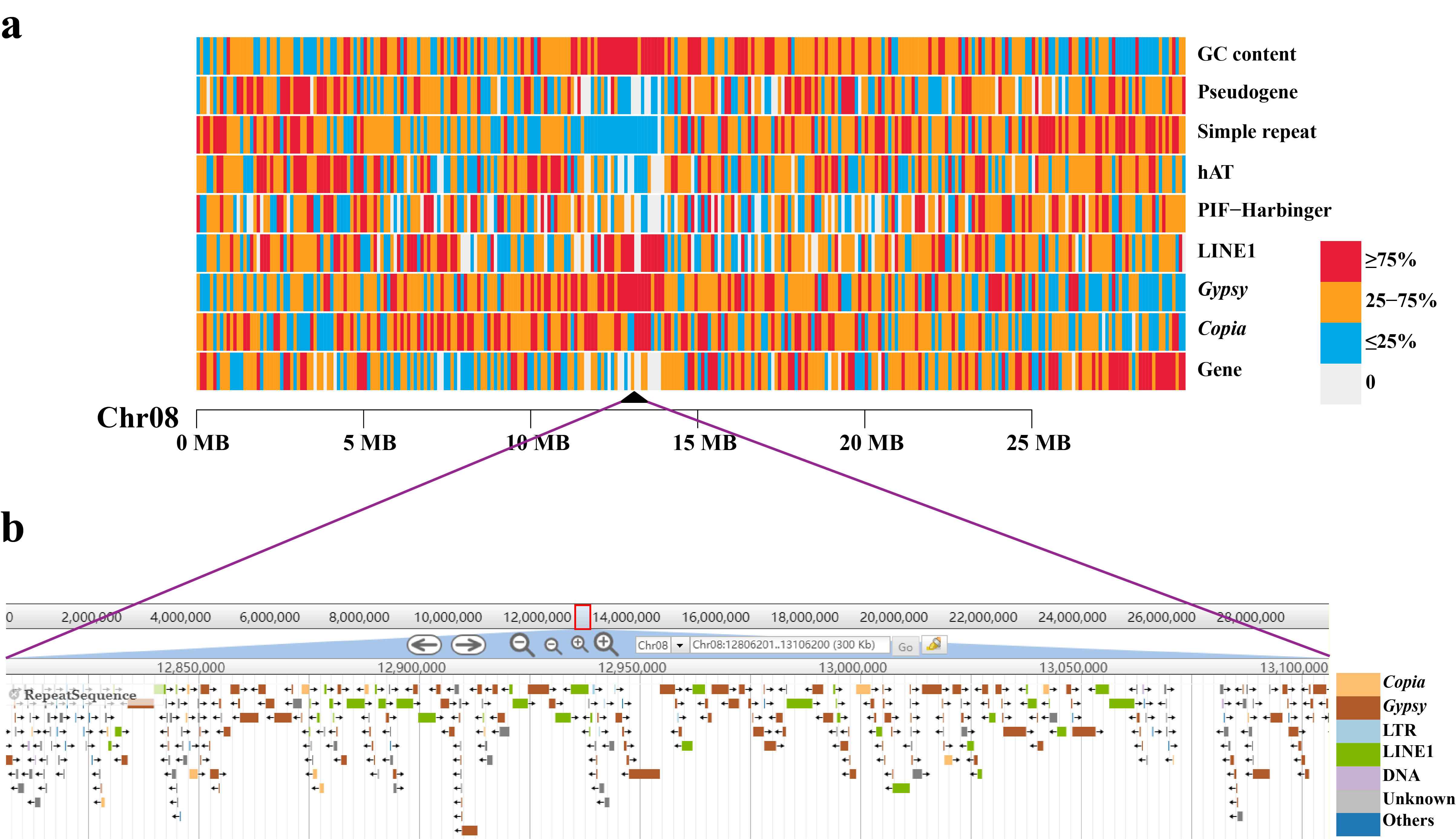
**

**Supplementary Figure 15. The density of genomic feature and the zoom in on the centromeric region for “Chr08”.** (a) Heat map view of genes, TE (*Copia*, *Gypsy*, LINE1, PIF-Harbinger, hAT), simple repeat, pseudogene, and GC content density in 100 Kb non-overlap windows. The black triangle represents the predicted location of centromere. (b) The zoom in on the centromeric region was showed.

**
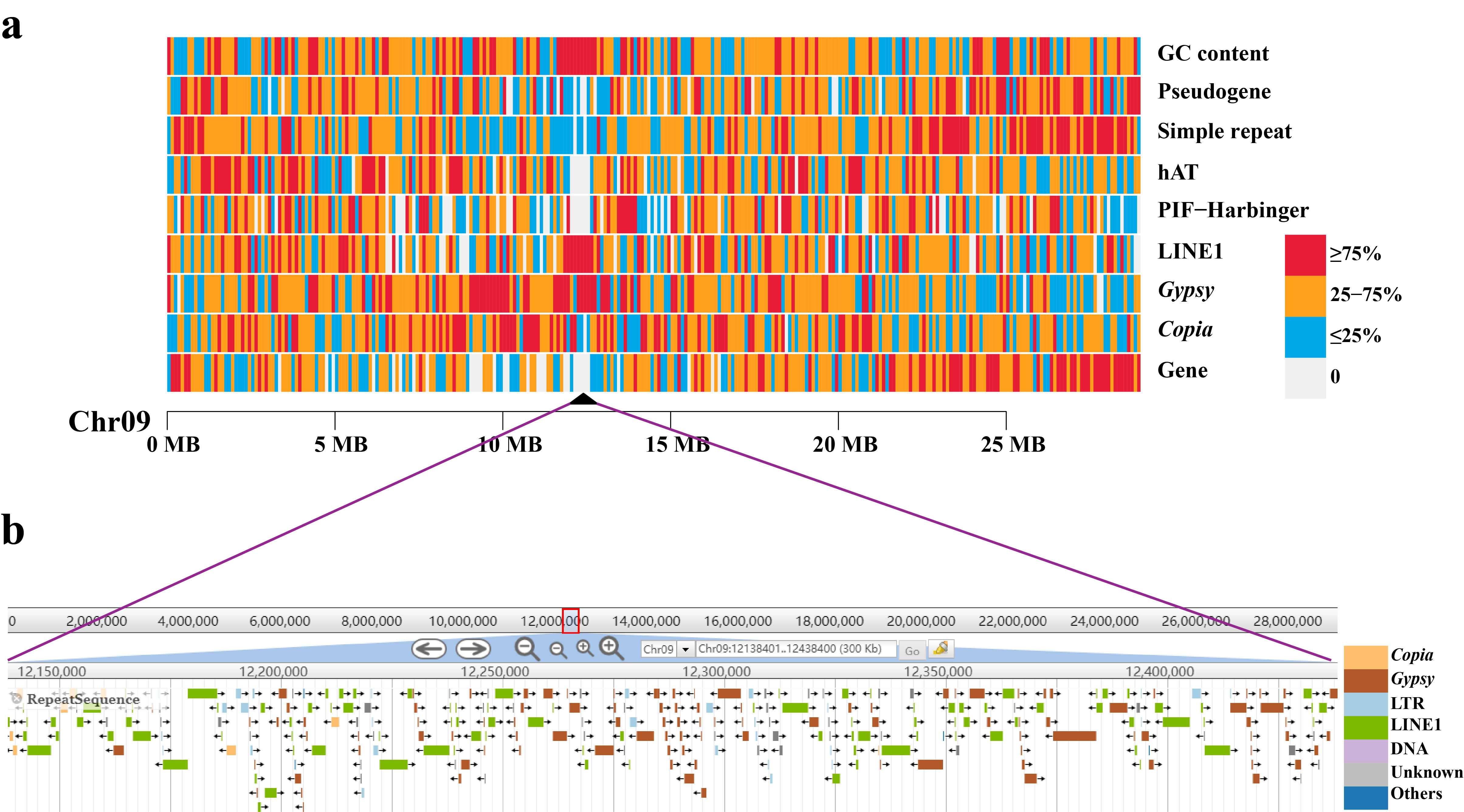
**

**Supplementary Figure 16. The density of genomic feature and the zoom in on the centromeric region for “Chr09”.** (a) Heat map view of genes, TE (*Copia*, *Gypsy*, LINE1, PIF-Harbinger, hAT), simple repeat, pseudogene, and GC content density in 100 Kb non-overlap windows. The black triangle represents the predicted location of centromere. (b) The zoom in on the centromeric region was showed.

**
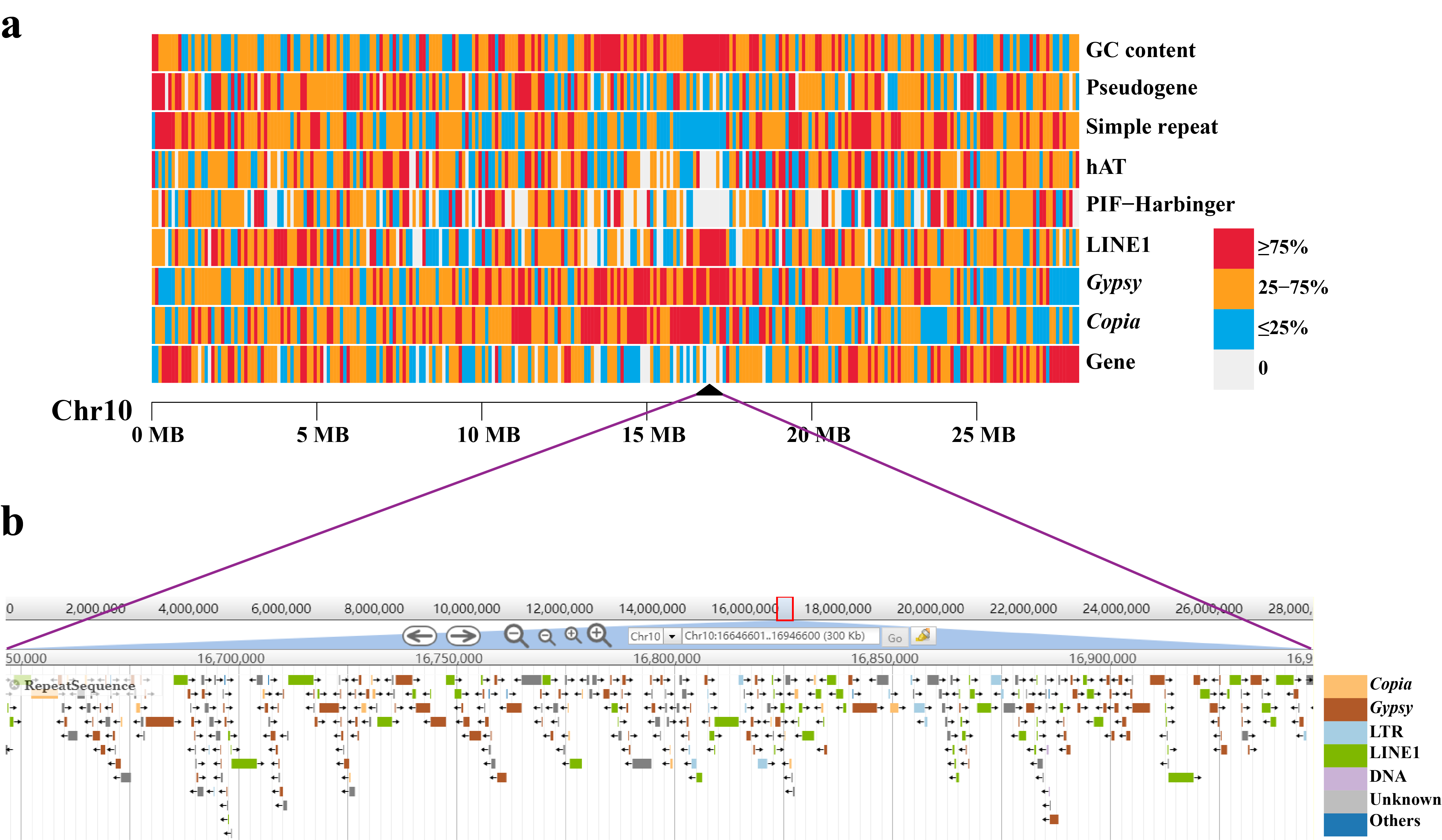
**

**Supplementary Figure 17. The density of genomic feature and the zoom in on the centromeric region for “Chr10”.** (a) Heat map view of genes, TE (*Copia*, *Gypsy*, LINE1, PIF-Harbinger, hAT), simple repeat, pseudogene, and GC content density in 100 Kb non-overlap windows. The black triangle represents the predicted location of centromere. (b) The zoom in on the centromeric region was showed.

**
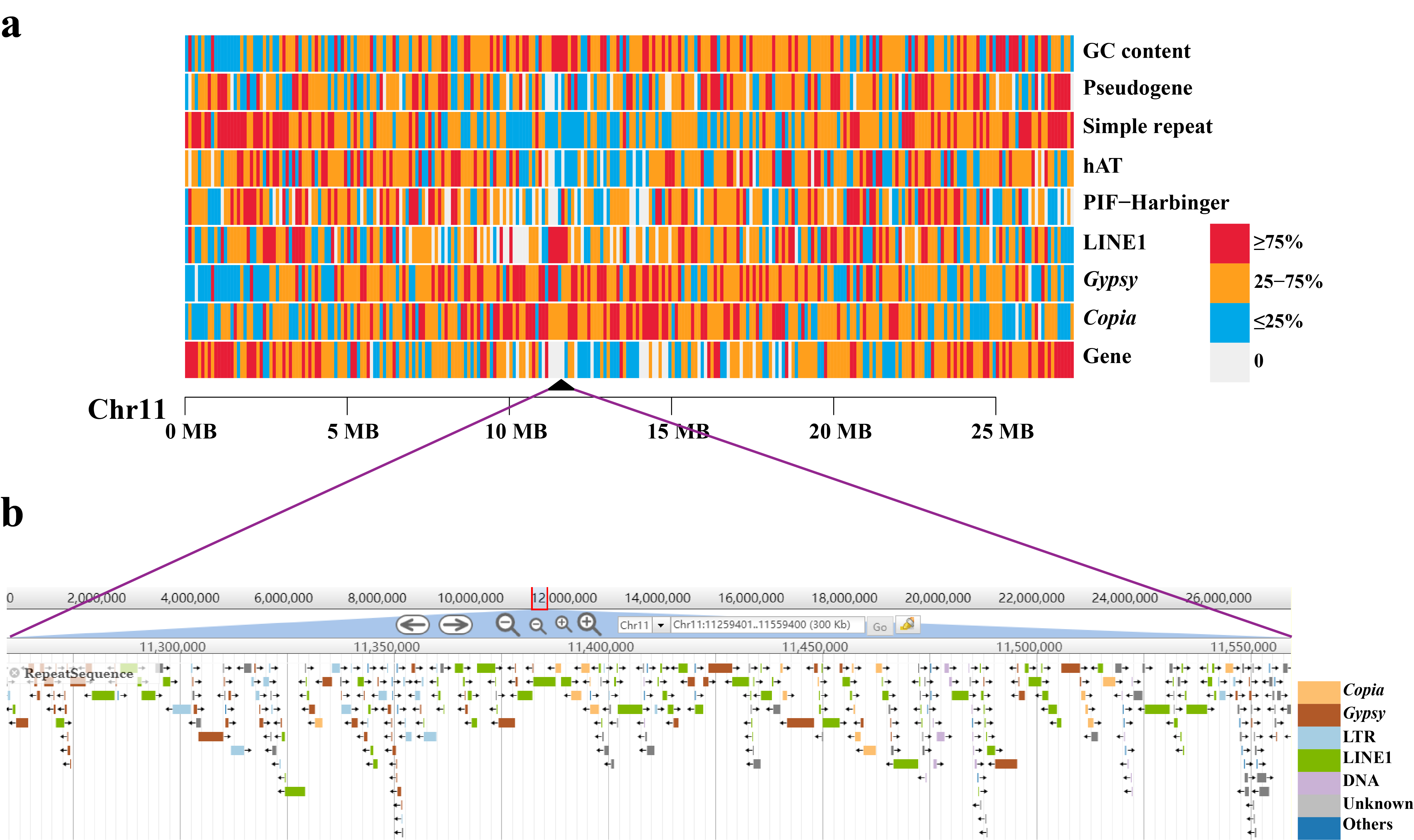
**

**Supplementary Figure 18. The density of genomic feature and the zoom in on the centromeric region for “Chr11”.** (a) Heat map view of genes, TE (*Copia*, *Gypsy*, LINE1, PIF-Harbinger, hAT), simple repeat, pseudogene, and GC content density in 100 Kb non-overlap windows. The black triangle represents the predicted location of centromere. (b) The zoom in on the centromeric region was showed.

**
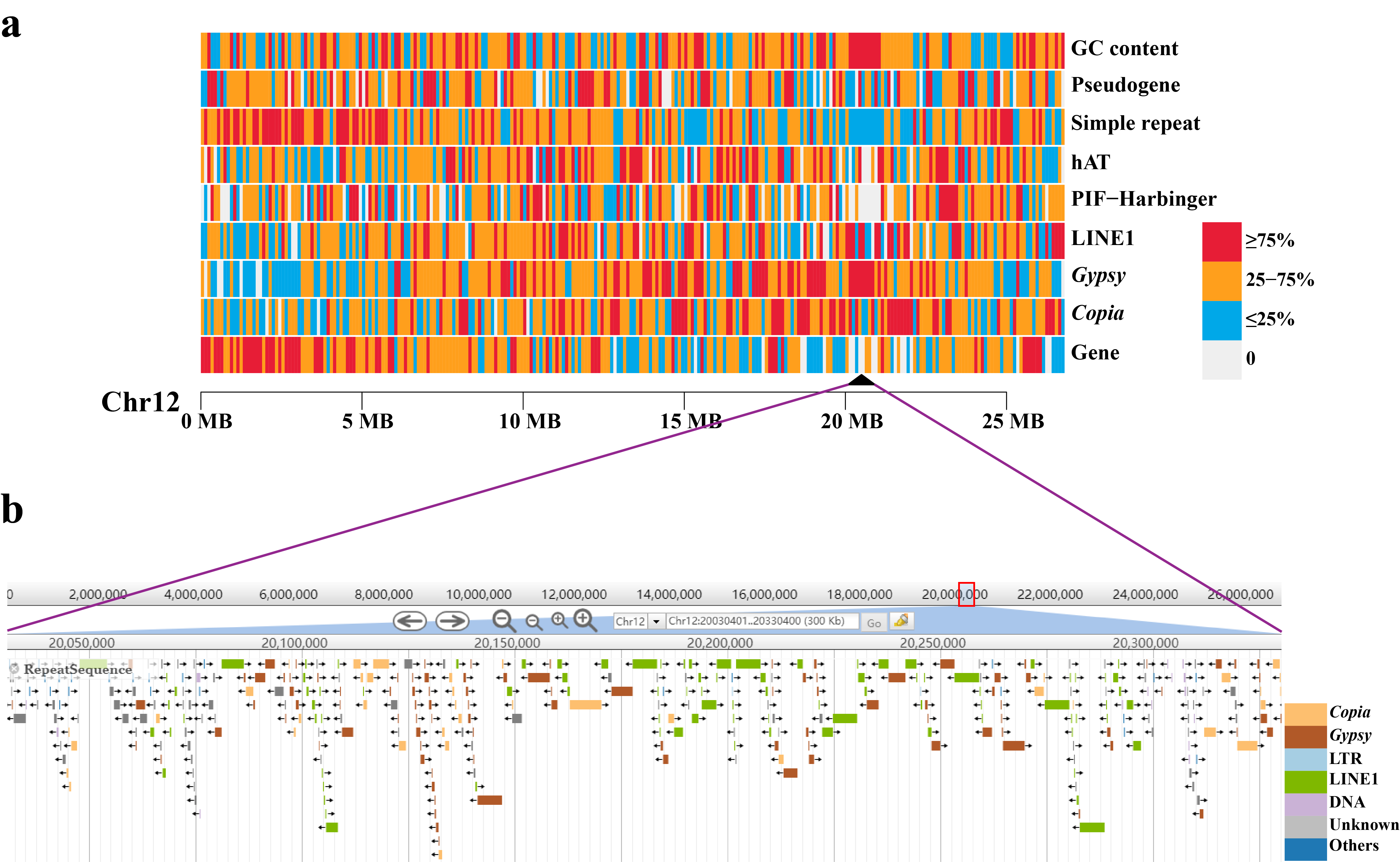
**

**Supplementary Figure 19. The density of genomic feature and the zoom in on the centromeric region for “Chr12”.** (a) Heat map view of genes, TE (*Copia*, *Gypsy*, LINE1, PIF-Harbinger, hAT), simple repeat, pseudogene, and GC content density in 100 Kb non-overlap windows. The black triangle represents the predicted location of centromere. (b) The zoom in on the centromeric region was showed.

**
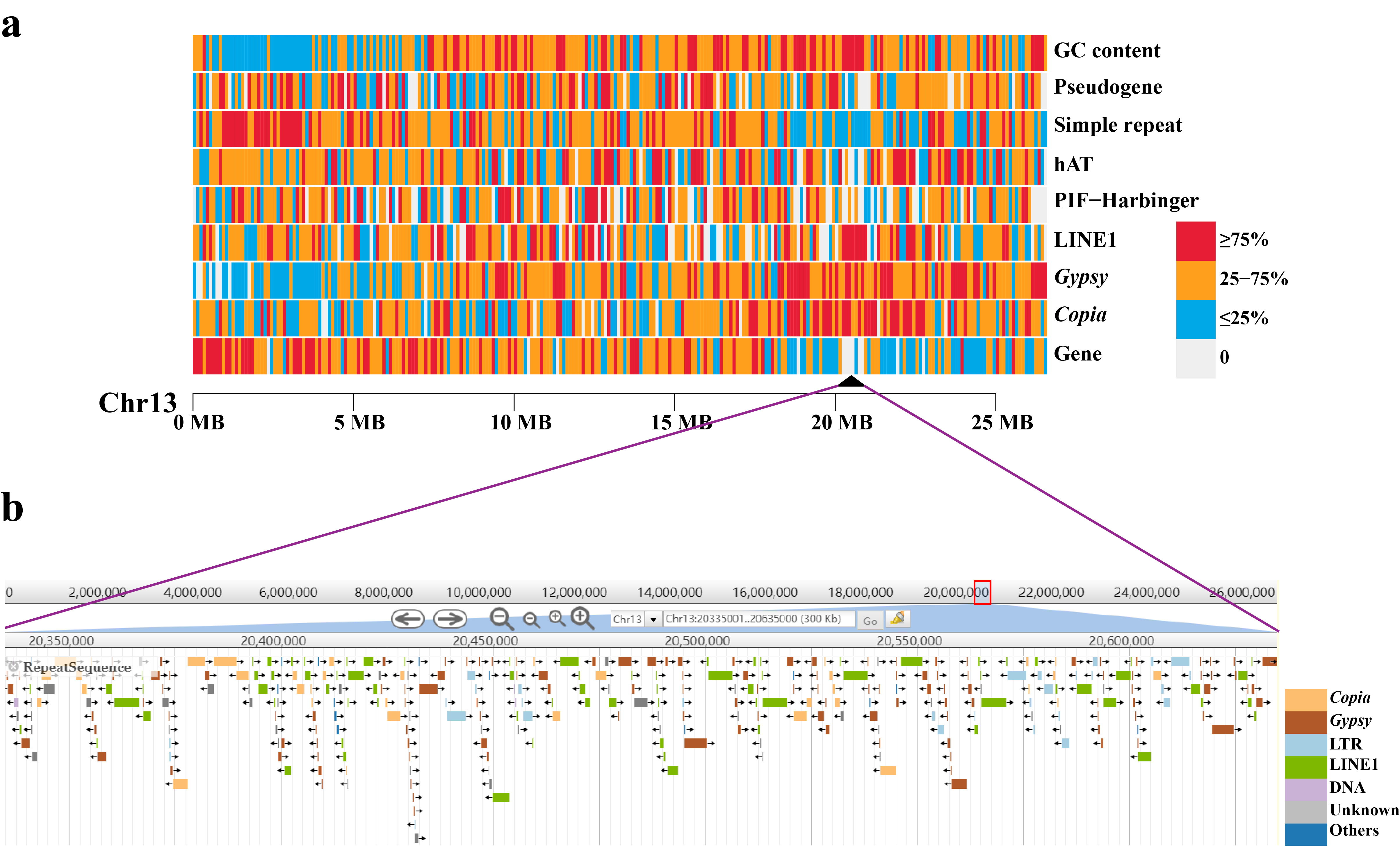
**

**Supplementary Figure 20. The density of genomic feature and the zoom in on the centromeric region for “Chr13”.** (a) Heat map view of genes, TE (*Copia*, *Gypsy*, LINE1, PIF-Harbinger, hAT), simple repeat, pseudogene, and GC content density in 100 Kb non-overlap windows. The black triangle represents the predicted location of centromere. (b) The zoom in on the centromeric region was showed.

**
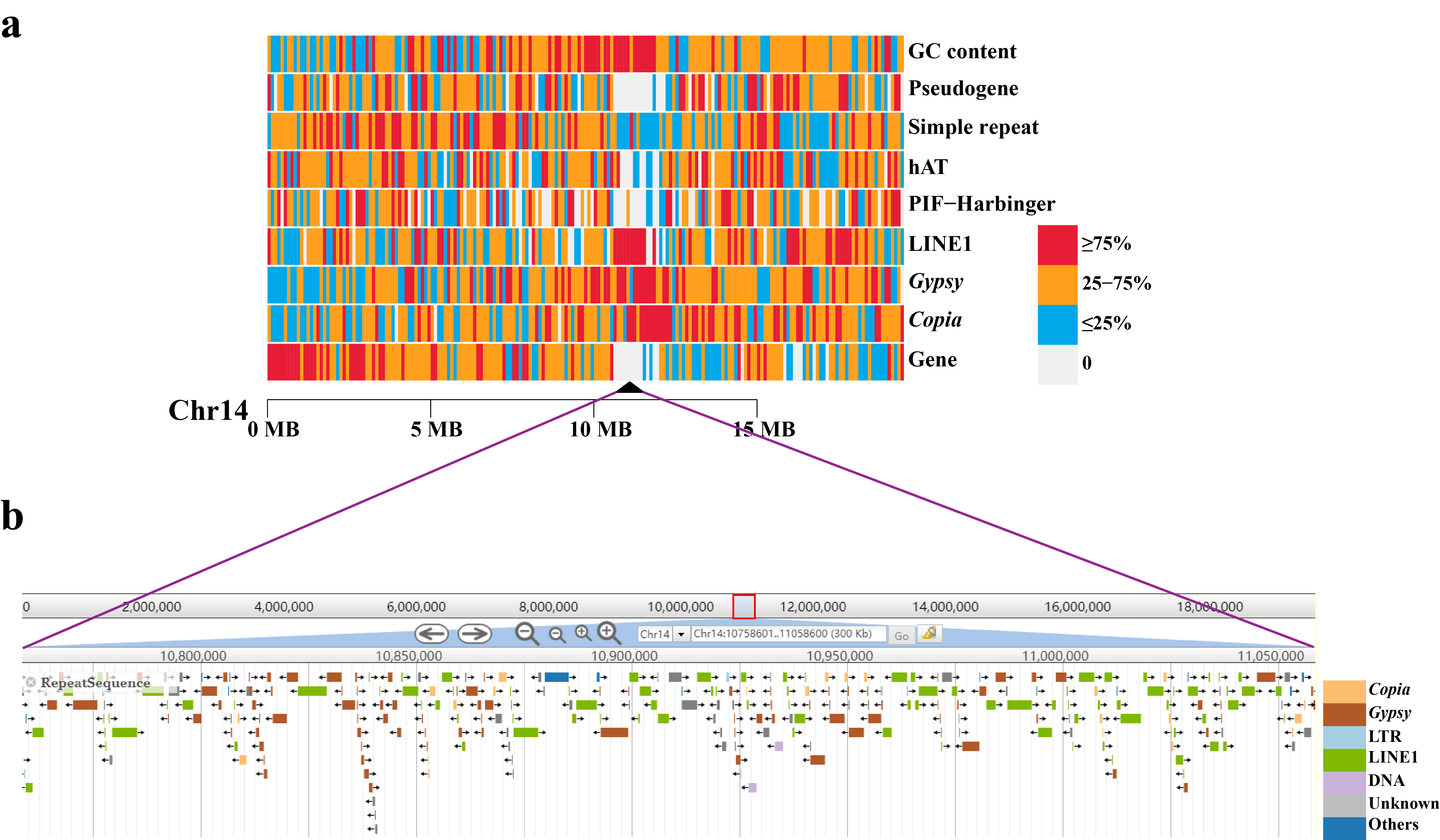
**

**Supplementary Figure 21. The density of genomic feature and the zoom in on the centromeric region for “Chr14”.** (a) Heat map view of genes, TE (*Copia*, *Gypsy*, LINE1, PIF-Harbinger, hAT), simple repeat, pseudogene, and GC content density in 100 Kb non-overlap windows. The black triangle represents the predicted location of centromere. (b) The zoom in on the centromeric region was showed.

**
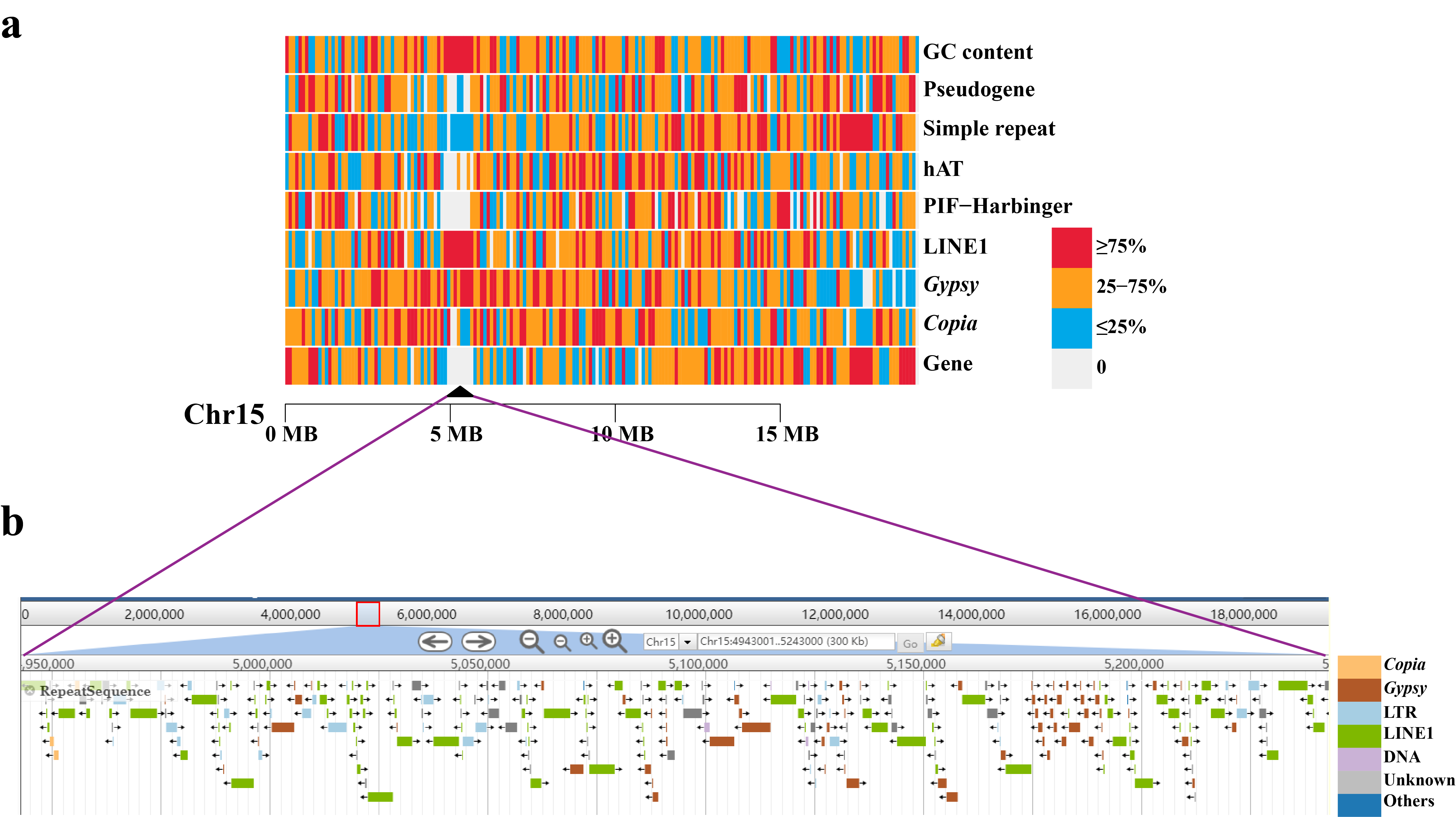
**

**Supplementary Figure 22. The density of genomic feature and the zoom in on the centromeric region for “Chr15”.** (a) Heat map view of genes, TE (*Copia*, *Gypsy*, LINE1, PIF-Harbinger, hAT), simple repeat, pseudogene, and GC content density in 100 Kb non-overlap windows. The black triangle represents the predicted location of centromere. (b) The zoom in on the centromeric region was showed.


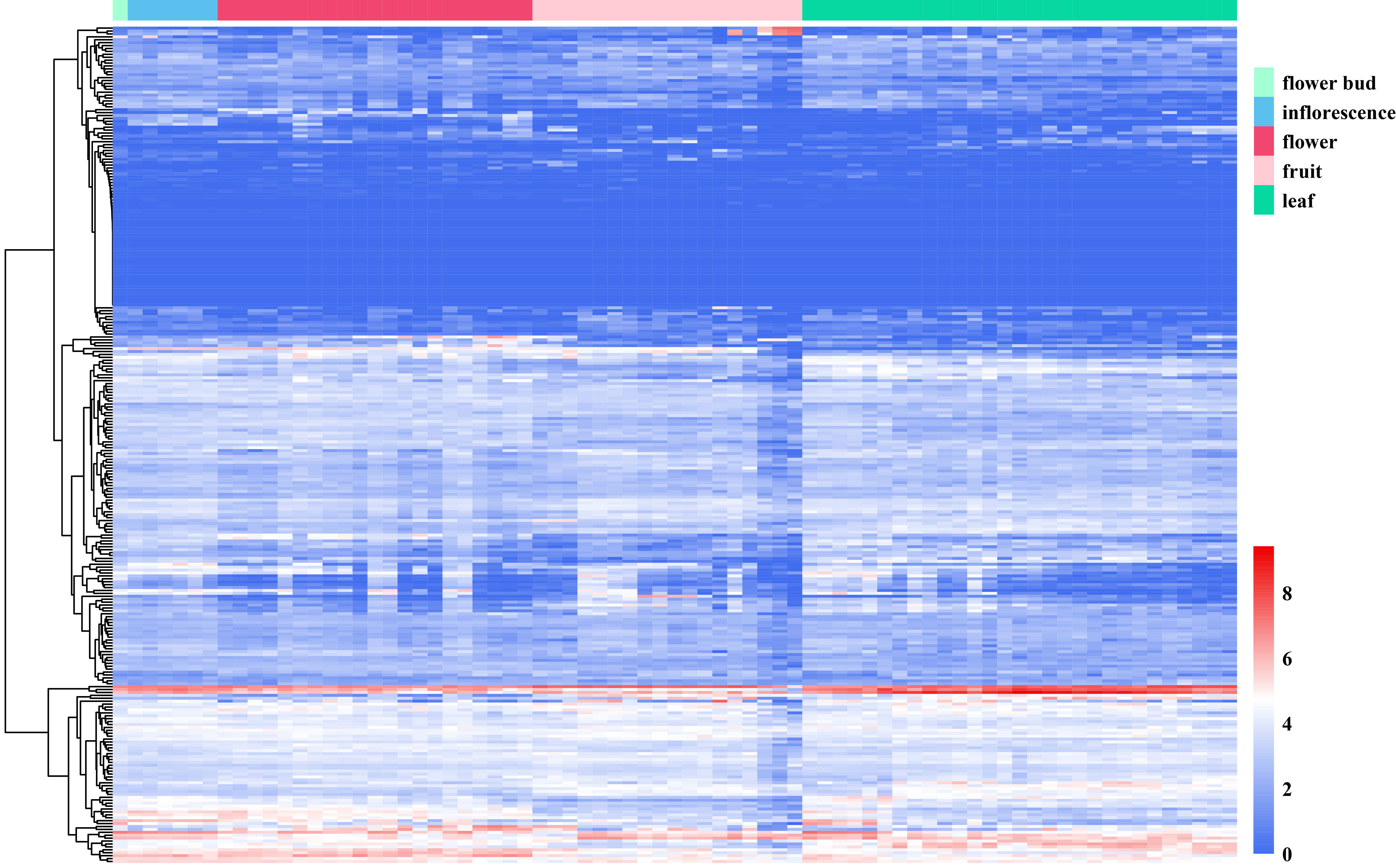


**Supplementary Figure 23. The expression profile of genes in the centromeric regions.** The expression values were normalized by ln(TPM+1). Expression data were provided in Data Files S2.

**
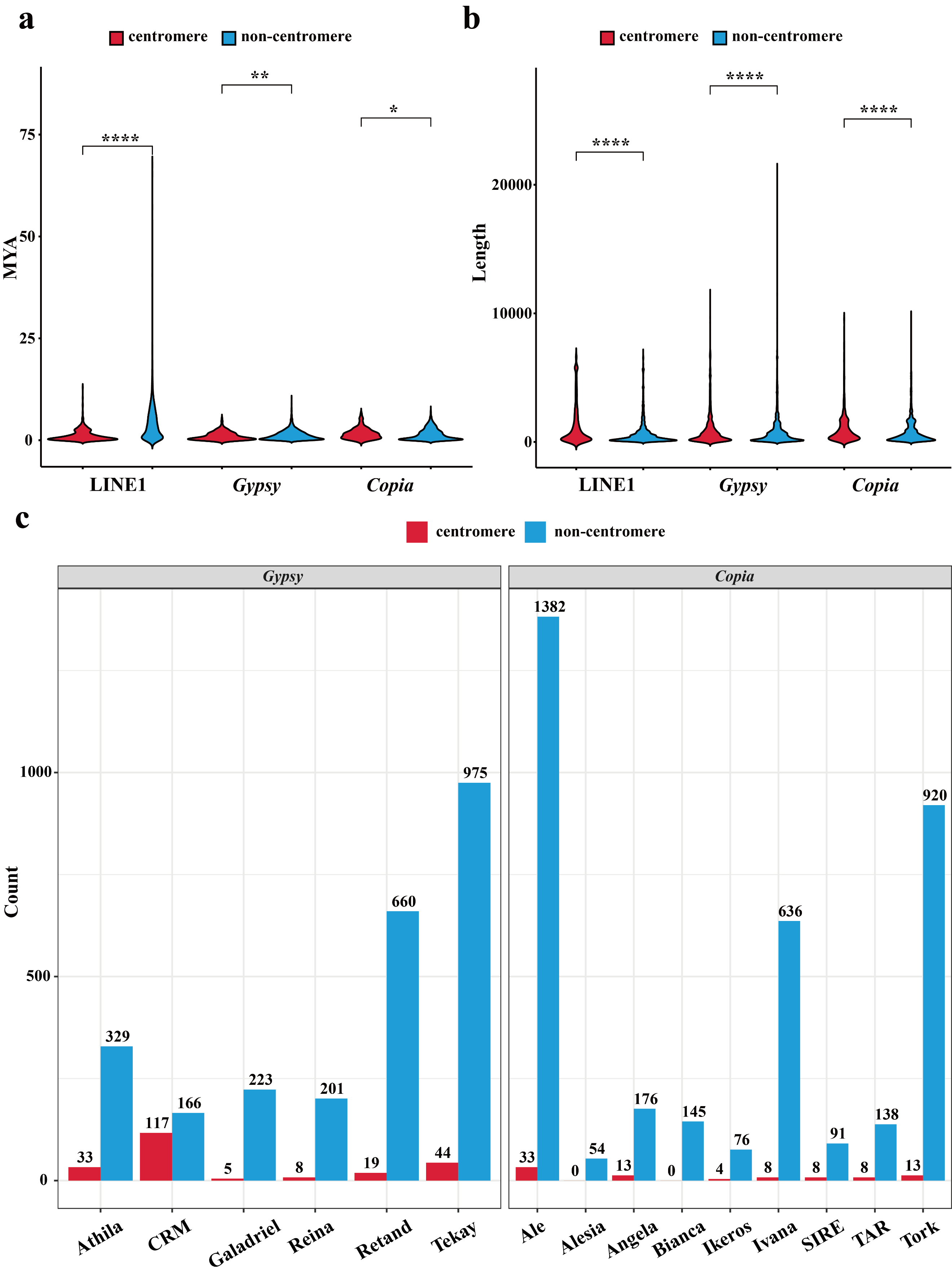
**

**Supplementary Figure 24. The comparison of of LINE1, *Gypsy*, and *Copia* elements between the centromeric regions and non-centromeric regions.** (a) Wilcoxon’s two-sided test comparison of the insertion time of LINE1, intact *Gypsy*, and intact *Copia* elements between the centromeric regions and non-centromeric regions (**P* < 0.05; ***P* < 0.01; ****P* < 0.001). (b) Wilcoxon’s one-sided test comparison of the length of LINE1, *Gypsy*, and *Copia* elements between centromeric regions and non-centromeric regions. (*****P* < 0.0001). (c) Barplot showing the subfamily of intact *Gypsy* and intact *Copia* retrotransposons in the centromeric regions and non-centromeric regions, respectively.

**
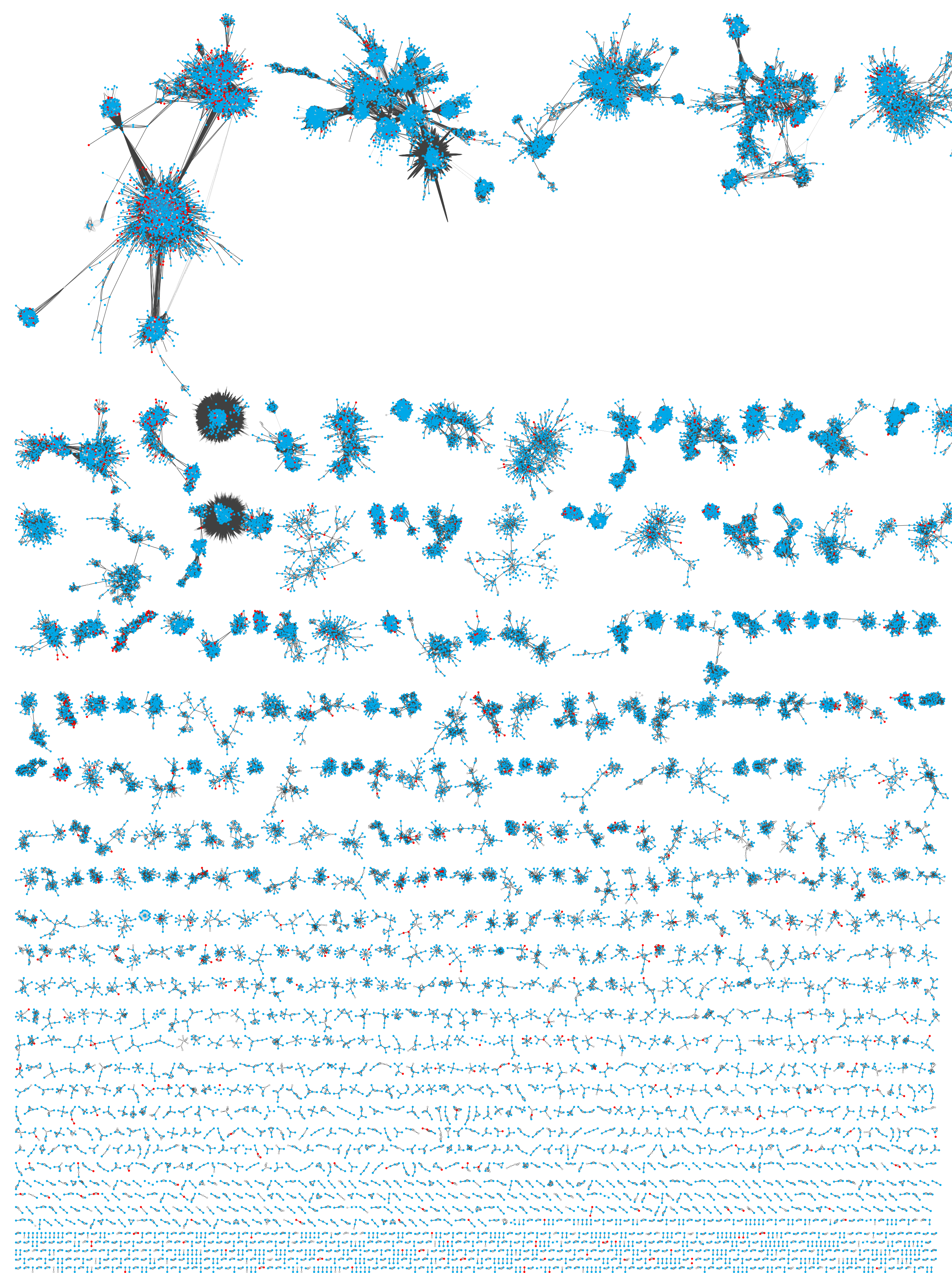
**

**Supplementary Figure 25. The phylogenetic network of *Copia* elements.** Each node in the network represents a single *Copia* sequence (red, *Copia* in centromeric regions; blue, *Copia* in non-centromeric regions).

**
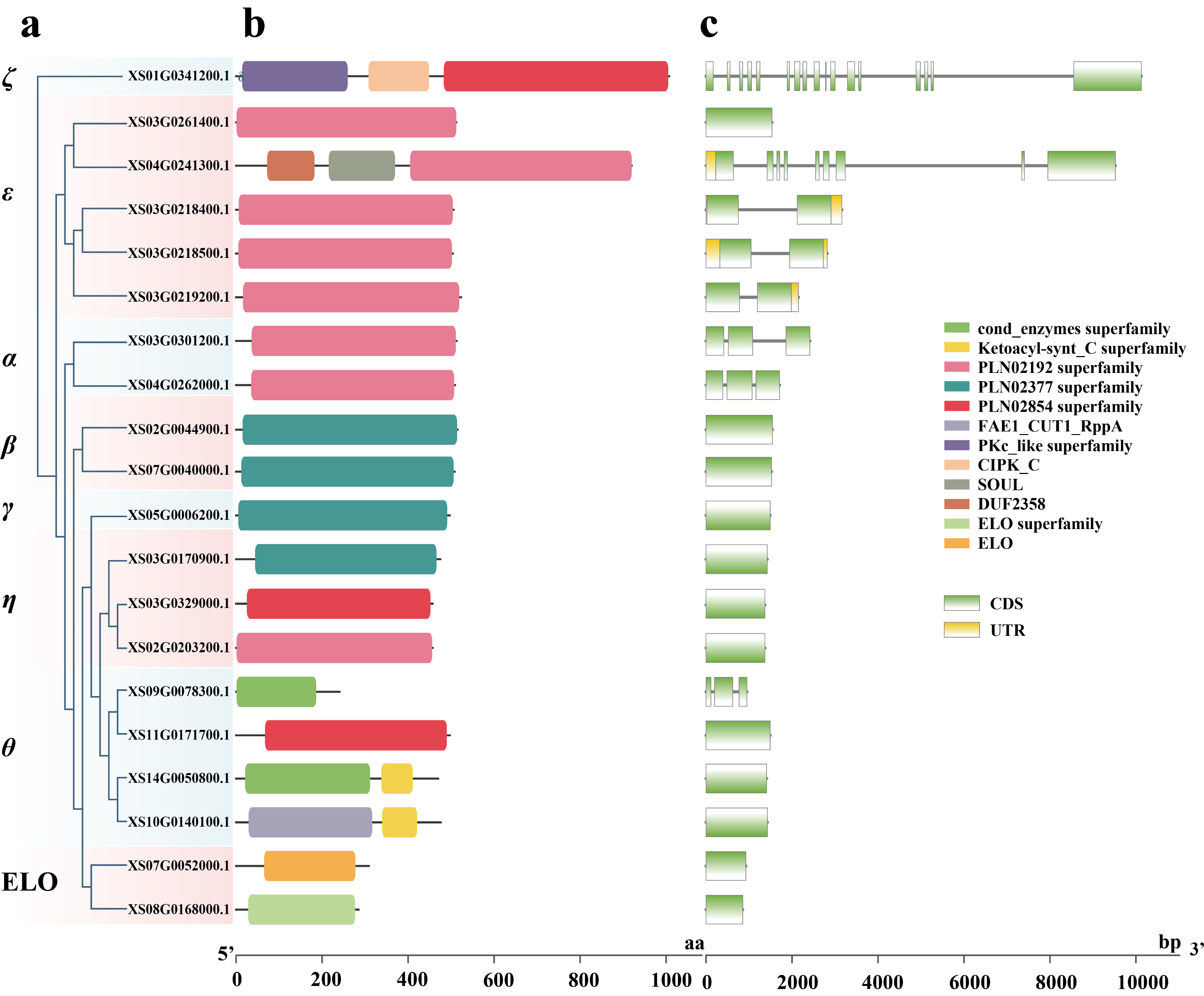
**

**Supplementary Figure 26. The phylogenetic tree, conserved domain, and gene structure of the candidate KCS genes and ELO-like genes.** (a) The maximum likelihood phylogenetic tree of the candidate KCS and ELO-like genes. (b) Conserved domains of the candidate KCS and ELO protein sequences annotated using CDD (Conserved Domain Database) (https://www.ncbi.nlm.nih.gov/cdd/). The conserved domains are indicated with boxes. (c) Gene structure analysis of the candidate KCS genes and ELO genes. The CDS, UTR, and introns are indicated with green filled boxes, yellow filled boxes, and grey lines, respectively.

**
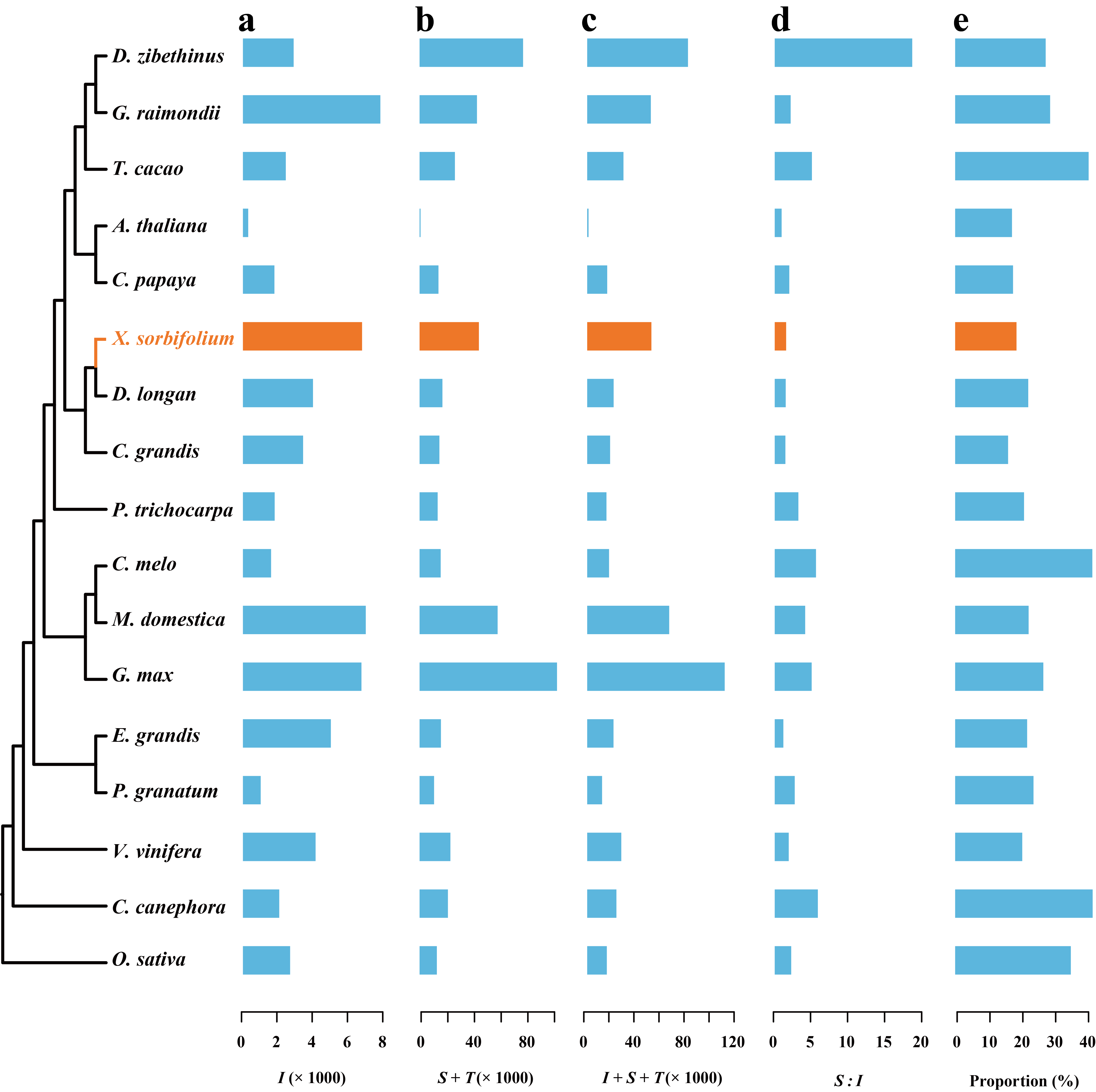
**

**Supplementary Figure 27. Birth and death of LTR-RTs in yellowhorn and 16 other plant genomes.** (a) Total number of intact LTR-RTs in yellowhorn (orange) and 16 plant genomes (*I*, intact LTR-RTs). (b) Total numbers of *S*+*T* values (*S*, solo-LTRs; *T*, truncated LTR-RTs). (c) Total numbers of intact LTR-RTs, solo-LTRs, and truncated LTR-RTs. (d) Ratio of solo-LTR to intact LTR-RT. (e) The proportions of the LTR-RT clusters with high removal rates (*S*:*I* ≥ 3).


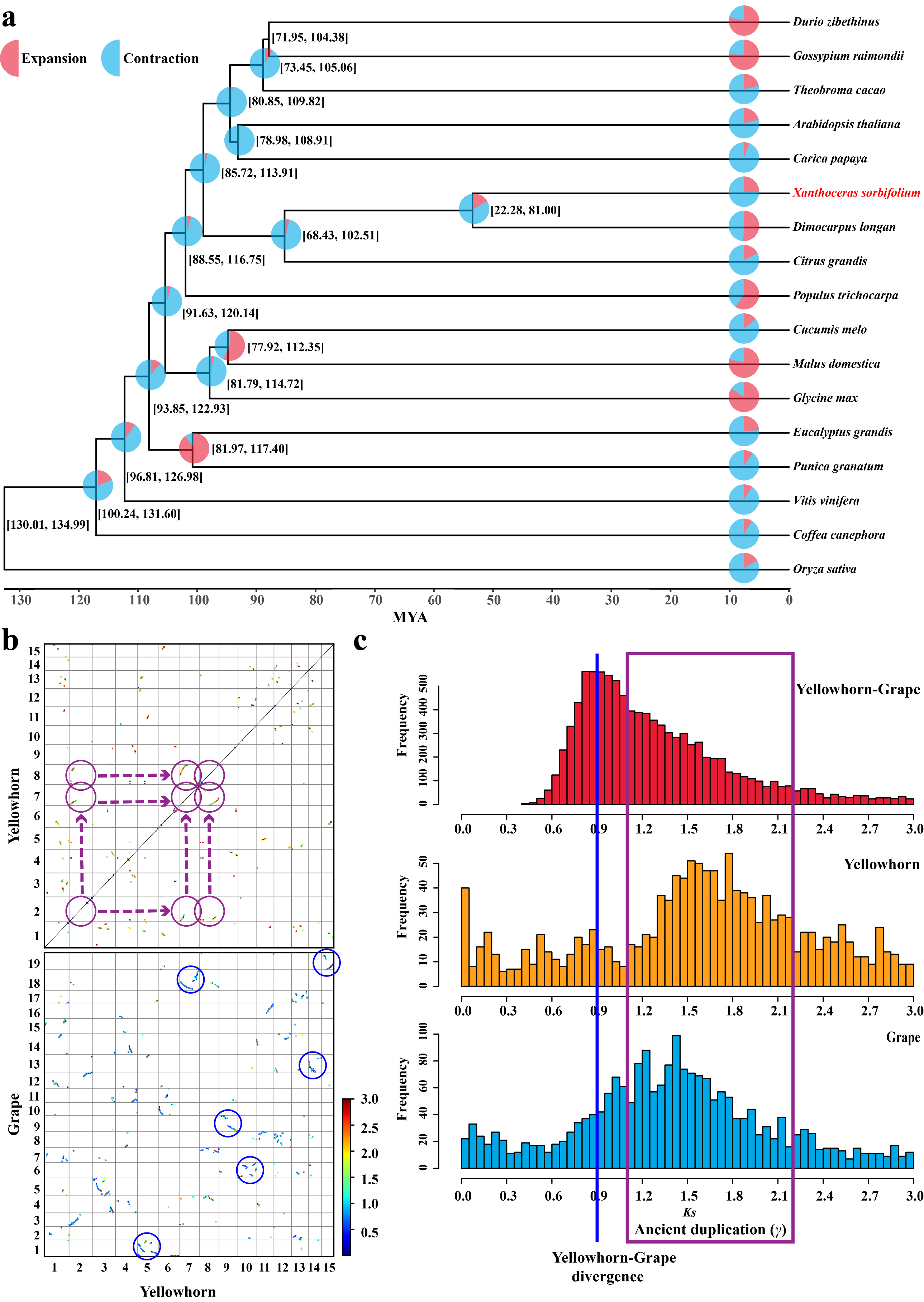


**Supplementary Figure 28.** **Phylogenetic and evolution analysis of yellowhorn genome**. (a) Phylogenetic tree, divergence time, and estimation of gene family expansion (red) and contraction (blue) on each evolutionary branch. The 95% confidence intervals for each dating point were presented in the brackets. (b) Dot plots of collinear blocks within yellowhorn, and between yellowhorn and grape. The color of dots represents the synonymous substitution (*Ks*) rate. The purple and blue circles represent the ancient gamma (*γ*) hexaploidy event and the 1:1 collinear block between yellowhorn and grape, respectively. (c) Synonymous substitution (*Ks*) distribution histogram of syntenic blocks between yellowhorn and grape, within yellowhorn, and within grape. The divergence between yellowhorn and grape and the ancient gamma (*γ*) hexaploidy event were presented with a vertical blue line and a purple rectangle, respectively.


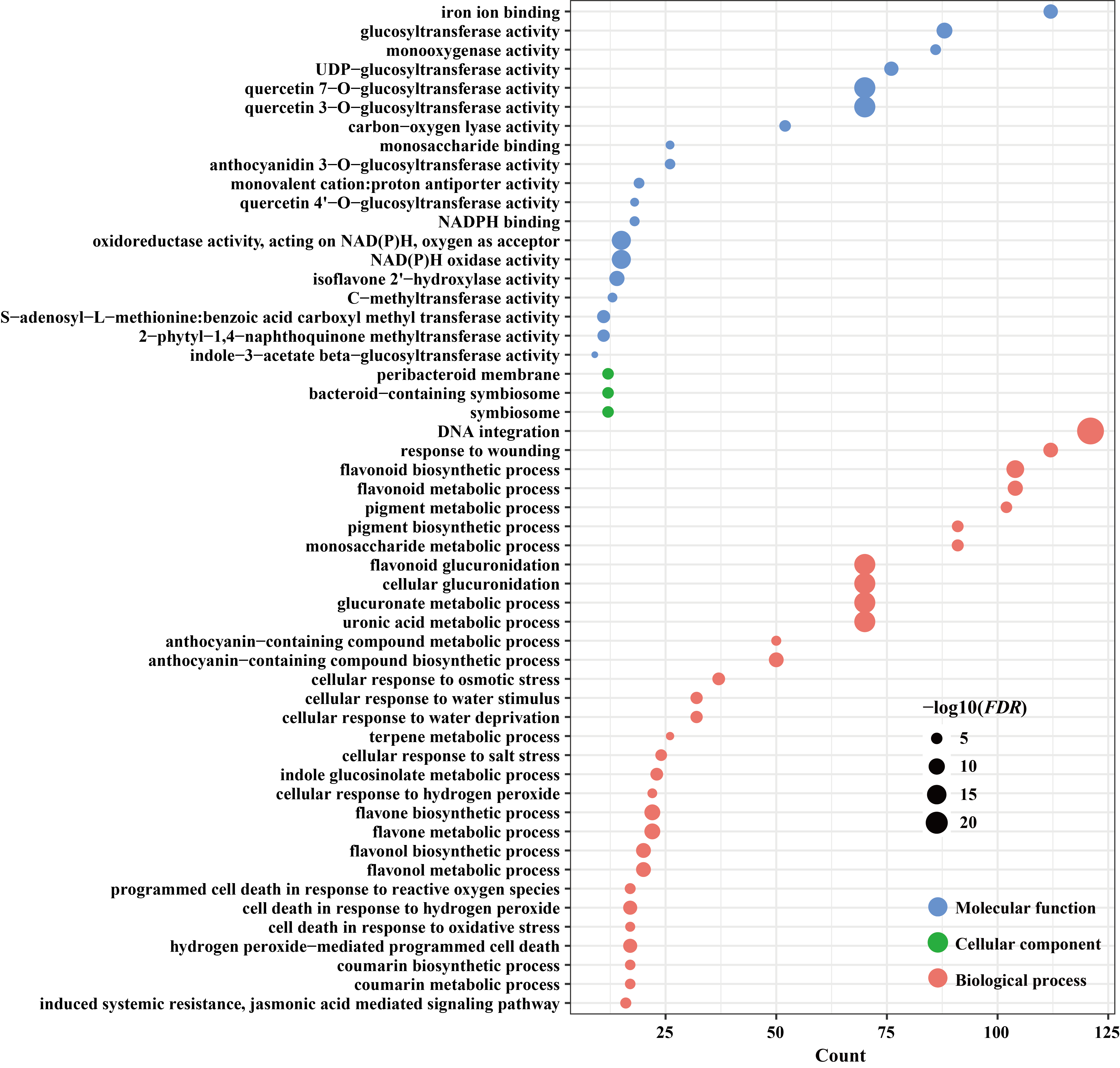


**Supplementary Figure 29. Gene Ontologies enrichment analysis of genes in expanded gene families.** The enriched GO terms with *FDR* < 0.001 were showed.

**Supplementary Table 1** Summary of the Illumina paired-end reads and Hi-C reads for the genome assembly of yellowhorn.

| **Platform** | **Library** | **Reads (Mb)** | **Bases (Gb)** | **Q20 (Gb)** | **Q30 (Gb)** |
| --- | --- | --- | --- | --- | --- |
| Illumina | 300_1 | 35.586 | 5.338 | 5.013 (93.9%) | 4.633 (86.8%) |
|  | 300_2 | 36.149 | 5.422 | 5.094 (94.0%) | 4.701 (86.7%) |
|  | 300_3 | 35.401 | 5.31 | 4.989 (94.0%) | 4.611 (86.8%) |
|  | 300_4 | 32.276 | 4.841 | 4.498 (92.9%) | 4.121 (85.1%) |
| Hi-C | G412 | 425.582 | 63.686 | 59.318 (93.1%) | 54.760 (86.0%) |

**Supplementary Table 2** Summary of the PacBio long-read data for the assembly of yellowhorn genome.

| **Platform** | **Reads (Mb)** | **Bases (Gb)** | **N50 (bp)** | **Average (bp)** | **Coverage** |
| --- | --- | --- | --- | --- | --- |
| PacBio | 8.456 | 63.146 | 10,577 | 7,467 | 120 |

**Supplementary Table 3** Summary statistics of different versions of the assembled genomes using different pipelines.

| **Version** | **Pipeline** | **Assembled size (Mb)** | **Contig/Scaffold number** | **Contig/Scaffold N50** | **BUSCO (%)** |
| --- | --- | --- | --- | --- | --- |
| v0.1 | CANU+SMARTdenovo | 482 | 1,607 | 779 Kb | 95.40 |
| v1.2 | v0.4+arrow+sspace+arrow+pilon×3 | 482 | 1,454/975 | 868 Kb/1.18 Mb | 95.00 |
| v2.1 | v1.2+Hi-C | 483 | 4,597/2000 | 388 Kb/29.7 Mb | 94.80 |
| v2.2 | v2.1+arrow×3+pilon×3 | 483 | 4,487/2002 | 406 Kb/29.7 Mb | 95.00 |
| final | v2.2+remove redundancy | 470 | 3,302/988 | 420 Kb/30.8 Mb | 94.50 |

**Supplementary Table 4** Summary of homolog chromosomes for the three yellowhorn genomes of “JGXP”, “WF18”, and “ZS4”. The chromosomes of the “JGXP” assembly are used as reference in the first column.

| **JGXP** | | | | |  | **ZS4** | |  | **WF18** | |
| --- | --- | --- | --- | --- | --- | --- | --- | --- | --- | --- |
| **Chromosome** | **Length (bp)** | | **Anchored contigs number** | **Gene number** |  | **Homolog chromosome** | **Gene number** |  | **Homolog chromosome** | **Gene number** |
| Chr01 | | 38,121,007 | 179 | 2,544 |  | LG1 | 2,949 |  | chr1 | 2,842 |
| Chr02 | | 36,665,449 | 230 | 1,366 |  | LG2 | 1,468 |  | chr3 | 1,480 |
| Chr03 | | 35,097,829 | 189 | 2,050 |  | LG3 | 2,170 |  | chr2 | 2,245 |
| Chr04 | | 34,138,836 | 193 | 1,488 |  | LG6 | 1,633 |  | chr4 | 1,701 |
| Chr05 | | 32,537,057 | 145 | 1,428 |  | LG8 | 1,510 |  | chr6 | 1,586 |
| Chr06 | | 32,286,479 | 165 | 1,337 |  | LG5 | 1,449 |  | chr12 | 1,046 |
| Chr07 | | 30,796,704 | 144 | 1,495 |  | LG4 | 1,735 |  | chr5 | 1,715 |
| Chr08 | | 29,682,413 | 162 | 1,267 |  | LG10 | 1,298 |  | chr7 | 1,509 |
| Chr09 | | 29,019,099 | 183 | 1,046 |  | LG9 | 1,073 |  | chr8 | 1,213 |
| Chr10 | | 28,176,291 | 159 | 1,046 |  | LG7 | 1,166 |  | chr9 | 1,250 |
| Chr11 | | 27,482,075 | 94 | 1,059 |  | LG12 | 1,140 |  | chr10 | 1,299 |
| Chr12 | | 26,813,003 | 134 | 1,350 |  | LG13 | 1,614 |  | chr14 | 798 |
| Chr13 | | 26,671,535 | 98 | 1,550 |  | LG11 | 1,844 |  | chr13 | 1,221 |
| Chr14 | | 19,545,690 | 111 | 940 |  | LG15 | 1,097 |  | chr15 | 915 |
| Chr15 | | 19,200,632 | 37 | 890 |  | LG14 | 1,219 |  | chr11 | 1,195 |
| Total | | 446,234,099 | 2,223 | 20,856 |  | - | 23,365 |  | - | 22,015 |

**Supplementary Table 5** Mapping rates of PacBio long reads, Illumina paired-end reads, and transcriptome to the yellowhorn genome.

| **Type** | **Coverage (%)** | **Coverage ≥5**× (%) | **Coverage ≥10**× (%) | **Coverage ≥20**× (%) |
| --- | --- | --- | --- | --- |
| PacBio long reads | 99.39 | 98.76 | 98.04 | 95.29 |
| Illumina paired-end reads | 97.66 | NA | NA | NA |
| Transcriptome | 91.43 | NA | NA | NA |

NA: data not available.

**Supplementary Table 6** Summary statistics for the gene annotation of yellowhorn genome.

| **Genomic feature** | **Number** |
| --- | --- |
| Protein-coding gene | 22,049 |
| Transcript | 22,049 |
| Average gene length (bp) | 4,276.93 |
| Average transcript/CDS length (bp) | 1,651.83/1,325.68 |
| Average exons per transcript | 6.06 |
| Average exon/intron length (bp) | 272.79/520.01 |
| Complete BUSCOs covered by protein-coding gene | 93.1% |
| Fragment pseudogene | 11,197 |
| Duplicated pseudogene | 4,120 |
| Retro-transpose pseudogene | 1,069 |
| Pseudogene | 16,386 |
| small ncRNA | 588 |
| rRNA | 65 |
| tRNA | 708 |

**Supplementary Table 7** Summary for the functional annotation of protein-coding genes.

| **Class** | **Number** | **Percent (%)** |
| --- | --- | --- |
| Swiss-Prot | 13,865 | 62.9 |
| TrEMBL | 20,854 | 94.6 |
| Nr | 20,944 | 95.0 |
| Pfam | 18,357 | 83.3 |
| eggNOG | 20,147 | 91.4 |
| InterProScan | 21,743 | 98.6 |
| GO | 17,622 | 79.9 |
| KEGG | 9,002 | 40.8 |
| Annotated | 21,856 | 99.1 |
| Unannotated | 193 | 0.9 |

**Supplementary Table 8** Annotated gene families of transcription factors, transcriptional regulators, and chromatin regulators genes in yellowhorn genome.

| **Family** | **Numbers** | **Type** |
| --- | --- | --- |
| A20-like | 15 | putative novel transcription regulator |
| ABTB | 6 | chromatin remodeling & transcription regulator |
| AP2-EREBP | 72 | transcription factor |
| ARF | 17 | transcription factor |
| ARID | 4 | transcription factor & chromatin remodeling |
| ARID-HMG | 3 | transcription factor & chromatin remodeling |
| AS2-LOB | 37 | transcription factor |
| AUX-IAA | 23 | transcription factor interactor and regulator |
| B3-Domain | 49 | transcription factor |
| BED-type(Zn) | 34 | transcription factor / chromatin remodeling |
| BES/BZR | 7 | transcription factor |
| bHLH | 120 | transcription factor |
| Bromodomain | 21 | chromatin remodeler |
| BTB-POZ | 27 | chromatin remodeling & transcription regulator |
| BTB-POZ-MATH | 5 | chromatin remodeling & transcription regulator |
| bZIP | 52 | transcription factor |
| C2C2-CO-like | 25 | transcription factor |
| C2C2-Dof | 22 | transcription factor |
| C2C2-GATA | 23 | transcription factor |
| C2C2-YABBY | 7 | transcription factor |
| C2H2 | 457 | transcription factor |
| C3H | 65 | transcription factor |
| C3H-WRC/GRF | 18 | transcription factor interactor and regulator |
| CCHC(Zn) | 239 | transcription factor interactor and regulator |
| CG1-CAMTA | 3 | transcription factor |
| CHROMO-DOMAIN | 30 | chromatin remodeling & transcriptional activation |
| CW-Zn | 6 | putative transcription factor & chromatin remodeling |
| CW-Zn-B3/VAL | 3 | transcription factor & chromatin remodeling |
| DDT | 7 | transcription factor & chromatin remodeling |
| E2F-DP | 5 | transcription factor |
| EIL | 3 | transcription factor |
| FAR | 38 | transcription factor |
| FHA-SMAD | 19 | transcription factor interactor and regulator |
| FYR | 5 | chromatin remodeling & transcription regulator |
| GAGA-Binding-like | 4 | transcription factor |
| GARP-G2-like | 7 | transcription factor |
| GeBP | 4 | transcription factor |
| GRAS | 42 | transcription factor |
| Hap2/NF-YA | 7 | transcription factor |
| Hap3/NF-YB | 57 | transcription factor |
| HD-SAD | 13 | transcription factor & lipid binding |
| HD-ZIP | 15 | transcription factor |
| HMG | 14 | chromatin remodeling & transcriptional activation |
| Homeodomain-LIKE | 6 | transcription regulator |
| Homeodomain-PHD | 2 | chromatin remodeling & transcription regulator |
| Homeodomain-TALE-BEL | 11 | transcription factor |
| Homeodomain-TALE-KNOX | 7 | transcription factor |
| Homobox-WOX | 61 | transcription factor |
| HSA | 2 | chromatin remodeler |
| HSF-type-DNA-binding | 18 | transcription factor |
| ISWI | 1 | chromatin remodeling |
| JmjC | 18 | transcription factor & chromatin remodeling & Metalloenzymes |
| JmjC-ARID | 1 | transcription factor & chromatin remodeling |
| JmjN | 6 | transcription factor & chromatin remodeling |
| JUMONJI | 6 | transcription factor & chromatin remodeling |
| Lambda-DB | 3 | putative novel transcription factor |
| LFY | 1 | transcription factor |
| LIM | 19 | transcription factor interactor and regulator |
| LisH | 23 | transcription factor interactor and regulator |
| MADS-MIKC | 21 | transcription factor |
| MADS-type1 | 45 | transcription factor |
| MYB | 26 | transcription factor |
| MYB/SANT | 19 | transcription factor |
| MYB-HB-like | 166 | transcription factor |
| MYB-related | 1 | transcription factor |
| NAM | 120 | transcription factor |
| Nin-like | 9 | transcription factor |
| NOZZLE | 2 | transcription factor |
| PAZ-Argonaute | 13 | post-transcriptional gene silencing |
| PHD | 129 | chromatin regulator |
| PLATZ | 6 | putative novel transcription repressor |
| RAV | 3 | transcription factor |
| RB | 2 | putative novel transcription repressor |
| RR-A-type | 32 | response regulator and putative novel transcription factor |
| RR-B-type | 4 | response regulator and putative novel transcription factor |
| S1Fa-like | 1 | transcription factor |
| SAP | 8 | transcription regulator |
| SBP | 14 | transcription factor |
| SET | 34 | chromatin remodeling |
| SNF2 | 30 | chromatin remodeling |
| ssDNA-binding-TF | 5 | transcription factor |
| SSXT | 2 | putative novel transcription factor |
| STY-LRP1 | 4 | transcription factor |
| SWIB-Plus-3 | 4 | chromatin remodeling |
| TCP | 21 | transcription factor |
| Tc-PD | 2 | transcription coactivator |
| Tesmin | 6 | putative novel transcription factor |
| TIFY | 14 | transcription factor |
| TTF-type(Zn) | 1 | transcription factor and/or regulators |
| TUBBY | 9 | transcription factor |
| WD40-like | 236 | transcription factor |
| WRKY | 42 | transcription factor |
| YEATS | 2 | putative novel transcription factor |
| ZF-HD | 11 | transcription factor |
| Znf-B | 21 | transcription factor interactor and regulator |
| Znf-LSD | 7 | transcription factor |

**Supplementary Table 9** Statistics of repeat elements for the three yellowhorn genomes of “JGXP”, “WF18”, and “ZS4”.

|  | **JGXP** | | |  | **ZS4** | | |  | **WF18** | | |  |
| --- | --- | --- | --- | --- | --- | --- | --- | --- | --- | --- | --- | --- |
| **Class** | **Number** | **Length (bp)** | **Percent (%)** |  | **Number** | **Length (bp)** | **Percent (%)** |  | **Number** | **Length (bp)** | **Percent (%)** | |
| LTR | 166,495 | 139,290,646 | 29.64 |  | 189,593 | 158,233,075 | 31.37 |  | 160,560 | 129,635,817 | 29.46 | |
| LTR/Cassandra | 206 | 26,652 | 0.01 |  | 0 | 0 | 0 |  | 0 | 0 | 0 | |
| LTR/Caulimovirus | 3,047 | 3,107,304 | 0.66 |  | 7,823 | 4,439,876 | 0.88 |  | 2,034 | 1,796,675 | 0.41 | |
| LTR/Copia | 66,858 | 55,825,097 | 11.88 |  | 76,264 | 63,496,438 | 12.59 |  | 64,175 | 52,145,918 | 11.85 | |
| LTR/DIRS | 433 | 65,663 | 0.01 |  | 0 | 0 | 0 |  | 0 | 0 | 0 | |
| LTR/ERV1 | 454 | 222,953 | 0.05 |  | 243 | 97,225 | 0.02 |  | 0 | 0 | 0 | |
| LTR/ERVK | 0 | 0 | 0 |  | 255 | 111,497 | 0.02 |  | 0 | 0 | 0 | |
| LTR/Gypsy | 92,198 | 79,104,389 | 16.83 |  | 102,305 | 89,661,383 | 17.78 |  | 91,560 | 73,972,004 | 16.81 | |
| LTR/Pao | 0 | 0 | 0 |  | 110 | 45,224 | 0.01 |  | 0 | 0 | 0 | |
| LTR/Ngaro | 2,664 | 840,543 | 0.18 |  | 0 | 0 | 0 |  | 139 | 206,879 | 0.05 | |
| LINE | 28,507 | 19,097,970 | 4.06 |  | 33,537 | 26,153,809 | 5.19 |  | 21,291 | 18,559,104 | 4.22 | |
| LINE/I-Jockey | 0 | 0 | 0 |  | 575 | 273,094 | 0.05 |  | 0 | 0 | 0 | |
| LINE/LINE1 | 23,589 | 17,804,744 | 3.79 |  | 23,691 | 22,118,159 | 4.39 |  | 18,538 | 17,952,441 | 4.08 | |
| LINE/LINE1-Tx1 | 2,563 | 608,507 | 0.13 |  | 0 | 0 | 0 |  | 0 | 0 | 0 | |
| LINE/LINE2 | 289 | 82,094 | 0.02 |  | 900 | 207,797 | 0.04 |  | 0 | 0 | 0 | |
| LINE/Penelope | 327 | 99,075 | 0.02 |  | 0 | 0 | 0 |  | 0 | 0 | 0 | |
| LINE/Tad1 | 1,739 | 503,550 | 0.11 |  | 8,371 | 3,554,759 | 0.70 |  | 2,753 | 606,663 | 0.14 | |
| SINE | 2,287 | 433,426 | 0.09 |  | 417 | 59,697 | 0.01 |  | 1,055 | 174,806 | 0.04 | |
| SINE/tRNA | 581 | 72,028 | 0.02 |  | 282 | 39,655 | 0.01 |  | 329 | 71,465 | 0.02 | |
| SINE/tRNA-Meta | 0 | 0 | 0 |  | 0 | 0 | 0 |  | 328 | 41,636 | 0.01 | |
| DNA | 67,892 | 26,418,792 | 5.62 |  | 80,782 | 32,005,058 | 6.35 |  | 79,360 | 29,380,132 | 6.68 | |
| DNA/CMC-EnSpm | 5,017 | 3,195,799 | 0.68 |  | 6,098 | 2,608,913 | 0.52 |  | 5,245 | 2,179,298 | 0.50 | |
| DNA/Ginger | 0 | 0 | 0 |  | 86 | 44,762 | 0.01 |  | 0 | 0 | 0 | |
| DNA/MULE-MuDR | 2,293 | 595,293 | 0.13 |  | 11,665 | 5,235,612 | 1.04 |  | 5,989 | 1,737,046 | 0.39 | |
| DNA/Maverick | 263 | 109,304 | 0.02 |  | 461 | 58,622 | 0.01 |  | 1,238 | 516,687 | 0.12 | |
| DNA/MuLE-MuDR | 5,293 | 4,107,298 | 0.87 |  | 6,195 | 4,239,675 | 0.84 |  | 4,753 | 2,963,326 | 0.67 | |
| DNA/PIF-Harbinger | 15,309 | 4,524,777 | 0.96 |  | 13,605 | 4,227,731 | 0.84 |  | 13,259 | 4,458,497 | 1.01 | |
| DNA/Sola-3 | 0 | 0 | 0 |  | 138 | 32,893 | 0.01 |  | 0 | 0 | 0 | |
| DNA/PiggyBac | 247 | 66,931 | 0.01 |  | 0 | 0 | 0 |  | 0 | 0 | 0 | |
| DNA/TcMar-Mariner | 0 | 0 | 0 |  | 0 | 0 | 0 |  | 275 | 171,368 | 0.04 | |
| DNA/TcMar-Pogo | 752 | 252,481 | 0.05 |  | 1,139 | 340,787 | 0.07 |  | 1,788 | 397,494 | 0.09 | |
| DNA/TcMar-Tc4 | 0 | 0 | 0 |  | 0 | 0 | 0 |  | 786 | 238,130 | 0.05 | |
| DNA/Zisupton | 449 | 116,065 | 0.02 |  | 237 | 51,481 | 0.01 |  | 2,095 | 616,049 | 0.14 | |
| DNA/hAT-Ac | 14,779 | 5,908,149 | 1.26 |  | 21,190 | 8,742,512 | 1.73 |  | 18,115 | 8,436,766 | 1.92 | |
| DNA/hAT-Charlie | 5,241 | 1,240,697 | 0.26 |  | 0 | 0 | 0 |  | 8,899 | 2,650,556 | 0.60 | |
| DNA/hAT-Tag1 | 5,832 | 2,020,140 | 0.43 |  | 4,832 | 1,837,699 | 0.36 |  | 7,257 | 2,087,050 | 0.47 | |
| DNA/hAT-Tip100 | 4,316 | 1,747,515 | 0.37 |  | 6,551 | 2,284,225 | 0.45 |  | 5,088 | 1,634,186 | 0.37 | |
| RC | 3,429 | 1,522,944 | 0.32 |  | 4,641 | 2,095,730 | 0.42 |  | 1,072 | 475,199 | 0.11 | |
| RC/Helitron | 3,429 | 1,522,944 | 0.32 |  | 4,641 | 2,095,730 | 0.42 |  | 1,072 | 475,199 | 0.11 | |
| Unknown | 300,763 | 114,080,231 | 24.27 |  | 302,741 | 99,384,074 | 19.70 |  | 241,351 | 84,140,969 | 19.12 | |
| rRNA | 0 | 0 | 0 |  | 261 | 348,068 | 0.07 |  | 0 | 0 | 0 | |
| Simple repeat | 152,297 | 6,550,426 | 1.39 |  | 176,440 | 8,121,632 | 1.61 |  | 139,072 | 7,179,020 | 1.63 | |
| Low complexity | 24,605 | 1,241,980 | 0.26 |  | 27,479 | 1,423,884 | 0.28 |  | 21,700 | 1,124,528 | 0.26 | |
| Total | 746,275 | 308,636,415 | 65.67 |  | 815,891 | 327,825,027 | 65.00 |  | 665,461 | 270,669,575 | 61.52 | |

**Note:** We re-annotated the repeat elements of the previously published genome assembly using the same method with the present study.

**Supplementary Table 10** Summary of the JGXP-specific TEs verified by the completely coverage of PacBio long-reads.

| **Class** | **Number of TE** | **“JGXP” PacBio reads** | |  | **“ZS4” PacBio reads** | |
| --- | --- | --- | --- | --- | --- | --- |
|  |  | **Number of verified TE** | **Depth** |  | **Number of verified TE** | **Depth** |
| LTR/Cassandra | 206 | 206 | 205 |  | 202 | 123 |
| LTR/DIRS | 433 | 432 | 99 |  | 425 | 115 |
| LTR/Ngaro | 2,664 | 2,663 | 89 |  | 2611 | 107 |
| LINE/LINE1-Tx1 | 2,563 | 2,563 | 94 |  | 2524 | 110 |
| LINE/Penelope | 327 | 327 | 86 |  | 320 | 101 |
| DNA/PiggyBac | 247 | 247 | 91 |  | 236 | 106 |

**Supplementary Table 11** Summary of identified structural variation among the three yellowhorn genomes of “JGXP”, “WF18”, and “ZS4”. The genome of “JGXP” as reference genome. SNP, single nucleotide polymorphism.

| **Type** | **JGXP *vs*. ZS4** | |  | **JGXP *vs*. WF18** | |
| --- | --- | --- | --- | --- | --- |
|  | **Number** | **Size (Mb)** |  | **Number** | **Size (Mb)** |
| Copy-gain | 376 | 1.5 |  | 273 | 0.8 |
| Copy-loss | 704 | 6.5 |  | 500 | 5.6 |
| Deletion | 175,557 | 6.8 |  | 148,246 | 6.1 |
| Duplicated regions gain | 3,209 | 18.8 |  | 2,600 | 12.8 |
| Duplicated regions loss | 366 | 5.6 |  | 301 | 4.7 |
| Highly diverged regions | 2,080 | 33.6 |  | 1,702 | 30.5 |
| Insertion | 833,355 | 0.8 |  | 613,751 | 0.6 |
| Inverted-duplicated regions gain | 2,673 | 13.5 |  | 2,121 | 8.0 |
| Inverted-duplicated regions loss | 344 | 4.2 |  | 272 | 2.3 |
| Inverted-translocated regions | 1,255 | 16.8 |  | 1,139 | 11.2 |
| Inverted regions | 378 | 56.9 |  | 426 | 51.4 |
| WF18/ZS4-specific regions | 8,838 | 118.5 |  | 7,349 | 91.0 |
| JGXP-specific regions | 5,484 | 119.9 |  | 4,904 | 129.7 |
| SNP | 1,974,879 | 2.0 |  | 1,567,424 | 1.6 |
| Syntenic regions | 3,652 | 241.5 |  | 3,027 | 242.2 |
| Tandem repeat | 56 | 0.4 |  | 42 | 0.3 |
| Translocated regions | 1,496 | 22.4 |  | 1,221 | 16.6 |

**Supplementary Table 12** The number, size, and the length summary of gene, LINE1, *Gypsy*, and *Copia* in the centromeric (Y) and non-centromeric (N) regions. SD, Standard deviation.

| **Element** | **Centromere** | **Number** | **Szie (Mb)** | **Length (bp)** | | |
| --- | --- | --- | --- | --- | --- | --- |
|  |  |  |  | **Average** | **Median** | **SD** |
| gene | Y | 287 | 1.5 | 5,199 | 3,757 | 5,092 |
| gene | N | 20,569 | 88.0 | 4,280 | 3,087 | 4,198 |
| LINE1 | Y | 3,312 | 4.2 | 1,269 | 492 | 1,639 |
| LINE1 | N | 18,883 | 12.6 | 669 | 327 | 965 |
| Intact LINE1 | Y | 61 | 0.5 | 7,891 | 7,784 | 389 |
| Intact LINE1 | N | 114 | 0.9 | 7,457 | 7,626 | 805 |
| *Gypsy* | Y | 6,592 | 6.2 | 945 | 488 | 1,313 |
| *Gypsy* | N | 80,233 | 68.4 | 852 | 460 | 1,217 |
| Intact *Gypsy* | Y | 226 | 2.0 | 8,855 | 7,267 | 2,712 |
| Intact *Gypsy* | N | 2,554 | 23.7 | 9,282 | 9,143 | 2,634 |
| *Copia* | Y | 3,567 | 3.6 | 1,018 | 604 | 1,194 |
| *Copia* | N | 60,004 | 49.7 | 828 | 450 | 1,022 |
| Intact *Copia* | Y | 87 | 0.6 | 6,739 | 5,540 | 2,172 |
| Intact *Copia* | N | 3,618 | 23.0 | 6,363 | 5,386 | 2,100 |

**Supplementary Table 13** The insertion time of LINE1, intact *Copia* and intact *Gypsy* in the centromeric (Y) and non-centromeric (N) regions. The insertion time of LINE1 elements were calculated by comparing with their best BLAST hit. The insertion time of intact *Gypsy* and intact *Copia* elements were calculated by comparing the 5’-LTR and 3’-LTR. MYA means million years ago; SD, Standard deviation.

| **Element** | **Centromere** | **Number** | **Insertion time (MYA)** | | |
| --- | --- | --- | --- | --- | --- |
|  |  |  | **Average** | **Median** | **SD** |
| LINE1 | Y | 623 (pair) | 1.17 | 0.67 | 1.41 |
| LINE1 | N | 3,774 (pair) | 4.42 | 3.01 | 5.61 |
| *Gypsy* | Y | 226 | 1.03 | 0.66 | 1.00 |
| *Gypsy* | N | 2,554 | 1.26 | 0.95 | 1.20 |
| *Copia* | Y | 87 | 1.64 | 1.34 | 1.37 |
| *Copia* | N | 3,618 | 1.42 | 0.98 | 1.39 |

**Supplementary Table 14** Summary of yellowhorn and the other 16 species used for phylogenetic, gene family, and LTR-RTs evolution analysis.

| **Species** | **Clade** | **Genome version** | **Genome size (Mb)** | **Scaffold N50 (Mb)** | **Gene number** | **Reference** |
| --- | --- | --- | --- | --- | --- | --- |
| *Xanthoceras sorbifolium* | rosids | v2f | 469 | 30.8 | 22,049 | This study |
| *Dimocarpus longan* | rosids | - | 495 | 0.6 | 39,282 | (Lin et al., 2017) |
| *Citrus grandis* | rosids | version1 | 346 | 40.4 | 30,123 | (Wang et al., 2017) |
| *Durio zibethinus* | rosids | Duzib1.0 | 715 | 22.7 | 35,832 | (Teh et al., 2017) |
| *Theobroma cacao* | rosids | v1.1 | 346 | 34.0 | 29,452 | *(Argout et al., 2011)* |
| *Gossypium raimondii* | rosids | v2.1 | 761.4 | 62.2 | 37,505 | (Wang et al., 2012) |
| *Punica granatum* | rosids | ASM220158v1 | 296 | 2.3 | 29,127 | (Yuan et al., 2018) |
| *Eucalyptus grandis* | rosids | v2.0 | 691 | 58.0 | 36,349 | (Myburg et al., 2014) |
| *Carica papaya* | rosids | Payaya1.0 | 370 | 1.1 | 18,003 | (Ming et al., 2008) |
| *Arabidopsis thaliana* | rosids | TAIR10 | 135 | 22.4 | 27,416 | (Swarbreck et al., 2007) |
| *Populus trichocarpa* | rosids | v3.1 | 423 | 20.0 | 41,335 | (Tuskan et al., 2006) |
| *Glycine max* | rosids | v1.0 | 978 | 49.0 | 56,044 | (Schmutz et al., 2010) |
| *Malus domestica* | rosids | GDDH13 v1.1 | 710 | 37.6 | 45,116 | (Daccord et al., 2017) |
| *Cucumis melo* | rosids | v3.5.1 | 407 | 31.4 | 27,485 | (Garcia-Mas et al., 2012) |
| *Vitis vinifera* | rosids | Genoscope.12X | 486 | 23.0 | 26,346 | (The French–Italian Public Consortium for Grapevine Genome et al., 2007) |
| *Coffea canephora* | asterids | - | 569 | 38.2 | 25,574 | (Denoeud et al., 2014), |
| *Oryza sativa* | commelinids | MSU_v7.0 | 372 | 28.6 | 42,189 | (Ouyang et al., 2006) |

**Supplementary Table 15** Summary of original and filtered intact LTR-RT, solo-LTR, and truncated LTR-RT. *I,* Intact LTR-RTs; *S*, Solo-LTRs; *T*, Truncated LTR-RTs.

| Species | *I* | Cluster number | *S* | *T* | *S*+*T* | *I*+*S*+*T* | Filtered | | | | | | |  |
| --- | --- | --- | --- | --- | --- | --- | --- | --- | --- | --- | --- | --- | --- | --- |
|  |  |  |  |  |  |  | Scaffold length (kb) | *I* | *S* | *T* | *S*/*I* | *T*/*I* | (*S*+*T*/*I*) | Cluster *S*/*I*≥3 (%) |
| *Xanthoceras sorbifolium* | 6,749 | 1,537 | 10,771 | 33,692 | 44,463 | 51,212 | 30 | 6,621 | 10,155 | 32,943 | 1.53 | 4.98 | 6.51 | 18.66 |
| *Durio zibethinus* | 2,871 | 1,067 | 53,594 | 23,978 | 77,572 | 80,443 | 255 | 2,761 | 51,220 | 22,111 | 18.55 | 8.01 | 26.56 | 27.55 |
| *Gossypium raimondii* | 7,786 | 3,285 | 16,696 | 26,306 | 43,002 | 50,788 | 0 | 7,786 | 16,696 | 26,306 | 2.14 | 3.38 | 5.52 | 28.90 |
| *Theobroma cacao* | 2,427 | 781 | 12,280 | 14,204 | 26,484 | 28,911 | 50 | 2,415 | 12,111 | 14,149 | 5.01 | 5.86 | 10.87 | 40.62 |
| *Arabidopsis thaliana* | 299 | 205 | 275 | 450 | 725 | 1,024 | 0 | 299 | 275 | 450 | 0.92 | 1.51 | 2.42 | 17.28 |
| *Carica papaya* | 1,782 | 889 | 7,909 | 6,239 | 14,148 | 15,930 | 60 | 1,433 | 2,810 | 4,511 | 1.96 | 3.15 | 5.11 | 17.61 |
| *Dimocarpus longan* | 3,969 | 1,257 | 6,268 | 10,854 | 17,122 | 21,091 | 125 | 3,718 | 5,473 | 10,128 | 1.47 | 2.72 | 4.20 | 22.24 |
| *Citrus grandis* | 3,407 | 787 | 4,876 | 9,987 | 14,863 | 18,270 | 0 | 3,407 | 4,876 | 9,987 | 1.43 | 2.93 | 4.36 | 16.11 |
| *Populus trichocarpa* | 1,797 | 806 | 6,249 | 7,300 | 13,549 | 15,346 | 215 | 1,684 | 5,361 | 6,763 | 3.18 | 4.02 | 7.20 | 20.95 |
| *Cucumis melo* | 1,596 | 757 | 8,869 | 6,933 | 15,802 | 17,398 | 0 | 1,596 | 8,869 | 6,933 | 5.56 | 4.34 | 9.90 | 41.75 |
| *M. domestica* | 6,959 | 1,855 | 28,543 | 29,957 | 58,500 | 65,459 | 0 | 6,959 | 28,543 | 29,957 | 4.10 | 4.30 | 8.41 | 22.35 |
| *Glycine max* | 6,714 | 1,585 | 34,605 | 68,430 | 103,035 | 109,749 | 755 | 6,643 | 33,113 | 67,592 | 4.98 | 10.17 | 15.16 | 26.84 |
| *Eucalyptus grandis* | 4,979 | 1,317 | 6,624 | 9,379 | 16,003 | 20,982 | 70 | 4,741 | 5,520 | 8,763 | 1.16 | 1.85 | 3.01 | 21.91 |
| *Punica granatum* | 1,005 | 370 | 5,778 | 5,058 | 10,836 | 11,841 | 1,150 | 633 | 1,700 | 1,623 | 2.69 | 2.56 | 5.25 | 23.84 |
| *Vitis vinifera* | 4,116 | 915 | 7,734 | 15,339 | 23,073 | 27,189 | 0 | 4,116 | 7,734 | 15,339 | 1.88 | 3.73 | 5.61 | 20.41 |
| *Coffea canephora* | 2,055 | 765 | 11,938 | 9,291 | 21,229 | 23,284 | 0 | 2,055 | 11,938 | 9,291 | 5.81 | 4.52 | 10.33 | 41.85 |
| *Oryza sativa* | 2,672 | 489 | 5,936 | 7,029 | 12,965 | 15,637 | 0 | 2,672 | 5,936 | 7,029 | 2.22 | 2.63 | 4.85 | 35.22 |

**Supplementary Table 16** Summary of the Illumina RNA-sequencing paired-end reads used for the gene annotation of yellowhorn genome.

| Sample | Tissue | Raw reads (Mb) | Clean reads (Mb) | Mapping rate |
| --- | --- | --- | --- | --- |
| A0-10Y | fruit | 44.323 | 40.684 | 0.87 |
| A0-2G | fruit | 43.089 | 40.015 | 0.95 |
| A0-3G | fruit | 41.372 | 38.498 | 0.95 |
| A0-4GR | fruit | 44.783 | 41.528 | 0.94 |
| A0-4Y | fruit | 44.319 | 39.752 | 0.94 |
| A0-5GP | fruit | 47.170 | 43.661 | 0.96 |
| A0-5GR | fruit | 42.041 | 38.420 | 0.96 |
| A0-5Y | fruit | 42.232 | 38.771 | 0.94 |
| A0-6Y | fruit | 43.686 | 40.562 | 0.95 |
| A0-7Y | fruit | 46.579 | 43.612 | 0.94 |
| A0-8Y | fruit | 44.324 | 40.074 | 0.93 |
| A0-H1-CH | flower | 33.179 | 29.351 | 0.95 |
| A0-H1-HX | flower | 31.022 | 27.121 | 0.95 |
| A0-H1-SK | flower | 40.504 | 35.353 | 0.96 |
| A0-H2-XH-BH | flower | 56.684 | 53.298 | 0.96 |
| A0-H2-XH-HU | flower | 40.590 | 37.665 | 0.96 |
| A0-H2-XH-QH | flower | 51.054 | 48.385 | 0.96 |
| A0-H3-LXH-LH | flower | 45.569 | 42.388 | 0.96 |
| A0-H3-XH-LH | flower | 47.841 | 44.922 | 0.96 |
| A0-Y1 | flower | 53.484 | 50.767 | 0.96 |
| A0-Y3 | flower | 42.096 | 39.207 | 0.96 |
| B-DHX | flower | 29.378 | 26.743 | 0.95 |
| C9-1G | fruit | 48.093 | 44.573 | 0.95 |
| C9-2G | fruit | 43.399 | 40.112 | 0.97 |
| C9-4GP | fruit | 51.452 | 47.501 | 0.95 |
| C9-4GR | fruit | 42.150 | 39.086 | 0.95 |
| C9-4Y | fruit | 45.233 | 41.576 | 0.95 |
| C9-5GP | fruit | 44.545 | 41.046 | 0.96 |
| C9-5Y | fruit | 40.847 | 37.616 | 0.95 |
| C9-6Y | fruit | 44.322 | 40.736 | 0.93 |
| C9-7Y | fruit | 35.591 | 32.562 | 0.95 |
| C9-8Y | fruit | 46.191 | 42.556 | 0.95 |
| C9-H1-CH | flower | 53.838 | 50.595 | 0.96 |
| C9-H1-CX | flower | 51.791 | 48.500 | 0.97 |
| C9-H1-DX | flower | 45.721 | 42.785 | 0.97 |
| C9-H1-HX | flower | 47.598 | 43.895 | 0.97 |
| C9-H1-XH | flower | 57.083 | 53.363 | 0.96 |
| C9-H2-BH-LXH | flower | 59.365 | 56.085 | 0.96 |
| C9-H2-LXH-QH | flower | 45.697 | 42.505 | 0.97 |
| C9-H2-QH-XH | flower | 59.670 | 55.709 | 0.96 |
| C9-H3-XH-LH | flower | 58.242 | 54.316 | 0.95 |
| C9-Y1 | flower | 51.273 | 48.039 | 0.97 |
| E2-10Y | fruit | 44.556 | 40.717 | 0.92 |
| E2-1G | fruit | 40.476 | 37.097 | 0.93 |
| E2-2G | fruit | 44.682 | 41.536 | 0.95 |
| E2-3G | fruit | 48.985 | 45.647 | 0.94 |
| E2-4GP | fruit | 44.225 | 40.886 | 0.93 |
| E2-4GR | fruit | 47.205 | 42.994 | 0.94 |
| E2-4Y | fruit | 46.371 | 42.752 | 0.94 |
| E2-5GP | fruit | 39.367 | 36.040 | 0.93 |
| E2-5GR | fruit | 50.017 | 46.512 | 0.96 |
| E2-5Y | fruit | 45.552 | 41.501 | 0.93 |
| E2-6GR | fruit | 45.279 | 41.754 | 0.93 |
| E2-6Y | fruit | 47.257 | 43.556 | 0.94 |
| E2-7Y | fruit | 41.681 | 38.415 | 0.92 |
| E2-8Y | fruit | 55.974 | 51.130 | 0.91 |
| E2-9Y | fruit | 47.111 | 43.257 | 0.93 |
| E2-H1-HX | flower | 38.532 | 34.354 | 0.96 |
| E2-H1-LXH | flower | 52.163 | 48.448 | 0.96 |
| E2-H2-HU | flower | 49.186 | 45.636 | 0.94 |
| E2-H2-KL-XH | flower | 55.367 | 52.138 | 0.94 |
| E2-H2-LXH | fruit | 43.437 | 40.397 | 0.93 |
| E2-Y1 | flower | 69.198 | 63.383 | 0.95 |
| E2-Y2 | fruit | 43.378 | 39.564 | 0.94 |
| JH-10Y | fruit | 45.463 | 42.283 | 0.92 |
| JH-4Y | fruit | 50.622 | 46.818 | 0.94 |
| JH-7Y | fruit | 44.007 | 40.847 | 0.94 |
| JH-8Y | fruit | 58.550 | 55.106 | 0.93 |
| JH-9Y | fruit | 64.347 | 60.634 | 0.93 |
| JH-H1-HL | flower | 46.484 | 43.492 | 0.95 |
| JH-H1-SH | flower | 40.869 | 38.069 | 0.96 |
| JH-H3-BK | flower | 33.785 | 30.066 | 0.95 |
| JH-H3-HO | flower | 53.937 | 50.106 | 0.96 |
| JH-Y1 | flower | 54.646 | 51.242 | 0.96 |
| RS-FB-1 | flower | 50.792 | 47.444 | 0.97 |

**Reference**

Argout, X., Salse, J., Aury, J.-M., Guiltinan, M.J., Droc, G., Gouzy, J., et al. (2011). The genome of *Theobroma cacao*. *Nature Genetics* 43**,** 101-108.

Daccord, N., Celton, J.-M., Linsmith, G., Becker, C., Choisne, N., Schijlen, E., et al. (2017). High-quality *de novo* assembly of the apple genome and methylome dynamics of early fruit development. *Nature Genetics* 49**,** 1099-1106.

Denoeud, F., Carretero-Paulet, L., Dereeper, A., Droc, G., Guyot, R., Pietrella, M., et al. (2014). The coffee genome provides insight into the convergent evolution of caffeine biosynthesis. *Science* 345**,** 1181-1184.

Garcia-Mas, J., Benjak, A., Sanseverino, W., Bourgeois, M., Mir, G., Gonzalez, V.M., et al. (2012). The genome of melon (*Cucumis melo* L.). *Proceedings of the National Academy of Sciences* 109**,** 11872-11877.

Lin, Y., Min, J., Lai, R., Wu, Z., Chen, Y., Yu, L., et al. (2017). Genome-wide sequencing of longan (*Dimocarpus longan* Lour.) provides insights into molecular basis of its polyphenol-rich characteristics. *Gigascience* 6**,** 1-14.

Ming, R., Hou, S., Feng, Y., Yu, Q., Dionne-Laporte, A., Saw, J.H., et al. (2008). The draft genome of the transgenic tropical fruit tree papaya (*Carica papaya* Linnaeus). *Nature* 452**,** 991-996.

Myburg, A.A., Grattapaglia, D., Tuskan, G.A., Hellsten, U., Hayes, R.D., Grimwood, J., et al. (2014). The genome of *Eucalyptus grandis*. *Nature* 510**,** 356-362.

Ouyang, S., Zhu, W., Hamilton, J., Lin, H., Campbell, M., Childs, K., et al. (2006). The TIGR rice genome annotation resource: Improvements and new features. *Nucleic Acids Research* 35**,** D883-D887.

Schmutz, J., Cannon, S.B., Schlueter, J., Ma, J., Mitros, T., Nelson, W., et al. (2010). Genome sequence of the palaeopolyploid soybean. *Nature* 463**,** 178-183.

Swarbreck, D., Wilks, C., Lamesch, P., Berardini, T.Z., Garcia-Hernandez, M., Foerster, H., et al. (2007). The *Arabidopsis* information resource (TAIR): Gene structure and function annotation. *Nucleic Acids Research* 36**,** D1009-D1014.

Teh, B.T., Lim, K., Yong, C.H., Ng, C.C.Y., Rao, S.R., Rajasegaran, V., et al. (2017). The draft genome of tropical fruit durian (*Durio zibethinus*). *Nature Genetics* 49**,** 1633-1641.

The French–Italian Public Consortium for Grapevine Genome, C., Jaillon, O., Aury, J.-M., Noel, B., Policriti, A., Clepet, C., et al. (2007). The grapevine genome sequence suggests ancestral hexaploidization in major angiosperm phyla. *Nature* 449**,** 463.

Tuskan, G.A., Difazio, S., Jansson, S., Bohlmann, J., Grigoriev, I., Hellsten, U., et al. (2006). The genome of black cottonwood, *Populus trichocarpa* (Torr. & Gray). *Science* 313**,** 1596-1604.

Wang, K., Wang, Z., Li, F., Ye, W., Wang, J., Song, G., et al. (2012). The draft genome of a diploid cotton *Gossypium raimondii*. *Nature Genetics* 44**,** 1098-1103.

Wang, X., Xu, Y., Zhang, S., Cao, L., Huang, Y., Cheng, J., et al. (2017). Genomic analyses of primitive, wild and cultivated citrus provide insights into asexual reproduction. *Nature Genetics* 49**,** 765-772.

Yuan, Z., Fang, Y., Zhang, T., Fei, Z., Han, F., Liu, C., et al. (2018). The pomegranate (*Punica granatum* L.) genome provides insights into fruit quality and ovule developmental biology. *Plant Biotechnology Journal* 16**,** 1363-1374.
